# Landscape of small nucleic acid therapeutics: moving from the bench to the clinic as next-generation medicines

**DOI:** 10.1038/s41392-024-02112-8

**Published:** 2025-03-10

**Authors:** Mohan Liu, Yusi Wang, Yibing Zhang, Die Hu, Lin Tang, Bailing Zhou, Li Yang

**Affiliations:** Department of Biotherapy, Cancer Center and State Key Laboratory of Biotherapy, West China Hospital, Sichuan University, Chengdu, 610041 China

**Keywords:** Drug discovery, Medical research

## Abstract

The ability of small nucleic acids to modulate gene expression via a range of processes has been widely explored. Compared with conventional treatments, small nucleic acid therapeutics have the potential to achieve long-lasting or even curative effects via gene editing. As a result of recent technological advances, efficient small nucleic acid delivery for therapeutic and biomedical applications has been achieved, accelerating their clinical translation. Here, we review the increasing number of small nucleic acid therapeutic classes and the most common chemical modifications and delivery platforms. We also discuss the key advances in the design, development and therapeutic application of each delivery platform. Furthermore, this review presents comprehensive profiles of currently approved small nucleic acid drugs, including 11 antisense oligonucleotides (ASOs), 2 aptamers and 6 siRNA drugs, summarizing their modifications, disease-specific mechanisms of action and delivery strategies. Other candidates whose clinical trial status has been recorded and updated are also discussed. We also consider strategic issues such as important safety considerations, novel vectors and hurdles for translating academic breakthroughs to the clinic. Small nucleic acid therapeutics have produced favorable results in clinical trials and have the potential to address previously “undruggable” targets, suggesting that they could be useful for guiding the development of additional clinical candidates.

## Introduction

**Fig. 1:**
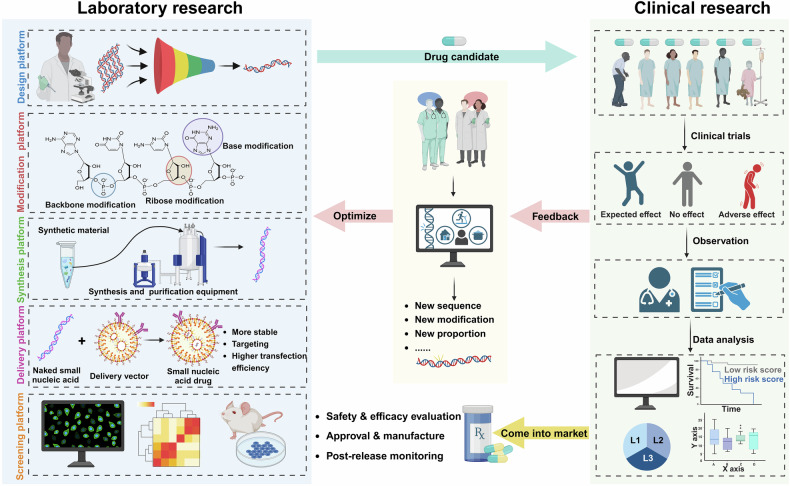
Translating small nucleic acid-based pharmacotherapy from bench to clinic and back. The development of small nucleic acid drugs begins with the design of potential nucleic acid sequences tailored to target specific diseases. Researchers then screen these sequences to identify the most effective candidates. Once optimal sequences are identified, they undergo chemical modifications to increase their stability and reduce their toxicity. These modifications optimize the drug’s pharmacokinetic properties, increasing the viability of the drug for therapeutic use. The modified small nucleic acid sequences are then encapsulated in delivery vectors. This step is crucial, as it further increases the drug’s stability in vivo and ensures that it reaches the specific target organ effectively. Extensive in vitro and in vivo evaluations have been conducted to assess the efficacy, safety, and pharmacodynamics of the drug. These evaluations help refine drug candidates before they proceed to clinical trials. Promising drug candidates then enter clinical trials, where many patients are recruited. Medical staff meticulously recorded the responses of different subjects to the candidate drugs, noting any therapeutic effects or adverse reactions. The clinical data obtained are analyzed by researchers, often with the assistance of artificial intelligence, to obtain deeper insights into the drug’s performance. This analysis helps researchers understand the drug’s efficacy, safety profile, and potential areas for improvement. The interpreted data are fed back to the laboratory for further optimization of the drug. Researchers may tweak the chemical structure, modify the delivery vector, or make other adjustments to enhance the drug’s performance. This iterative process continues, ensuring that each cycle brings the drug closer to its optimal form. This cycle of design, testing, feedback, and optimization is repeated until the drug meets the desired standards of efficacy and safety, ultimately leading to its approval and use in clinical settings

Gene therapies were initially used to treat diseases during the 1960s and early 1970s, and major biological and technological breakthroughs led to the development of several safe and effective platforms.^1^ Nucleic acid therapeutics use engineered sequences of nucleotides to selectively modulate gene expression.^2^ The most prominent nucleic acid drugs are those based on antisense oligonucleotides (ASOs), aptamers, short interfering RNAs (siRNAs) and microRNAs (miRNAs), which enhance the effects and delivery of drug materials, revolutionize precision medicine and increase the efficacy of existing pharmaceuticals.^3,4^ Oligonucleotides can be designed to target specific mRNAs and are capable of treating or managing a broad spectrum of diseases.^5^ The mechanism of action of these drugs differs from that of traditional drugs, which in some cases helps to prevent drug resistance, especially in cancer treatment. The efficacy of these drugs can be precisely controlled by altering their sequences, structures, or chemical modifications, and their flexibility allows the medication to be tailored according to the specific needs of the disease. In recent years, researchers have developed various delivery platforms to improve the stability and bioavailability of oligonucleotide drugs, thereby enhancing their therapeutic effects.^6^ Currently approved nucleic acid therapeutics and drugs assessed in clinical trials are valuable for patients who previously had limited treatment options (Fig. 1).

ASO technology, which originated from a 1978 study, uses synthetic strands of 15 or 20 nucleotides to complement a short RNA sequence of the Rous sarcoma virus directly.^7^ Based on Watson–Crick base pairing, the concept of treating a target RNA as a receptor for an ASO was proposed. Following this important finding, commercial companies focused on developing antisense therapeutics in the late 1980s to work against ‘undruggable’ receptors. High-throughput screening systems were established to identify optimal RNA binding sites for ASOs, leading to advances in ASO design. Currently, ASO drugs have been designed using this strategy, and further chemically modified ASOs have been used to enhance their pharmacological properties and passive tissue-targeting abilities.^8^ Additionally, RNA interference (RNAi) was first described in 1998 as a process of sequence-specific gene silencing initiated by double-stranded RNA (dsRNA), sparking great interest from drug developers for its potential to suppress the expression of specific genes.^9–11^ In 2001, Elbashir et al. were the first to use siRNAs to silence the expression of different genes in mammalian cell lines. These researchers generated siRNAs against sea pansy (*Renilla reniformis*, RL) luciferase and siRNAs against two sequence variants of firefly (*Photinus pyralis*, GL2 and GL3) luciferase.^12^ The first proof-of-principle experiment in which siRNAs could be utilized for the treatment of a disease in an in vivo model was performed in 2003. Research has shown that an intravenous injection of a Fas siRNA specifically reduces Fas mRNA levels and protects mice from fulminant hepatitis.^13^ The Nobel Prize in Medicine and Physiology was awarded to Andrew Fire and Craig Mello in 2006 for their discovery of RNA interference (RNAi). Since then, the development of delivery systems has broadened the potential applications of RNAi, particularly in rare diseases and cancers. The first human trial started in 2008, and the data were published in 2011. A phase 1 study (NCT00689065) was designed for the systemic administration of an siRNA to patients with solid tumors via a targeted nanoparticle delivery system, and the results revealed specific gene inhibition.^14^ However, drug developers have struggled to place siRNA therapies on the market quickly for clinical use, and the first globally approved siRNA drug for marketing was the therapeutic patisiran (ONPATTRO™), which is used for the treatment of hereditary transthyretin (hATTR)-mediated amyloidosis.^15^ Since then, interest in siRNA therapies has increased, even surpassing interest in ASO therapies.

Compared with traditional small-molecule and protein drugs, nucleic acid drugs have unique advantages. Fundamentally, the main targets of traditional small-molecule drugs and antibody drugs are proteins, but only 1.5% of the human genome can encode proteins, 80% of which are undruggable targets for traditional drugs. Unlike traditional drugs, nucleic acid drugs regulate target expression by binding to related nucleic acids, which means that nucleic acid drugs are theoretically applicable to any therapeutic target. Furthermore, designing a nucleic acid drug that binds to the target gene is relatively easy once the base sequence of the target gene is known. Finally, because of the simple nucleotide synthesis process and low cost, the research and development of nucleic acid drugs are quite accessible.

**Fig. 2:**
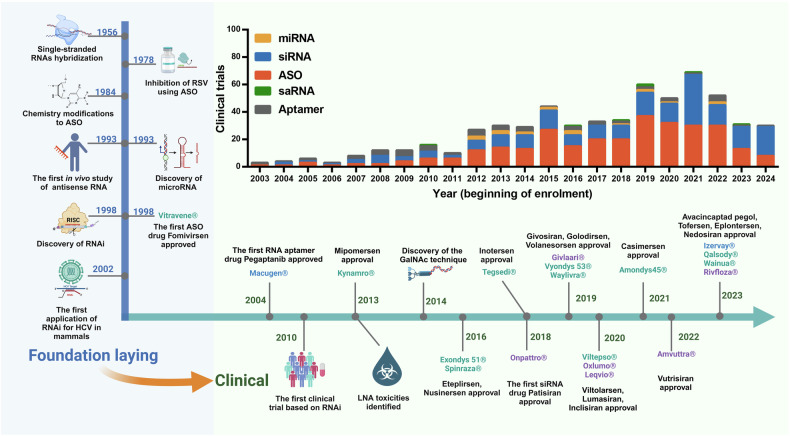
Timeline of milestones from the discovery of small nucleic acid drugs to their clinical use. The development of nucleic acid therapeutics has been marked by considerable technological advances, pivotal drug approvals, and notable setbacks. A timeline highlighting crucial breakthroughs depicts the evolution from foundational research to clinical application. As the performance of antisense oligonucleotides (ASOs) has improved, the scope of therapeutic opportunities has broadened, now encompassing both rare and common diseases and virtually any delivery route. The bar graph illustrates the number of clinical trials for small activating RNAs (saRNAs), microRNAs (miRNAs), small interfering RNAs (siRNAs), and antisense oligonucleotides (ASOs) over the years. Each bar represents the beginning of enrollment recorded in the ClinicalTrials.gov database, providing a clear picture of the growing momentum in nucleic acid therapeutic research (up to the middle of 2024). RSV respiratory syncytial virus, RNA RNA interference, HCV hepatitis C virus, LNA locked nucleic acid, GalNAc N-acetylgalactosamine

In recent years, small nucleic acid-based therapeutic modalities have expanded the platforms available for clinical oligonucleotide drug development. ASO drugs were first used on the market to control the expression of target genes. Chemical modification of ASOs is necessary for their stability and efficacy; however, the phosphorothioate or polyethylene glycol linkage in the backbone of ASOs can increase their binding affinity to unintended proteins, which is associated with toxicity. siRNA drugs with less modified linkages in the backbones can be developed as alternatives to ASO drugs. The first siRNA drug, patisiran, was approved by the FDA in 2018, and the second siRNA drug, givosiran, was approved in 2019.^15,16^ siRNA drugs have entered a rapid stage of development. After 20 years of research, the commercial potential and clinical value of small nucleic acid drugs have been proven (Fig. 2). In this review, we bridge the gap between laboratory research and the clinical implementation of small nucleic acid therapies, emphasizing how these technologies have evolved and are now poised for practical therapeutic use. Moreover, we focus on summarizing the latest advancements in drug delivery systems, particularly in the areas of nucleic acid chemical modification and nanoparticle carrier systems, highlighting how these improvements increase delivery efficiency. We also seek to identify the challenges and future directions in the field, discussing how these advancements could revolutionize the treatment of previously untreatable conditions.

## Therapeutically relevant nucleic acids

### Classification of therapeutically relevant nucleic acids

**Fig. 3:**
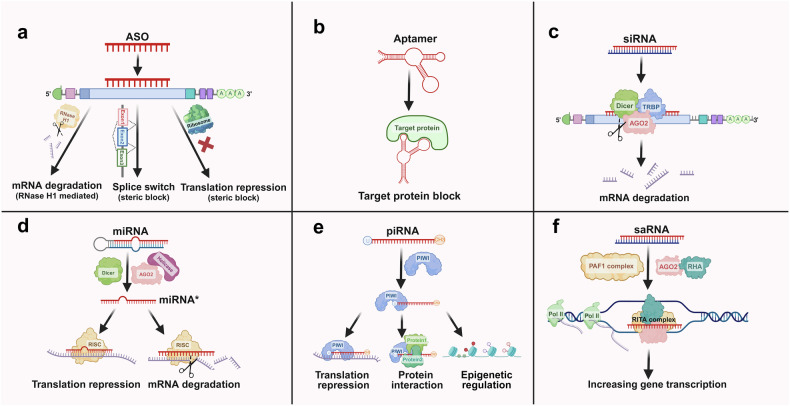
The functional mechanisms of four small nucleic acids. **a** ASOs: ASOs are short synthetic nucleic acid sequences that bind to complementary mRNA molecules. This binding can block the translation process or promote the degradation of the mRNA, thereby reducing the expression of the targeted protein. **b** Aptamers: Aptamers are short, single-stranded nucleic acids that bind specific targets with high affinity and specificity. They function by folding into unique three-dimensional structures, allowing them to interact with proteins, small molecules, or cells effectively. **c** siRNAs: siRNAs are double-stranded RNA molecules that guide the RNA-induced silencing complex (RISC) to complementary mRNA targets. This interaction leads to the cleavage and degradation of mRNAs, resulting in the silencing of specific genes. **d** miRNAs: miRNAs are endogenous, single-stranded RNA molecules that regulate gene expression posttranscriptionally. They bind to complementary sequences in the 3’ untranslated regions (UTRs) of target mRNAs, typically causing translational repression or mRNA degradation, thus modulating the expression of multiple genes. **e** PIWI-interacting RNAs (piRNAs) are small, single-stranded RNA molecules, approximately 24–30 nucleotides long. They interact with PIWI proteins to regulate gene expression, silence transposable elements, and maintain genome stability in germline cells. **f** saRNAs: saRNAs are synthetic short RNA molecules designed to upregulate gene expression. They bind to specific promoter regions of target genes, recruiting transcriptional activators or altering the chromatin structure to increase transcriptional activity, thereby increasing the production of specific proteins

According to their molecular mechanisms, therapeutically relevant nucleic acids can be roughly categorized into eight categories: aptamer, siRNA, miRNA, ASO, small activating RNA (saRNA), PIWI-interacting RNA (piRNA), messenger RNA (mRNA) and plasmid DNA (pDNA). In this review, we focus on small nucleic acid therapeutics, in which mRNA and pDNA are not included (Fig. 3).

#### ASOs

ASOs are short synthetic single-stranded oligonucleotides of 18–30 nucleotides in length and regulate gene expression by blocking the function of RNAs. Two major mechanisms for ASOs to exert their functions have been identified: RNase H1-dependent cleavage and steric hindrance.^17^ In the RNase H1-dependent mechanism, the ASO always contains a central contiguous sequence of 8–10 deoxynucleotides sandwiched between two RNA flanking regions, termed the ‘gapmer’ pattern.^6,18^ After binding, RNase H1 cleaves the target RNA strand, including the mRNA and pre-mRNA strands, approximately 7–10 nucleotides from the 5′-end of the duplex region since RNase H1 is robustly active in both the nucleus and the cytoplasm.^19,20^ In the RNase H1-independent mechanism, ASOs bind to target RNAs with high affinity, causing steric hindrance to alter gene expression by masking specific sequences rather than cleaving RNA.^5^ After the ASO binds to the initiation codon of an mRNA, steric interference prevents ribosome binding, thus inhibiting mRNA translation. Additionally, ASOs are also used to increase target gene expression.^21^ When multiple open reading frames (ORFs) exist in an mRNA, binding of the ASO to the initiation codon of the upstream ORF can alleviate translation inhibition of the main ORF, thereby activating the expression of the target protein, which is typically suppressed by the ORF.^22^ Moreover, ASOs can activate gene expression by masking premature termination codons or the exon junction complex, thereby leading to target mRNA decay.^23,24^ Importantly, ASOs can also alter the biological properties of proteins by binding to pre-mRNAs to make the splicing signal invisible to the spliceosome, thereby modulating splicing decisions such as exon skipping and exon inclusion.^25,26^

Single-stranded ASOs are unstable against intracellular nuclease degradation and are strictly complementary to target RNAs. These properties indicate that the ability to optimize the properties of ASOs via sequence variation is limited. Thus, chemically modified nucleotides or specific sequences are incorporated to increase the binding affinity and resistance to nuclease degradation of the ASOs.^27^ In addition, ASOs tolerate more extensive chemical modifications in the gapmer without abrogating the activity of RNase H1.^28^ The detailed chemical modifications of ASOs will be introduced in the next section.

#### Aptamers

Aptamers are short single-stranded nucleotides with unique tertiary structures, typically 20 to 100 nucleotides of DNA or RNA.^29^ Aptamers have diverse secondary structures, such as hairpins, internal loops, bulges, pseudoknots, and G tetramers.^30^ Aptamers can recognize, bind and subsequently block target molecules with high affinity, working like antibodies.^31^ Compared with in vivo synthesized antibodies, aptamers have the advantages of a low cost, low immunogenicity, a short synthesis time and high specificity.^32^ Additionally, due to their flexible nature, aptamers can be designed to bind even hidden epitopes, which is impossible for antibodies.^33^ Generally, high-affinity aptamers are selected from random libraries using an in vitro procedure called systematic evolution of ligands by exponential enrichment (SELEX).^34^ The basic process of SELEX is a repeated cycle of four steps: incubation, binding, elution of the bound target and amplification. First, a random oligonucleotide sequence library is incubated with the target molecule. During incubation, some sequences bind to the target molecule, whereas others bind weakly or do not react. Then, through partitioning and washing, lower affinity aptamers and unbound sequences are removed from the solution, whereas bound aptamer molecules are eluted. The eluted sequences are subsequently amplified for the next round of selection. After 5–15 rounds, a rich pool of aptamer candidates can be obtained for experimental evaluation and further optimization to obtain the final mature aptamers. However, traditional black box approaches such as SELEX run the risk of being unable to develop the best aptamer for a specific target.^34^ Thus, researchers have developed more advanced methods for consistently producing high-performance aptamers. For example, Gotrik et al. designed the microfluidic device SELEX and high-throughput sequencing SELEX, and the results revealed that after these alterations, higher-affinity aptamers can be obtained after far fewer rounds of selection relative to conventional methods.^35^ Capillary electrophoresis allows separation in free solution, and its participation in SELEX can greatly reduce the selection time and selection cost.^36^ Ishida et al. presented a novel method, RNA aptamer Ranker (RaptRanker), to identify optimal aptamers from HT-SELEX data by scoring and ranking. Through an evaluation, they proved that the performance of RaptRanker was superior to that of conventional methods, such as frequency, enrichment and MPBind.^37^

#### siRNAs

siRNAs are double-stranded RNA molecules consisting of 20–25 base pairs, with two nucleotides overhanging at the 3’ ends. Each molecule possesses a 5’ phosphate and a 3’ hydroxyl group.^12,38^ There are two strands of siRNA, one is guide strand (also called antisense strand), which can guide nuclease to cut the target gene; the other one is passenger strand (also known as sense strand). After the introduction of double-stranded siRNA into cytoplasm, the Argonaute 2 protein (AGO2) clears the passenger strand, releasing the guide strand, forming an RNA-induced silencing complex (RISC) with Dicer and transactivation response RNA binding protein (TRBP).^39–43^ A success RNAi initiation is owing to the correct selection of guide sense by Dicer, possessing ability of asymmetrical recognition, since the passenger sense is incapable in RNAi activation.^40,44^ TRBP maintains a proper orientation for the guide strand to bind to AGO2 because of its high flexibility. After the guide strand binding with specific mRNA, the target mRNA is cleaved, a process mediated by AGO2 that occurs between the 10th nucleotide and the 11th nucleotide from the 5′-most of paired sequences.^45–48^ Broken mRNA is unable to maintain the structural stability and subsequently degraded, resulting in target gene downregulation.

Initially, siRNA sequence selection was based on experimental insights, but bioinformatic methods are now used to aid in siRNA design. Key principles in siRNA design include targeting the coding sequence (CDS) or 3’ untranslated regions (UTR) and avoiding the 5’ UTR; ensuring no complementarity with nontarget mRNAs or homology with sequences from other species^49^; preferring 21-nt siRNAs with 2-nt 3′ overhangs and necessary 5′ phosphates^50,51^; avoiding overly long dsRNAs to prevent mRNA degradation failure and apoptosis^52–54^; preventing secondary structure formation in the guide strand^55^; maintaining a G-C content between 30% and 52%^56^; ensuring strand asymmetry, where the 5′ end of the guide strand is less stable and having U or A^57,58^; avoiding U-rich or GU-rich sequences in the guide strand to reduce immunogenicity^59–62^; and considering deoxyribonucleotide (TT) substitutions for 3′ overhangs to lower the synthesis costs.^47^

#### miRNA

The mechanism by which miRNAs silence gene expression is similar to that of siRNAs. Several steps are needed for endogenous miRNAs to progress from primary miRNAs (pri-miRNAs) to mature miRNAs. First, pri-miRNAs are transcribed from miRNA genes with RNA polymerase II in the nucleus.^63^ DGCR8 helps cleave pri-miRNAs at approximately 11 bp from the junction with Drosha, creating hairpin pre-miRNAs with 60 to 70 nucleotides, called pre miRNAs.^64^ Then, these pre-miRNAs are exported to the cytoplasm by the EXP5 complex and further processed into shorter double-stranded duplexes by Dicer.^65^ One strand (miRNA*) of the duplexes is stabilized in Argonaute to form the miRISC and the other strand (miRNA) is expelled.^63,66–68^ Finally, miRISC targets mRNAs, typically at the 3′-UTR, through seed sequence hybridization, leading to mRNA translational inhibition or decay. On the one hand, guide strand miRNAs that have perfect complementary pairing with their mRNA targets induce mRNA cleavage and subsequent degradation; on the other hand, miRNAs that have imperfect complementary pairing with their mRNA target repress mRNA translation through steric hindrance, resulting in target gene silencing.^69^ Unlike rigid siRNAs, a single miRNA duplex can simultaneously bind to different mRNAs, regulating the expression of multiple proteins, as more than half of the human genome contains numerous miRNA binding sites.^70^ Although abundant microRNAs are considered negative regulatory noncoding RNAs (ncRNAs) that function in the cytoplasm, emerging evidence shows that there are also some miRNAs in the nucleus.^71–73^ More unexpectedly, Xiao et al. reported a set of miRNAs located in the nucleus possessing gene-activating function. In their research, they found that miR-24-1, a member of theses miRNAs, can bind to enhancers of RNA transcripts, increasing expression of the target gene.^74^ The most commonly used miRNA drugs in the clinic are still miRNA mimics, whose sequences mirror those of natural endogenous miRNAs to downregulate the expression of target genes.

Since the sequences of miRNA mimics are exactly the same as those of natural miRNAs, modifying their sequences is challenging. However, key principles in miRNA mimic design have been identified. First, the mimics should involve two incompletely paired strands, as double-stranded miRNA mimics exhibit significantly greater efficacy in gene silencing than single-stranded mimics do, by approximately 100–1000-fold.^75^ Additionally, variations in the length of pre-miRNAs may affect Dicer cut site selection, potentially altering the miRNA seed sequence and influencing guide strand selection.^76^ Ideal length for miRNA mimics is 22 base pairs, matching that of mature endogenous miRNA duplexes. This length not only enhances the ease of delivery and reduces costs but also ensures optimal efficacy. Moreover, the seed and 3′ regions of synthesized miRNAs should be enriched with AU sequences to prevent strong binding between the target RNAs and the 3′ regions of guide strands.^77,78^

#### piRNAs

piRNAs are a class of single-stranded small noncoding RNAs (ncRNAs) with a length of 26–31 nucleotides.^79^ The concept of piRNAs was introduced in July 2006. Several research groups almost simultaneously reported that a new class of ncRNAs typically binds to PIWI proteins, resulting in the silencing of transposable elements, which is distinct from the mechanisms for siRNAs and miRNAs. PIWI, a subgroup of Argonaute proteins, is expressed mainly in the reproductive system.^80–83^ Endogenous piRNAs are processed from piRNA-coding sequences, which are predominantly grouped into 0.4–73.5 kilobase clusters occurring in the intergenic regions of chromosomes with highly uneven distributions.^84^ piRNA clusters are processed into mature piRNAs through several steps. The piRNA clusters are transcribed to primary piRNAs (pri-piRNAs) by RNA polymerase II in the nucleus, similar to the process of miRNAs. Single-stranded pri-piRNAs are subsequently transported to the Yb body in the cytoplasm for further slicing with Zucchini (Zuc).^85^ The sliced 5′ fragment subsequently binds to the PIWI proteins, forming an intermediate-piRNA-PIWI complex. After the 3′ end is trimmed to the optimal length and the 2′-hydroxy group at the 3′-end is methylated, the mature piRNA-PIWI complex migrates back into the nucleus to block target gene transcription.^86^ In addition to the primary processing pathway described above, mature piRNAs can be amplified through a “ping-pong” cycle. In addition to PIWI proteins, mature piRNAs can also bind to AGO3 (sense transcription) or AUB (antisense transcription). The resulting piRNA-AGO3/AUB complex can target pri-piRNAs and cut them into new mature piRNAs for the next cycle.^87^ Numerous studies have shown that piRNAs can regulate the differentiation and development of germ cells through transposon inhibition.^88–93^ In general, three major pathways by which piRNAs regulate gene expression have been identified. piRNAs can form a piRNA-induced silencing complex (piRISC) with PIWI proteins, resulting in transcriptional gene silencing in the nucleus and posttranscriptional gene silencing in the cytoplasm.^94^ In the PIWI cleavage process, mismatches to any target nucleotide can be tolerated, including those flanking the scissile phosphate.^95^ Additionally, piRISC can also bind to proteins to promote interactions with multiple proteins.^96^

Although most studies of piRNAs are focused on mechanistic research and rarely on gene regulation therapies in preclinical studies or clinical trials, growing evidence indicates that piRNA predominantly regulates the occurrence and progression of cancer cells, particularly in terms of proliferation, migration, metastasis, and apoptosis.^97^ piRNAs act as oncogenes or tumor suppressors by regulating factors in tumor-related signaling pathways. For example, piR-651 is upregulated in various human solid cancers, where its overexpression promotes cell proliferation and invasion, suppresses apoptosis, and decreases G0/G1 phase arrest, accompanied by increased expression of oncogenes such as MDM2, CDK4, and Cyclin D1.^98^ In contrast, piR-823 is expressed at significantly lower levels in gastric cancer tissues compared to non-cancerous tissues. Additionally, piR-823 interacts with PINK1, enhancing its ubiquitination and proteasomal degradation, thereby suppressing mitophagy during tumor development.^99,100^ Thus, targeting piRNAs presents a promising therapeutic strategy. Emerging studies suggest that piRNAs could be leveraged to modulate pathways that contribute to tumorigenesis, presenting new avenues for therapeutic intervention. For example, the downregulation of PIWIL1 and piR-DQ593109 enhanced BTB permeability via the MEG3/miR-330-5p/RUNX3 axis, providing valuable insights for glioma therapy.^101^ Another successful application is the upregulated expression of piRNA-36712, which shows a synergistic anticancer effect when combined with chemotherapeutic agents in breast cancer cells.^102^ However, delivering piRNA in a manner that is specific to organs remains a challenge to minimize off-target effects. Further research is needed to fully characterize the mechanisms through which piRNAs operate in different cancer types. Exploring the therapeutic potential of piRNA-based treatments, including the design of piRNA mimics or inhibitors, could lead to innovative cancer therapies with improved specificity and reduced side effects. Collectively, piRNAs offer a promising new frontier in cancer research, with the potential to be developed into targeted therapies that address both the prevention and treatment of cancer by leveraging their unique role in maintaining genomic stability and regulating key oncogenic pathways.

#### saRNAs

saRNAs are double-stranded RNAs consisting of 21 nucleotides, with 2 nucleotides overhanging at the 3’ end, which can increase the expression of target genes by RNA activation (RNAa).^103,104^ After introduction into the cytoplasm, the saRNA duplex binds to AGO2 and unwinds under the action of RNA helicase A (RHA), following an import into the nucleus mediated by importin-8.^105–109^ The guide strand then leads the complex to bind with target promoters, recruiting the polymerase-associated factor 1 (PAF1) complex to assemble the RNA-induced transcriptional activation (RITA) complex.^110^ Finally, the RITA complex initiates transcription and productive elongation by RNA polymerase II, thus increasing the expression of target proteins.^111^

The selection of genomic regions is vital in the sequence design of saRNAs. There should be sequences in the saRNAs which can complementary with the promoters or 3’ terminus of target genes.^112^ The target region should not be identical to other sequences to avoid off-target effects (OTEs) caused by sequences; the target region should not contain CpG islands or DNA hypermethylation to avoid OTEs caused by special nucleotides.^113^ Furthermore, every nucleotide is highly important for saRNAs since mutation of the seed region can influence RNAa levels and can even lead to complete dysfunction.^114^ Additionally, single-stranded saRNAs have no effect on gene expression activation, indicating that the presence of an intact duplex RNA is essential.^115^

Compared with the downregulation of genes triggered by siRNAs and miRNAs, the activation of gene expression induced by saRNAs lasts much longer, approximately 2 weeks, than that induced by other agents and is 7 days long.^113^ However, a greater concentration (nM) of saRNA therapeutics is required to achieve therapeutic effects, whereas siRNAs can act at a relatively low concentration (pM–nM).^116^ Compared with mRNAs, saRNAs offer advantages in mass production because of their lower nucleotide count, resulting in substantially lower costs. Moreover, the smaller size of saRNAs may pose fewer delivery challenges than those posed by the larger size of mRNA molecules. Additionally, saRNAs tap into the gene regulatory machinery of endogenous cells to activate gene expression, reduces the risk of detrimental overexpression and immunogenicity, a common challenge encountered with mRNAs.^117^

In summary, although some differences exist among the six types of small nucleic acid therapeutics, they all make great efforts to rescue the disordered gene expression of targets that were previously considered undruggable. Although the rational design of a nucleic acid sequence can increase its potency, chemical modification is a much more powerful strategy.

### Modification of nucleic acids

**Fig. 4:**
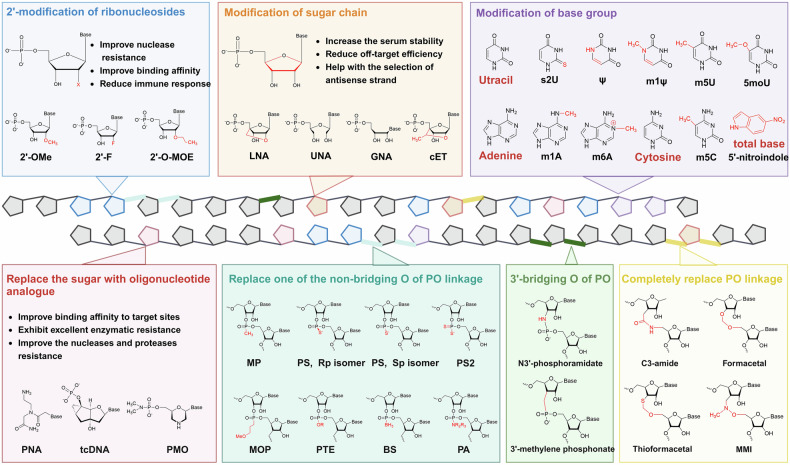
Chemical modifications used for small nucleic acids. Common modifications can be divided into three categories: ribosome modifications, nucleobase group modifications and backbone modifications. Modifications of ribosomes are always performed on the 2’ ends of ribose sugars (blue box) and sugar chains (orange box). In modifications of the base group, uracil, adenine and cytosine are the common objects (purple box), and the ribose sugar can be completely replaced (red box). The PO linkage of the backbone can be changed, including the nonbridging O atom (cyan box), 3’-bridging O atom (green box) and total PO linkage (golden box), to reduce the net anionic charge of small nucleic acids

Since unmodified naked nucleic acids that are intravenously injected into the target tissue can be rapidly degraded by RNases and cleared from the blood, chemical modification is crucial for generating effective nucleic acid drugs.^118^ The key goals for chemical modification include increasing the binding of small nucleic acids with target sequences, enhancing nuclease stability, optimizing pharmacokinetic characteristics, and minimizing side effects.^119,120^ To date, a lot of effort has been made in modification of small nucleic acids (Fig. 4).^121^

#### Structural modification of nucleic acids

The structure of ribonucleotides can be altered by chemically modifying nucleotide bases, sugar moieties, or nucleosides. Widely used 2’-sugar modifications include 2ʹ-fluoro (2ʹ-F), 2ʹ-O-methyl (2ʹ-OMe) and 2ʹ-O-(2-methoxyethyl) (2ʹ-O-MOE), all of which have been proven to be effective and accessible to the market.^122–126^ In addition to adding modifications at the 2’ position of ribose, changing its structure is also feasible. RNAs with ribose structural modifications include constrained ethyl (cEt), glycol nucleic acids (GNAs), locked nucleic acids (LNAs; also known as 2′,4′-bridged nucleic acids, BNAs), nucleosides and tricyclo-DNA (tcDNA) and unlocked nucleic acids (UNAs). cEt nucleosides are similar to LNAs in structure with an additional methyl group.^127^ GNAs are acyclic nucleic acid analogs, which can result in greater thermal destabilization of the duplex structure.^128^ There is a methylene bridge to connect the 4′-carbon with 2′-oxygen of LNAs, which can both increase the serum stability and reduce the off-target effects.^129^ UNAs are 2′,3′-seco-RNAs that always used to disrupt the stability of double-stranded RNA.^130^ Another common strategy involves modifying the base group of nucleotides, such as replacing uracil with pseudouridine (Ψ),^131^ N1 methylpseudouridine (m1 Ψ),^132^ 5-methyluridine (m5U),^133^ 5-methoxyuridine (5moU)^134^ or 2-thiouridine (s2U)^135^; using N1-methyladenosine (m1 A)^136^ or N6-methyladenosine (m6 A)^137^ instead of adenine; and methylating cytosine to 5-methylcytidine (m5C).^138^ Moreover, the base group can be directly replaced with a nitroindole, an universal base to decrease undesired off-target effects.^139^ Through the addition of oligonucleotide analogs to nucleic acids, such as tcDNA, a conformationally constrained oligonucleotide analog^140^; PMO, which replaces the pentose sugar with a morpholine ring; phosphate^141^; peptide nucleic acids (PNAs), enhance both the selectivity and thermal stability of the compounds to increase the binding to target RNAs.^142^

#### Backbone modifications

Reducing the net anionic charge of oligonucleotide drugs and increase the delivery efficiency.^143^ A common strategy is to replace one of the nonbridging oxygen atoms of the phosphodiester bond (PO linkage) with a nonionic modification group,^137^ such as phosphorothioate (PS),^144^ methylphosphonate (MP),^145^ methoxypropyl phosphonate (MOP),^146^ phosphorodithioate (PS2),^147^ phosphoramidate (PA),^148,149^ phosphoroselenoate (PSe),^150^ phosphotriester (PTE)^151^ or boranophosphate (BS).^152^ Likewise, the 3′-bridging oxygen atoms of the PO linkage can be replaced with carbon or nitrogen to form 3′-methylene phosphonate or N3′-phosphoramidate, respectively.^153,154^ In fact, the PO linkage can be completely replaced. The trans isomers of amides preorganize sugars into C3′-endo conformations; thus, the C3-amide modification (3’-CH_2_-CO-NH-5’) increases the biological activity of nucleic acids.^155^ Methylene (methylimino) (MMI) is another nitrogen that contains an achiral four-atom linkage (3’-CH_2_N(CH_3_)-O-5’) and is completely resistant to nucleases.^156^ Apparently, the hydrophobic formacetal linkage (3’-O-CH_2_-O-5’) exhibited increased stability in all the RNA duplexes, whereas it strongly destabilized the DNA helix.^157^ Thioformacetal, which replaces the 3′-sided oxygen atom with a sulfur (3’-S-CH_2_-O-5’), increases both the stability of nucleic acids and the affinity for target RNA.^158^ Among these modifications, PS is the most commonly used. The PS linkage has chemical properties similar to those of the PO linkage, but it can greatly increase the metabolic stability of small nucleic acids and protect them from rapid degradation.^159^ In addition, there are two isomers of PS linkage, including right-handed (Rp) and left-handed (Sp), possessing different biological properties.^160^ With the development of synthetic methodologies, stereopure PSs with only one isomer are possible. However, whether nucleic acid drugs with stereopure PSs possess superior potency, efficiency and durability is still unclear since this topic has been highly debated for several years.^161–164^

In the early days of the chemical modification of nucleic acid drugs, primary agents were synthesized, and recent studies have shown that their combination with nucleic acids and the backbone has excellent potency. Wu et al. combined PS2 with 2’-OMe in a same nucleotide (MePS2), results showed enhanced resistance to nucleases degradation for siRNAs.^165^ Baker et al. suggested that introducing LNAs into splice-switching oligonucleotides, which have been modified with phosphorothioates, increases the activity of nucleic acids due to their reduced charge.^166^ Notably, though these chemical modifications showed robust efficiency and well tolerance in a broad of researches, there are some side effects occurred in the clinic.^167^ For example, PS-modified oligonucleotides lead to unexpected binding to platelets, thus forming thrombus.^168^ Moreover, LNAs can induce hepatotoxicity consistent with acute liver injury in as little as 4 days, which might be caused by two trinucleotide motifs, TCC and TGC.^169,170^ Safe and efficient chemical strategies should be a focus for the development of future nucleic acid therapeutics.

## Nucleic acid delivery system

**Fig. 5:**
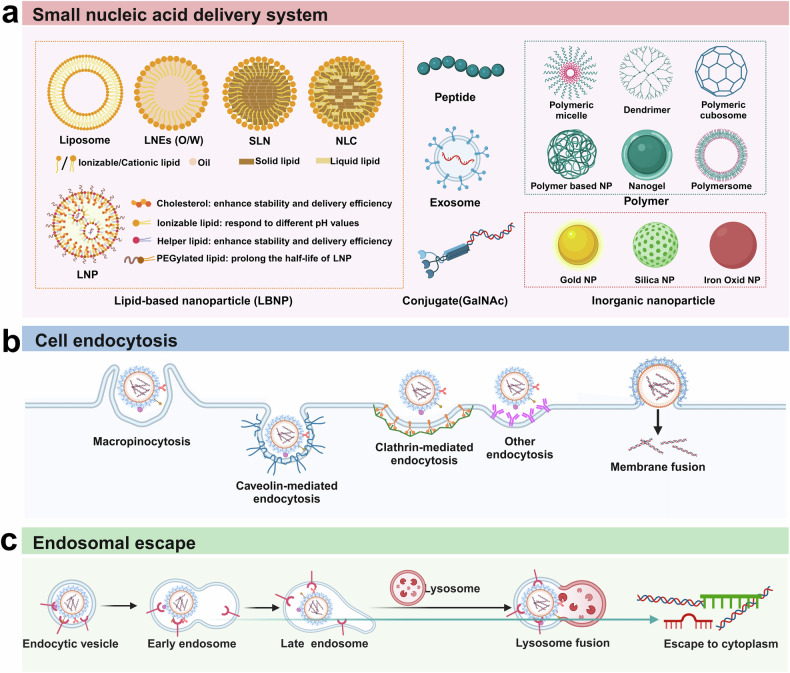
Technologies for the delivery of small nucleic acids. **a** The six types of common nonviral delivery vectors include lipid-based nanoparticles (LNPs), polymers, peptides, exosomes, conjugates (GalNAc) and inorganic nanoparticles. **b** Schematic diagram of small nucleic acid drug uptake pathways by target cells. There are four major endocytosis pathways, including macropinocytosis, caveolin-mediated endocytosis, clathrin-mediated endocytosis and direct membrane fusion. **c** Schematic diagram of endosomal escape. After internalization, endocytic vesicles develop into early endosomes and late endosomes and subsequently fuse with lysosomes to induce lysosomal degradation. Only a small fraction (usually less than 1%) of small nucleic acids can escape from the endosome to the cytoplasm

Although rational sequence design and chemical modification of nucleic acids can increase their stability and reduce OTEs, targeted delivery to specific cells and organs remains a great challenge. Ideal delivery systems are expected to possess high drug loading efficiency and sufficient safety, protect nucleic acids from rapid degradation and specific targeting. In the following sections, we describe the major nonviral delivery systems used for small nucleic acids (Fig. 5).

### Lipid-based nanoparticles (LBNPs)

LBNPs are spherical nanoparticles with favorable biocompatibility and degradability and are currently the most promising delivery carrier. LBNPs can be divided into five categories according to their compositions: liposomes, lipid nanoparticles (LNPs), lipid nanoemulsions (LNEs), solid lipid nanoparticles (SLNs) and nanostructured lipid carriers (NLCs).

#### Liposomes

Classic liposomes consist of cholesterols and amphiphilic phospholipid bilayers encapsulating an aqueous inner compartment, with sizes ranging from 100 nm to 5 μm, which is quite similar to the size of the natural cell membrane.^171,172^ The amphiphilic phospholipid has a hydrophobic tail group and a hydrophilic head group, which are oriented to the outer and inner sides in the composition of liposomes. Liposomes can be divided into 2 types according to their basic structures: unilamellar liposomes with single bilayer membranes and multilamellar liposomes containing multiple bilayer membranes.^173^ Liposomes were first discovered in 1961. Bangham and Horne reported that phospholipids dispersed in excess water could form closed vesicles composed of bimolecular membranes, and images captured with an electron microscope were published in 1964.^174^ In 1971, Gregoriadis et al. first constructed liposomes composed of phosphatidylcholine, cholesterol and diacetyl phosphate as enzyme drug carriers. The results revealed no measurable leakage of the encapsulated albumin, and most of the albumin accumulated in the liver.^175^ Since then, liposomes have been widely used as drug delivery systems.^176–178^ Yano et al. complexed siRNA-B717, which targets the human *Bcl-2* oncogene, with a novel cationic liposome, LIC-101, containing 2-O-(2-diethylaminoethyl)-carbamoyl-1,3-O-dioleoylglycerol and egg phosphatidylcholine and intravenously injected into mice with liver metastasis and subcutaneously injected into prostate cancer-bearing mice (inoculated under the skin). In both models, the complex showed strong antitumor activity.^179^ However, cationic nanocarriers are reported to induce cellular necrosis, especially in the lungs, upon systemic administration.^180^ Changing the electric charge of liposomes could be a promising strategy. Qian et al. employed hyaluronan (HA) to negate the positive charge of liposomes since HA is an anionic polysaccharide with a negative charge. Through in vitro and in vivo tests, HA-modified liposomes were shown to exhibit reduced toxicity and a comparable transfection efficiency to unmodified liposomes.^181^ In addition, Villares et al. constructed neutral liposomes with neutral 1,2-dioleoyl-*sn*-glycero-3-phosphatidylcholine (DOPC), which can simultaneously improve the tumor uptake efficiency of nucleic acid‒liposome complexes. Their results first revealed the effective inhibition of melanoma growth and metastasis in melanoma-bearing mice after systemic treatment with the protease-activated receptor-1 siRNA-DOPC complex.^182^

#### Lipid nanoparticles (LNPs)

LNPs are the most advanced clinical delivery vehicles,^183–185^ currently showing strong potential for nucleic acids delivery in gene therapy.^186^ In addition to GalNAc-modified siRNAs, LNPs are the only small nucleic acid carriers approved by the FDA for the targeted delivery of siRNAs.^187^ The unique property of LNPs is their neutrally charged surface, which enables them to avoid extensive adsorption by serum proteins and prevent poor penetration into the target tissue.^188,189^ Unlike liposomes, LNPs contain multiple reverse micelle nuclei composed of nucleic acids and lipids, which are surrounded by a monolayer lipid membrane.^190^ Four primary lipids are used to form the shells of LNPs, namely, ionizable lipids, polyethylene glycol (PEG)-modified lipids, cholesterol and helper phospholipids, which are present in suitable proportions (commonly known as molar ratios of 50, 1.5, and 38.5/10, respectively).^191^ The composition of the LNP shell is quite important in the physicochemical properties and functions of LNPs, and the optimal ratio can vary under different conditions. Each lipid in the LNP shell plays a different but similarly essential role in effective small nucleic acid delivery. Ionizable lipids are the major component of LNPs because of their potency. Ionizable lipids can ensure that the surface of LNPs remains neutral at physiological pH, given their precise and appropriate pKa, which is between 6.2 and 6.5, different from previous cationic lipids.^192,193^ The ionizable lipids can respond to different pH values, remaining neutral at pH 7 but becoming positively charged at lower pH values.^193^ Therefore, for neutral LNPs, tolerability and safety are further increased by preventing reactions with negatively charged biomacromolecules, which can trigger severe side effects that are unavoidable when permanently charged cationic lipids are used.^194^ Moreover, a PEGylated lipid is a hydrophilic PEG polymer conjugated to a hydrophobic lipid anchor, with the lipid domain within the polymer structure and the PEGylated domain extending out from the polymer surface.^195^ PEG can prolong the half-life of LNPs in the circulation, since it can adsorb the serum proteins in blood.^196–198^ However, it can also reduce the proximity of LNPs to cell membranes and inhibit fusion between LNPs and cell membranes, leading to a decrease in nucleic acid uptake efficiency.^199^ Research has shown that a higher PEGylated lipid content results in a smaller particle size.^200^ However, the reduced delivery efficiency is driven primarily by an increase in the PEG content rather than by a decrease in the particle size.^201^ Thus, the optimal dose of PEGylated lipids should be selected to maintain a balance between stability and transfection competency and safety. Cholesterol maintains the stability of the LNP surface while increasing its delivery efficiency since it can fill the gaps between different lipids, reduce the possibility of immune clearance and bind Apo E and LDL receptors to aid in LNP uptake.^202,203^ The addition of cholesterol analogs and C-24 alkyl phytosterols not only increases the cellular uptake of nucleic acids but also prolongs the retention time after endosomal escape.^204^ Helper phospholipids, such as 1,2-dioleoyl-sn-glycero-3-phosphoethanolamine (DOPE) and 1,2-distearoyl-sn-glycero-3-phosphocholine (DSPC), are always zwitterionic, which can increase the stability and delivery efficiency of LNPs. Cone-shaped DOPE forms an inverted hexagonal (HII) phase, and cylinder-shaped DSPC can provide greater bilayer stability.^205^

The most commonly used technology for LNP synthesis is the ethanol dilution method, which uses a microfluidic device. Generally, by mixing a lipid solution dissolved in ethanol and a small nucleic acid solution dissolved in aqueous buffer (pH 4), positively charged ionizable lipids bind to negative nucleic acids and subsequently encapsulate RNAs to form LNPs with other lipids.^206^ The residual ethanol is then removed from the pH 4 buffer through dialysis, followed by increasing the pH (7.4) for long-term storage and subsequent use.^191^ The particle size of LNPs is associated with the speed of ethanol dilution with a buffer solution, where more rapid dilution produces smaller LNPs, suggesting that the desired microfluidic device should be able to undergo rapid and controllable mixing.^200,207^ Detection methods have also improved, thereby improving the quality control of LNPs. For example, Kim et al. developed a robust high-performance liquid chromatography with charged aerosol detector (HPLCCAD), which can quantify the composition of LNPs using the designed approach.^208^

Research has shown that LNPs enter cells through clathrin-mediated endocytosis and micropinocytosis.^209^ After internalization, the nucleic acid–LNP complex is transported through early endosomes, late endosomes, and lysosomes, all of which have pH values less than 7.^210^ Small nucleic acid release occurs mainly in a moderately acidic early endocytic compartment before transport to late endosomes and lysosomes.

However, several hurdles limit the application of LNPs, the majority of which are safe and associated with LNP formulations. Although PEGylated lipids attribute a lot to LNP delivery systems, they are also the causes of various adverse reactions induced by nucleic acid-LNP drugs.^211^ First, accumulating evidence indicates that PEG can induce hypersensitivity reactions by stimulating the complement system.^212,213^ Moreover, PEGylated lipids could lead to accelerated blood clearance (ABC) phenomenon, reducing their therapeutic efficacy.^214–217^ Furthermore, another disadvantage of PEGylated lipids is their nonbiodegradability, suggesting that PEGs should have a relatively low molar mass, generally less than 20 kDa.^218–220^ Additionally, the molar mass should be greater than 400 Da since a lower mass is toxic in humans.^221^ Many efforts have been made to reduce the side effects of LNP treatment. Judge et al. evaluated LNPs the stabilities of LNPs with different PEGylated lipids, results showed that PEGylated lipids with shorter alkyl chains (C14) are more stable.^222^ To date, new PEGylated lipids have been preferentially designed, which can disengage from the LNPs after injection via a process called PEG shedding, thus maintaining the advantages of PEGylated lipids and simultaneously avoiding their side effects.^223,224^ For example, Hatakeyama et al. developed PEG–peptide–DOPE (PPD), showed higher efficiency of cellular uptake and endosomal escape.^225^ The long-chain PEG 5000 polymer O’-methyl polyethylene glycol (omPEG), which is a pH-sensitive PEG shed from LNPs, has low toxicity to blood and noncancerous intestinal cells.^226^ In addition, the fate of the nucleic acid–LNP complex is also a noteworthy issue, as nucleic acid–LNP complexes accumulate in large amounts in the liver when they are administered intravenously.^227–229^ The target organs of LNPs can be changed by altering the lipids used to form the LNP shell. For example, DSPC-containing LNPs preferentially accumulate in the spleen, whereas DOPE-containing LNPs preferentially accumulate in the liver, suggesting that different helper lipids should be chosen for different uses of LNPs.^230^ Additionally, adding a target molecule into an LNP can affect its delivery endpoint. For example, Lee et al. incorporated a Glu-urea-Lys PSMA-targeting ligand into the LNP system, resulting in increased LNP accumulation at tumor sites.^231^ Ramishetti et al. constructed tLNPs that can specifically deliver nucleic acids to CD4( + ) T lymphocytes by chemically conjugating mAbs against CD4 to the LNPs. After intravenous injection, particles were accumulated in lymph nodes, blood, and bone marrow, and effectively endocytosed by CD4( + ) T lymphocytes.^232^ Younis et al. designed ultrasmall lipid nanoparticles with a novel pH-sensitive lipid and a targeting peptide, exhibiting excellent tumor accumulation and gene silencing in vivo.^233^ In addition, by adding polyelectrolyte layers to LNPs, poly-L-arginine and hyaluronan (HA) can cross the BBB, targeting glioblastoma multiforme (GBM) cells more effectively than unmodified LNPs in vitro and delaying tumor growth in vivo after siRNA and miRNA transfection.^234^ Altering the administration route might also be a viable option. Bai et al. developed an inhalable and mucus-penetrating LNP system that can significantly diminish fibrosis development.^235^ Wang et al. constructed lipidoid (lipid-like) nanoparticles to deliver a VEGF siRNA into the eyes through intravitreal administration and treat retinal angiogenic diseases.^236^ In summary, LNPs show great potential in small nucleic acid delivery.

#### Lipid nanoemulsions (LNEs)

LNEs are nanosized spherical particles composed of surfactants and phospholipids and are approximately 200 nm in size. The structure of LNEs not only is similar to that of liposomes but also remains biodegradable, biocompatible and nonimmunogenic.^237^ Three dispersions of LNEs are commonly used—oil in water (O/W), water in oil (W/O) and water in oil in water (W/O/W)—of which O/W is the most commonly used in nucleic acid drug delivery.^238^ The oil phase of LNEs can protect nucleic acids from degradation by nucleases. Although no proteins are components of LNEs, apolipoproteins, such as ApoA, ApoC and ApoE, can be incorporated into LNEs through contact with plasma in the blood circulation. ApoE can be recognized by the low-density lipoprotein (LDL) receptor, which is overexpressed in some tumors; thus, LNEs can deliver nucleic acids to tumor cells with high LDL receptor expression.^239^ Changing the proportions of components or directly changing the composition of LNEs could alter their physicochemical characteristics and in vivo biodistribution. By increasing the weight ratio of medium-chain triglycerides to soybean oil from 1:4 to 3:2, Jiang et al. reported that the LNE size decreased from 126.4 ± 8.7 nm to 44.5 ± 9.3 nm. The addition of PEGylation increased the accumulation of LNEs in the tumor area by 6–7-fold.^240^ Kaneda et al. formulated LNEs consisting of DOTAP, DOPE and cholesterol. These siRNA-LNE complexes can be internalized through lipid raft transport, bypassing the usual endosomal nanoparticle uptake pathway and increasing drug availability.^241^ Compared with liposomes, the manufacture of LNEs is less expensive. Gehrmann et al. investigated the preparation of LNEs with disposable materials. By changing different syringe filters and pharmaceutically relevant emulsifiers, they finally developed a stable protocol to quickly and easily produce LNEs with narrow particle size distributions.^242^ Unfortunately, LNEs are more commonly used in the delivery of poorly water-soluble, highly lipophilic drugs but not hydrophilic nucleic acid drugs, as their oil phase can solubilize these drugs.^243^

#### Solid lipid nanoparticles (SLNs)

SLNs are spherical nanoparticles composed of biodegradable physiological lipids and surfactants with a drug-containing solid lipid core, and thus they are less toxic than other nanodelivery systems.^244^ The melting points of the employed lipids, such as monostearin, stearyl alcohol, stearic acid, glycerol monostearate and Precirol® ATO5, are above room temperature.^245^ Unlike LNEs, organic solvents are not used in the preparation of SLNs, which may introduce potential toxicity.^246^ Interestingly, Shirvani et al. produced a novel type of SLN using natural beeswax and propolis wax, both of which are considered food grade.^247^ SLNs are stable in biological fluids and during storage for at least one week at room temperature and 1 month at 4 °C.^248^ Lyophilization can further improve the storage stability of the solid forms of SLNs and retard unfavorable chemical degradation.^249^ In addition, by coating the surface of SLNs with chitosan, the residence time in the focal area of the drug–SLN complex can be further improved via mucoadhesive interactions.^250^ The size of LNEs may influence their in vivo distribution. By comparing SLNs with different particle sizes (120–480 nm), Huang et al. reported that a larger particle size led to a longer lung retention time.^251^ In addition, SLNs are quite easy to sterilize and mass produce.^252,253^

However, some obstacles prevent SLNs from delivering nucleic acids. For example, during the solidification process, the inner loaded nucleic acids might be excluded from the surface of SLNs, which are surrounded by an aqueous phase, resulting in an insufficient loading efficiency.^254^ In addition, during storage, SLNs are prone to aggregate and gelatinize.^254^

#### Nanostructured lipid carriers (NLCs)

NLCs contain a lipophilic core formed by a mixture of one solid lipid (in a greater quantity) with one liquid lipid (in a lower quantity).^255^ NLCs are recognized as second-generation SLNs, which address their limitations. First, liquid lipids are added to the solid lipid matrix of SLNs to disrupt the perfect crystal order, thereby stabilizing the LNEs and preventing loaded nucleic acid leakage during storage.^256^ By adjusting the ratio of liquid lipids to solid lipids, NLCs can also maintain a solid skeleton structure at room temperature for a long period, which is conducive to the controlled release of loaded nucleic acids.^257^ In addition, the payload of NLCs is increased, and the bioavailability of loaded nucleic acids is further improved.^258^ Because of these alterations, NLCs are more suitable for carrying biologically active ingredients.^259^ However, relatively few studies have investigated the use of NLCs for small nucleic acid delivery. Oner et al. successfully prepared 13 NLCs with characteristics similar to those of their precursor SLNs, but not all of these NLCs could form complexes with siRNAs.^260^ More detailed information about the LBNP system mentioned in this section is listed in Table 1.

**Table 1:** LBNP-based nanoparticles for nucleic acid drug delivery

Composition	Size (nm)	Z potential (mV)	Disease	Nucleic acid drug	Therapeutic target	Administration route	RNA EE [%]	Ref.
YSK05/DOPE/Cholesterol/PEG-SP94/PEG = 5:2: 3:1: 0.3	60.47 ± 6.9	−17.4 ± 5	HCC	siRNA	Midkine	Intravenous injection	94.5 ± 6.5	^233^
DMAP-BLP/DSPC/Cholesterol/PEG-DSG = 40: 17.5: 40: 2.5 mol%	84.5 ± 32.5	-	Advanced PCa	siRNA	AR	Intravenous injection	-	^231^
MC3/DSPC/ Cholesterol /PEG2000-lipid= 50:10:38.5:1.5	89.74 ± 4.89	1.13 ± 0.51	-	siRNA	-	Intravenous injection	93.6 ± 3.8	^229^
PLGA: PEG: PAMAM dendrimer: 1,2-epoxytetradecane =1:1:1:5	130.9 ± 2.17	1.97 ± 0.10	Lung fibrosis	siRNA	IL11	Nebulization	93.12	^235^
synthesized lipid: Cholesterol: DOPE: DSPE-PEG2000 = 16:4:1:1	150	-31.7	-	ASO	PCSK9	Intravenous injection	97	^185^
DODM: Cholestero: DOPE = 50:39:11% mol	128 ± 1	13 ± 1	GBM	miRNA	miR-181a	Intertumoral injection	91 ± 1	^234^
Amine: Acrylates=1:2.4	212.5 ± 1.6	2.0 ± 0.3	Retinal neovascularization	siRNA	VEGF	Intravitreal injection	-	^236^
DLin-MC3-DMA: DSPC: Cholesterol: DMG-PEG2000 = 50:10:38.5:1.5	60 ± 10	-	AMLs	siRNA	RUNX1/ETO	Intraperitoneal injection	-	^198^

*LBNP* Lipid-based nanoparticle, *EE* encapsulation efficiency, *HCC* hepatocellular carcinoma, *PCa* prostate cancer, *GBM* glioblastoma, *AD* Alzheimer’s disease, *AMLs* acute myeloid leukaemia

### Peptides

Cell-penetrating peptides (CPPs) and homing peptides consist of 5–30 amino acids and interact with biomolecules and cells to increase nucleic acid delivery efficiency.^261^ Compared with viral carriers, peptides of varying sizes and structural features have the advantages of a greater payload capacity and biocompatibility and have broad applications.^262^ Moreover, rationally designed peptides can overcome a series of biological obstacles, such as endosomal escape, cell internalization, and interactions with biomolecules and cells, to improve nucleic acid delivery efficiency. The side chains of these materials can carry various active functional groups that can be chemically modified to enhance the drug delivery systems. In addition, peptides can also be integrated as functional fragments into drug delivery systems.

CPPs and intracellular targeting peptides are of particular interest because they can improve the delivery efficiency of gene therapies into cells. CPP also known as a protein transduction domain (PTD), is a kind of membrane-active peptide that typically contains 5–30 amino acids. CPPs were initially designed to mimic the transactivator of transcription (TAT) protein transduction domain,^263^ penetratin,^263^ and Pvec,^263^ which can cross cellular membranes via energy-dependent or -independent mechanisms without interacting with specific receptors.^264^ Nucleic acids are attached to CPPs via covalent bonds or noncovalent complex formation.^265^ Noncovalent complex formation depends on electrostatic and/or hydrophobic interactions between negatively charged cargoes and positively charged CPPs, protecting bioactive conjugates from proteases or nucleases.^265,266^ Moreover, peptides can covalently interact with nucleic acids to produce conjugated molecules, and this method is suitable for generating neutral nucleic acids.^267^ Additionally, functional peptide fragments can be coupled to nucleic acids for delivery through chemical bonds (such as ester bonds, disulfide bonds, and thiol maleimide bonds). Peptide‒nucleic acid coupling molecules have specific structures, molecular weights, and high stability, with an excellent degree of reproducibility.^268^ CPPs are classified into three major categories according to their physicochemical characteristics: cationic, amphiphilic, and hydrophobic. Numerous preclinical evaluations of CPP-derived therapeutics have been performed to address major unmet medical needs (Table 2).

**Table 2:** Representative cell-penetrating peptides

Class	CPP name	Sequence	Origin	Ref.
Cationic	TAT (48-60)	GRKKRRQRRRPPQ	HIV-1 TAT	^263^
	TAT (49-57)	RKKRRQRRR	HIV-1 TAT	^263^
	Penetratin, pAntp (43-58)	RQIKIWFQNRRMKWKK	Antennapedia Drosophila melanogaster	^263^
	Polyarginines	Rn	Chemically synthesized positively charged sequence	^279^
	DPV1047	VKRGLKLRHVRPRVTRMDV	Chemically synthesized positively charged sequence	^272^
	DP7	VQWRIRVAVIRK	Parent peptide HH2	^762,763^
Amphipathic	MPG	GALFLGFLGAAGSTMGA	A fusion sequence of HIV gp41 and NLS of SV40 T-antigen	^281^
	Bac 7 (Bac _1-24_)	RRIRPRPPRLPRPRPRPLPFPRPG	Bactenecin family of antimicrobial peptides	^298^
	pVEC	LLIILRRRIRKQAHAHSK	Vascular endothelial cadherin	^288^
	ARF (1-22)	MVRRFLVTLRIRRACGPPRVRV	the tumor suppressor protein p14ARF	^289^
	CADY	GLWRALWRLLRSLWRLLWRA	Derived from PPTG1 peptide, W and charged amino acids	^291,295^
	M918	MVTVLFRRLRIRRACGPPRVRV	the tumor suppressor protein p14ARF	^289^
	BPrPr (1-28)	MVKSKIGSWILVLFVAMWSDVGLCKKRP	N terminus of unprocessed bovine prion protein	^290^
	Pep-1	KETWWETWWTEWSQPKKKRKV	NLS from Simian Virus 40 large T antigen and reverse transcriptase of HIV-1	^271^
	MAP	KLALKLALKALKAALKLA	Chemically synthesized amphipathic model peptide	^292^
	VT5	DPKGDPKGVTVTVTVTVTGKGDPKPD	Chemically synthesized	^764^
	Transportan	GWTLNSAGYLLGKINLKALAALAKKIL	Galanin-Lys-mastoparan	^285^
	p28	LSTAADMQGVVTDGMASGLDKDYLKPDD	Azurin	^286^
Hydrophobic	C105Y	CSIPPEVKFNKPFVYLI	α1-Antitrypsin	^305,306^
	PFV	PFVYLI	C105Y	^307^
	Pep-7	SDLWEMMMVSLACQY	CHL8 peptide phage clone	^305,306^

*HIV* Human Immunodeficiency Virus

Cationic CPPs are typically short (up to 30 amino acids) and rich in arginine and lysine residues that have positive net charges.^269^ The positive charge of cationic peptides is attracted to negatively charged membrane constituents, enhancing the interaction of CPPs with the cell membrane to initiate transport.^270^ The TAT protein transduction domain was first identified in the HIV genome and is the first and most studied type of CPP. After the discovery of the TAT peptide, additional CPPs, such as Pep-1, polyarginine, and penetratin, were discovered.^271,272^ The structural prerequisites for the cellular uptake of cationic CPPs have been widely examined in various studies. For example, after cholesterol modification, the DP7 peptide (DP7-C) has shown significant potential because of its unique properties and effectiveness in facilitating cellular uptake.^273–277^ It can be conjugated with various nucleic acids, including siRNAs and miRNAs, enhancing the stability and bioavailability of nucleic acids.^275–277^ This broad applicability allows DP7-C to be used in a variety of therapeutic settings, treating numerous diseases and conditions.^275–277^ However, the precise mechanisms by which cationic CPPs enter the cell membrane are complex and debated. Arginine-rich CPPs are taken up by lipid raft-dependent macropinocytosis, independent of caveolar, clathrin-mediated endocytosis, and phagocytosis.^278^ Other studies suggest they may directly cross the membrane via transient pores.^279^ Recently, arginine-rich CPPs were shown to go through vesicles and live cells via vesicular fusion induced by calcium influx, similar to the mechanism of membrane multilinearity and fusion.

Peptides with both polar and nonpolar regions are known as amphipathic CPPs, typically classified as primary, secondary, or proline-rich peptides.^280^ Primary amphipathic CPPs are chimeric peptides formed by covalently attaching a hydrophobic domain to a nuclear localization signal (NLS), enhancing their ability to target cell membranes effectively. The peptide carrier MPG, derived from the fusion peptide domain of the HIV-1 gp41 protein combined with the nuclear localization sequence of the SV40 large T antigen, efficiently delivers siRNAs into mammalian cells.^281^ Furthermore, a refined and shorter variant of the amphipathic peptide carrier MPG, called MPG-8 (AFLGWLGAWGTMGWSPKKKRK), enhances the efficiency of siRNA delivery both in vitro and in vivo, without triggering an immune response.^282^ In a mouse xenograft tumor model, stable and noncovalent nanoparticles were generated with a cyclin B1 siRNA to block tumor cell proliferation and tumor growth.^282^ Additionally, peptide transduction domain–dsRNA binding domain (PTD-DRBD) fusion proteins efficiently deliver siRNAs.^283^ In vivo delivery of EGFR and Akt2 siRNAs via PTD-DRBD triggered apoptosis specifically in tumor cells and prolonged survival in mice bearing glioblastoma.^284^ Moreover, secondary amphipathic α-helical CPPs feature a highly hydrophobic patch on one side, whereas the other side can be cationic, anionic, or polar.^285,286^ In an interesting study, the siRNA delivery efficiencies of cationic and amphipathic CPPs were compared, and the results revealed that the amphipathic CPPs are more suitable as carrier moieties for delivering siRNA polyplexes.^287^ In addition to MPG and Pep-1, other known primary amphipathic CPPs are derived from natural proteins. These CPPs include an 18-amino acid-long peptide originating from vascular endothelial cadherin (pVEC),^288^ a 22-amino acid-long peptide derived from the N-terminal region of p14ARF, named ARF (1–22),^289^ and a peptide sourced from the N-terminus of the unprocessed bovine prion protein (BPrPr).^290^ Secondary amphipathic CPPs typically display a unique structure, adopting an α-helical conformation wherein hydrophilic and hydrophobic amino acids are segregated onto separate faces of the helix.^291^ The use of secondary amphipathic CPP-based delivery systems provides therapeutic carriers with high delivery efficiency and no cytotoxicity or immunogenicity. Model amphipathic peptide (MAP) is a well-studied CPP featuring an α-helical structure with hydrophilic and hydrophobic residues arranged on opposite sides of the helix.^292^ Comprising a sequence of alanines, leucines, and lysines (KLALKLALKALKAALKLA),^292^ MAP has been shown to be an effective carrier for delivering siRNA into cells.^293,294^ CADY is a 20-residue peptide that adopts a helical conformation within cell membranes and forms stable complexes with siRNAs.^291,295^ Another type of amphipathic CPPs contains proline-rich CPPs. Although these CPPs vary in sequence and structure across different families, they all feature a proline pyrrolidine template.^296–298^ Apidaecin and oncocin belong to this group of peptides and can permeate the BBB in mice.^299,300^ PR39 is a proline- and arginine-rich peptide implicated in wound healing and protection against myocardial ischemia.^301^ PR39 has been utilized as an innovative carrier for delivering siRNAs into the cell cytoplasm, and loading a Stat3 siRNA has shown synergistic effects in suppressing the invasion and migration of 4T1 cells.^302^

Hydrophobic CPPs with a low positive or negative net charge tend to have poor solubility and easily aggregate.^303^ Hydrophobic CPPs have recently received increasing amounts of attention due to the extensive presence of positive charges that cause cationic CPPs to be toxic.^304^ For example, natural C105Y, its C-terminal portion (PFVYLI), and Pep-7 are part of this group, and the affinity of their nonpolar amino acids for the hydrophobic domain of cell membranes aids in their translocation.^305–307^ Several modified peptides can also be generated using various approaches.^308^ The chemically modified hydrophobic CPP TP10 has four cationic amino acid (Lys) residues among the many hydrophobic amino acid residues, enabling efficient delivery of a splice-correcting 2’-OMe RNA oligonucleotide.^309^ Therefore, hydrophobic CPPs hold significant potential for the noncovalent delivery of negatively charged oligonucleotides.

Although the groundbreaking and versatile peptide tools have received heightened interest in their application within gene therapy, peptide nanocarriers must be tested in clinical trials before they can be applied in clinical settings. Therefore, additional basic science and preclinical studies are needed to accelerate peptide-based vector development.

### Polymers

Since the first human trial approval (NCT01644890) was obtained in the early 1990s, polymeric drugs have entered the market as potent therapeutics.^310,311^ Innovative polymer-based drug delivery systems have gained significant interest in gene therapy. The polymers employed for gene delivery differ greatly in terms of molecular weight, structure, and composition.^312^ With linear, branched or dendritic structures, these polymers electrostatically bind negatively charged nucleic acids through their inherent proton sponge behavior, offering protection and promoting cellular uptake.^312–314^ Further chemical modifications of polymers optimize the transfection efficiency, biocompatibility, cell selectivity, and in vivo distribution and reduce cytotoxicity.^313,315^ In general, synthetic and natural polymers are completely distinct. Polymers derived from natural sources, such as plants, animals, and microorganisms, have the advantages of biocompatibility, mechanical properties, biodegradability and renewability, whereas synthetic polymers are easier to generate and modify and have been widely utilized in all applications due to their structural and mechanical properties; however, they are unable to perform certain biological functions.^316–318^ One of the first widely utilized polymers in gene delivery was polyethyleneimine (PEI), has shown great potential for transfecting dividing cells.^319^ Regrettably, its cytotoxicity has restricted its application in vivo and in clinical trials, as it has been found to cause membrane damage and initiate apoptosis in clinically relevant human cell lines.^320^ Currently, a range of polymers, such as poly(b-amino esters), poly(amido ethylenimines), and dendrimers, have emerged,^321^ demonstrating high transfection efficiency and low cytotoxicity (Table 3).^322–328^

**Table 3:** Polymer-based nanoparticles for nucleic acid drug delivery

Composition	Particle size (nm)	Disease	Nucleic acid drug	Therapeutic target	Administration route	Ref.
Glu-PEG-PLL, MeO-PEG-PLL	45	CNS disorders	ASO	-	Intravenous injection	^322^
Porous poly(γ-butyrolactam), poly(ε-caprolactam),	-	*C. albicans*	2’- O MethylRNA ASO	EFG1	-	^323^
PAMA, C7A-MA, DBA-MA, PEG	30	Retinal neovascularization	siRNA	VEGFA	Intravitreal injection	^324^
Branched PEI, Linoleoyl chloride, Lauroyl chloride	175 ~ 227	Myeloid leukemia	siRNA	FLT3	Intravenous injection	^325^
DSPE-PEG_2000_, QACs; degradable polymer	70	Cancer	siRNA	PD-L1	Intravenous injection	^326^
DOTAP/ ionizable DLin-MC3-DMA, mPEG_5k_-b-PLGA_11.1k_	∼100	Cancer	siRNA	CD47, PD-L1	Intravenous injection	^327^
FeCl_3_·6H2O, PEI, HA, NH_4_OAc	227.0 ± 8.2	Melanoma	miRNA	E2F7	intratumoral injection	^328^

*CNS* Central Nervous System, *ASO* Antisense oligonucleotides, *PAMA* poly (aminoethyl methacrylate), *C7A-MA* 2-(hexamethylenediamine) ethyl methacrylate, *DBA-MA* 2-(dibutyl amino) ethyl methacrylate, *PEI* polyethyleneimine, *QACs* quaternary ammonium compounds, *PIBCA* poly(isobutylcyanoacrylate), *DEAE* diethylaminoethyl

#### Poly(L-lysine) (PLL)

Poly-L-lysine (PLL), synthesized from L-lysine found in high-protein foods like meat and eggs, has inherent properties of non-toxicity, non-antigenicity, biocompatibility, and biodegradability.^329,330^ As a cationic polymer, PLL can be protonated in physiological conditions, allowing it to electrostatically bind with negatively charged nucleic acids and form complexes.^331^ Although PLLs are biodegradable, their high degree of cationic toxicity limits their use in nucleic acid drug delivery.^322,332^ Chemical modifications, such as the introduction of polyethylene glycol (PEG), have been implemented to reduce the toxicity and increase the transfection efficiency of PLLs and thus improve their gene delivery performance. For instance, Kazunori Kataoka’s group developed a PEG-block-poly(L-lysine) (PEG-b-PLL) that features lysine amines modified with 2-iminothiolane (2IT) and the cyclo-Arg-Gly-Asp (cRGD) peptide at the PEG terminus.^333^ This modification of PEG-b-PLL resulted in better control over micelle formation and enhanced siRNA stability in the bloodstream; subsequently, the incorporation of siRNA into these micelle structures increased cellular uptake, improved the gene silencing efficacy of the siRNAs and increased drug accumulation in both the tumor tissue and tumor-associated blood vessels following intravenous injection.^333^ This successful application of the PLL modification potentially expands the utility of polymer-based siRNA therapies for cancer treatments that require intravenous injection.^333^ Dendritic PLL, which allows for the control of molecular size and shape, can also be utilized for gene delivery.^334^ The 6th generation of dendritic poly(L-lysine) (KG6), with 128 amine groups on its surface, was demonstrated to be an efficient siRNA carrier with minimal cytotoxicity.^335^ Another star-shaped copolymer consisting of a β-cyclodextrin core and PLL dendron arms was used for docetaxel and MMP-9 siRNA codelivery.^336^ A biomimetic nanocomplex consisting of a cationic nanocore formed by a membrane-penetrating helical polypeptide (P-Ben) and Sav1 siRNA, a charge-reversal intermediate layer of PLL-cis-aconitic acid (PC), and an outer shell of a hybrid membrane, was administered intravenously. This nanodrug effectively accumulated in the ischemia‒reperfusion-injured myocardium.^337^ Although polymeric materials are increasingly used in the development of second-generation polymer therapeutics, the applications of PLLs are still relatively restricted compared to other polycations like chitosan (CS), poly(ethyleneimine) (PEI), and poly(amido amine) (PAMAM) and further investigations should explore the development of an appropriate regulatory framework that can be universally applied in research.

#### PEI

PEI is a synthetic polymer introduced in 1995, featuring an amine group and two CH2 spacers making it a cationic polymer suitable for nucleic acid delivery..^338,339^ PEI has two distinct chemical structures, namely, branched PEI (BPEI) and linear PEI (LPEI), with molecular weights ranging from 1 to 1000 kDa.^340,341^ BPEIs contain all types of various primary, secondary and tertiary amines, whereas LPEIs contain only secondary and primary amino groups.^341^ Interestingly, the capacity of PEI for gene delivery and cytotoxicity are greatly affected by its molecular weight. Therefore, it is important to carefully adjust the PEI structure to balance transfection efficiency and toxicity.^342^ Qiao et al. developed a successful PEI-siRNA delivery system, the dual siRNA-PEI (siRP) complex locally knocked down the target gene.^343^ Additionally, SNS01-T is a PEI-based formulation containing two nucleic acids: an siRNA and a plasmid. It has been shown to inhibit tumor growth in various animal models of B-cell cancers and demonstrates acceptable tolerability.^344,345^ In a Phase I/II clinical trial (NCT01435720), SNS01-T was administered intravenously in multiple doses and dose escalation stages to evaluate the safety and tolerability of the vector in patients with recurrent and refractory B-cell lymphoma. Although the trial was activated in 2011 and was completed in 2014, the results have not yet been published. Only one PEI-based small nucleic acid drug has been tested in clinical trials, highlighting the significant potential of novel PEI-based formulations to overcome current challenges related to drug stability.

#### Poly(β-amino esters) (PBAEs)

Poly(β-amino esters) (PBAEs) are a promising group of cationic polymers made from acrylates and amines, extensively developed for drug delivery.^346,347^ PBAEs are pH sensitive and protonated in acidic environments; they can be soluble at acidic pH levels but insoluble at physiological pH levels.^348^ PBAE-based drug delivery systems primarily enter cells by energy-dependent endocytosis and are split into separate vesicles by lipid bilayers.^349^ These separate vesicles are internalized into endosomes (pH<5.5), which subsequently transform into lysosomes (pH<4.5).^350,351^ Various PBAEs, synthesized from different diacrylate and amine combinations, offer diverse physicochemical and mechanical properties for drug delivery.^346,352^ In recent years, PBAEs have been extensively studied for convenient synthesis, low cost and good biocompatibility, and they can effective at activating antigens and boosting immune responses.^353–356^ Bioreducible PBAEs self-assemble with siRNAs in aqueous conditions to form nanoparticles and the ability of simple polymeric nanoparticles to efficiently knock down genes in primary human glioblastoma cells was greater than that of Lipo2000.^357^ Additionally, synthetic end-modified poly(beta-amino ester) (PBAE)-based nanoparticles can improve siRNA delivery into human mesenchymal stem cells (hMSCs).^358^ Although PBAEs were not approved for clinical use following initial human studies, their significant benefits make them promising candidates for medical applications.

#### Dendrimers

Dendrimers are highly branched, monodisperse, tree-like macromolecules.^359^ Unlike hyperbranched polymers, dendrimers exhibit random branching and have well-defined and regular nanoarchitectures with spherical shapes.^360,361^ Moreover, the surface of dendrimers can be chemically modified in multiple ways to alter the functionality of the macromolecules, and the branched nature of dendrimers results in a very high surface-to-volume ratio.^362^ A relatively empty dendrimer matrix is amenable to host molecule entrapment, facilitating precise, controlled payload release. Dendrimers are prepared via divergent or convergent synthesis.^363^ The divergent synthesis method has the advantage of modifying the dendrimer molecules starting from the core, whereas the convergent method allows greater control of the modification of molecules at specific positions responsible for specific functions.^364,365^ Commercially available poly(amidoamine) (PAMAM; Starburstk) dendrimers and poly(propylenemine) (also called PPI, DAB; AstramolR) dendrimers have been most widely studied and applied in drug delivery.^366,367^ Therapeutic drugs can be encapsulated within the core of PAMAM or conjugated to their surface, facilitating delivery to target cells. The development of covalently bonded hydroxyl-terminated PAMAM dendrimer–siRNA conjugates allows for precise nucleic acid loading, which has been observed using a GFP-targeted siRNA (siGFP) conjugate (D-siGFP).^368^ Additionally, the conjugation of PAMAM to the thermosome acts as an anchor for siRNAs, and further modification of the protein cage with a CPP increases the delivery efficiency of the siRNA transfection reagents.^369^ However, the synthesis of dendritic architectures through convergent or divergent methods may disrupt the design and construction, resulting in increased difficulty in mass production. A novel PEG modification strategy that preserves the surface amino groups of polymers has been proposed.^370^ Catechol-PEG polymers were used to modify the surface of PBA-modified generation 5 (G5) PAMAM dendrimers (G5PBA) via reversible boronate esters.^370^ This approach maintains the free amines of G5PBA, aiding in siRNA loading, stable complex formation, and increased transfection efficacy in serum.^370^ PEG-modified dendrimer/siRNA polyplexes show a similar gene silencing efficacy to non-PEG-modified polyplexes under serum-free conditions but superior performance in serum due to PEG shielding.^370^ In vivo, PEG- and RGD-modified dendrimer/siRNA nanoassemblies target tumors effectively, with PEG dissociating in acidic environments, allowing efficient gene silencing by G5PBA/siRNA polyplexes.^370^ Generally, the activation of functional groups on both PAMAM and the thermosome facilitates conjugation, typically using a crosslinking agent such as EDC/NHS (1-ethyl-3-(3-dimethylaminopropyl)carbodiimide/N-hydroxysuccinimide) to form stable amide bonds.^371^ Once the PAMAM dendrimers are conjugated to the thermosome, the positively charged surface amine groups of PAMAM can electrostatically interact with the negatively charged phosphate backbone of the siRNAs. This strong electrostatic interaction allows siRNA molecules to bind efficiently to the surface of the modified thermosome.^369,368^ One of the most common methods for attaching CPPs to the protein cage involves covalent bonding^372^ This process is typically achieved through reactive side chains on the amino acids of the protein cage. For example, lysine residues, which contain primary amine groups, can be targeted for conjugation with CPPs that have been preactivated with functional groups such as NHS esters or maleimides.^373^

Click chemistry is an efficient method for dendronizing β-cyclodextrin macrocycles, and the emergence of click chemistry has led to the generation of a dendritic delivery system with good yield and increased uniformity.^374,375^ This approach facilitates the rapid and reliable formation of covalent bonds between molecular components, resulting in the synthesis of dendritic systems with excellent yields. Moreover, click chemistry reduces the likelihood of side reactions, leading to increased uniformity of the final product.^376^ Copper-assisted azide‒alkyne cycloadditions (CuAAC), thiol‒ene click (TEC) reactions and Diels–Alder (DA) reactions are commonly applied to dendrimers. Luis José López-Méndez et al. introduced a novel dendritic material created by combining β-cyclodextrin (βCD) with second-generation poly(ester) dendrons.^374^ The dendrons were selectively attached to the seven positions on the primary face of βCD via a CuAAC click reaction. This method, along with a straightforward work-up process, enables the production of monodisperse materials with exceptionally high yields.^374^ Several hundred articles were surveyed and analyzed in this field. Due to the length of this review, we have not given special attention to the click chemistry applied to dendrimer synthesis.

### Exosomes

Exosomes (also called extracellular vesicles) are nanosized vesicles, ranging from 30 nm to 150 nm, composed of various proteins and RNAs surrounded by a lipid bilayer membrane, similar to liposomes in structure.^377^ The concept of exosomes was first proposed in 1981; Trams et al. reported that exfoliated membrane vesicles with 5’-nucleotidase activity are present in the culture supernatants of some cell lines and these vesicles may have a physiological function.^378^ Natural exosomes are derived from the endocytosis of plasma membrane.^379,380^ First, invagination of the cytoplasmic membrane is processed to form the early endosomes (ESEs). Then, by exchanging materials with other organelles, the early endosomes form late endosomes (LSEs) and further sprout into multivesicular bodies (MVBs). After MVBs fusing with cell membrane, exosomes are finally bud.^381^ Since exosomes are derived from natural cells, they not only are highly biocompatible but also have low immunogenicity, thus realizing high transfection (in vitro) and delivery (in vivo) efficiencies without inducing serious adverse effects.^382^ Once considered a trivial biological phenomenon, exosomes have recently garnered much attention for use as nucleic acid delivery systems.^383^ Rosas, L. E. et al. added of HEK293T-derived exosomes into human monocytic cell lines, results showed that no potential cytotoxic effects were observed.^384^ Furthermore, a series of studies revealed that no severe immune reactions were observed in mice or humans following repeated administration of exosomes either from mice or humans.^385–387^ In addition, exosomes possess a unique homing effect in which they preferentially target the parent cell from which the exosomes are produced.^388^

There are two major types for nucleic acids loading, including active loading, which includes mechanical extrusion,^389^ sonication,^390^ electroporation^391–396^ and repeated freeze‒thaw cycles,^397^ and passive loading, which includes direct incubation^398^ and exosome transfection.^399,400^ Shan et al. used exosome membranes obtained from repeated freeze‒thaw cycles to encapsulate DEP-siRNAs by coextrusion with a liposome extruder (220 µm, 12 times), thus generating a stable EM@DEP-siRNA complex.^401^ The mixture consisting of siRNAs and exosomes was ultrasonicated at an ultrasonic power of 25 W for 6 cycles. After incubated for 30 min at 4 °C, Angiopep-2 was added for a 24 h incubation. Finally, the unconjugated An2 and unloaded siRNA were removed to obtain Exo-An2-siRNA.^390^ Electroporation is the most established method used for this purpose; however, the conditions may vary among protocols, and numerous methods can achieve efficient loading to modulate target gene expression.^402–406^ The direct incubation of exosomes and siRNAs is time consuming with low efficiency. Tian et al. modified the siRNAs with 2′Ome through the conjugation of cholesterol at the 3′-terminus, thus increased the siRNAs loading efficiency. The loading efficiency of cholesterol-modified siRNAs in exosomes was highly increased and nearly 3-fold greater than that resulting from electroporation.^398^ Moreover, Exo-Fect is commonly used as a cell transfection reagent. After the exosomes were mixed with Exo-Fect and the desired nucleic acid cargo and incubated for an hour, the complex was added to the recipient cells. The Exo-Fect-induced loading procedure is time-saving and easy and increases the cellular uptake of the nucleic acid cargo.^407^ Nucleic acids loaded exosomes can be endocytosed by cells through different uptake pathways, including clathrin-coated pit-mediated internalization and micropinocytosis.^408^ The exosomal uptake efficiency of phagocytic cells is much greater than that of nonphagocytic cells.^409^ These internalization pathways can be divided into two main types: direct membrane fusion, which is the most likely route, and receptor-mediated endocytosis.^399^

However, the development of exosome delivery systems faces many difficulties. First, natural exosomes can migrate everywhere in vivo and do not have specific organ-targeting abilities, as verified by multiple experiments.^410^ Therefore, engineered exosomes are conducted to enhance their specific targeting of cells and organs. The most commonly used strategy involves modifying specific ligands or receptors on the membrane of exosomes. Through genetic engineering technology, homing proteins or polypeptides that effectively target recipient cells can be expressed on the membrane of exosomes. Generally, the plasmids are inserted with targeting peptide-fused exosomal membrane proteins, such as CD63, CD81, and Lamp2.^411^ For example, Liang et al. fused Her2, involved in tumor progression, to the exosomal surface to generate exosomes with the ability to target tumor cells, and the results revealed efficient accumulation and therapeutic effects both in vitro and in vivo.^412^ Additionally, Li et al. constructed peptide-fused Exos that contained CP05 and titanium-binding peptide (TBP), thereby achieving an exosome-targeted titanium implant.^413^ On the other hand, chemical modifications can directly conjugate target proteins or peptides to the membrane of exosomes. For example, Gunassekaran et al. produced IL4R-Exos (si/mi), the surface of which was modified with IL4RPep-1, and the accumulation of these exosomes was greater in tumors than in the liver.^414^ Ran et al. inserted neuron-targeting RVG peptides into the membrane with His-CP05 to form AP-ExoRs, and ex vivo imaging revealed a significantly increased fluorescence intensity.^415^ Chemical modification after exosome extraction is easier and more convenient than chemical modification prior to extraction, and biological engineering can protect exosomes without introducing other unwanted chemicals. In addition, selective organ targeting can also be achieved through physical engineering. For example, vesicle shuttles, consisted of an Fe3O4 core, a silica shell and anti-CD63 antibodies (exosome capture), can lead to a local release of the captured exosomes via magnetic fields.^416^ However, large-scale exosome production for clinical application is still difficult and expensive.^417^ In addition to stimulating exosome release through processes such as phototherapy-based light induction,^418^ the upregulation of certain genes (STEAP3, syndecan-4 and a fragment of L-aspartate oxidase),^419^ the introduction of electrical pulses provided by a cellular nanoporation biochip,^420^ and the production of bioinspired exosome-mimetic nanovesicles (NVs) have greatly increased the yield. NVs are produced by the breakdown of cells through serial extrusion using several filters of different decreasing sizes (10, 5, and 1 μm), similar to the natural production of exosomes.^421^ Furthermore, exosomes derived from tumor cells, especially tumor cells with an amplified oncogene, may transfer this oncogene along with nucleic acid drugs to target cells, further stimulating tumor growth since additional tumor-specific genetic properties, such as oncogenic DNA (c-Myc) and retrotransposon elements (HERV, L1 and Alu sequences), are present in these exosomes.^422^ However, in addition to immortalized cells, all nucleated cells present some level of risk for horizontal gene transfer (HGT). Usman et al. proposed generating exosomes from mature human red blood cells, which lack both nuclear and mitochondrial DNA, to resolve this issue, thus completely abrogating the risk of gene transfer.^423^ More detailed information about the exosome systems mentioned in this section is listed in Table 4.

**Table 4:** Exosome-based nanoparticles for nucleic acid drug delivery

Origin	Disease	Nucleic acid drug	Therapeutic target	Drug loading method	Administration route	Ref.
HEK293 cell	GBM	ASO	anti-miR-21	Electroporation	Intravenous injection	^391^
hMSCs	PD	ASO	α-syn	Electroporation	Intracerebroventricular injection	^392^
ReN cells	GBM	siRNA	PD-L1	Hydrophobic interaction	Intravenous injection	^398^
HEK293 cell	ZIKV infection	siRNA	ZIKV	Electroporation	Intravenous injection	^393^
Primary dendritic cells	PD	shRNA/siRNA	SNCA	Electroporation	Intravenous injection	^394^
Milk	Inflammatory bowel disease	siRNA	TNF-α	Electroporation	Oral	^395^
Granulocytic MDSCs	Colitis-associated cancer	miRNA	STAT3	Transfection	Intraperitoneal injection	^400^
BMSCs	SAH	miRNA	HDAC3	Electroporation	Peripheral nerve injections	^396^
Autologous breast cancer cells	Breast cancer	siRNA	S100A4	Freeze‒thaw cycles	Intravenous injection	^397^

*GBM* glioblastoma, *ASO* Antisense oligonucleotides, *PD* Parkinson’s disease, *ReN* ReNcell VM, *ZIKV* Zika virus, *hMSCs* human bone marrow mesenchymal stem cells, *MDSCs* myeloid-derived suppressor cells, *BMSCs* bone marrow mesenchymal stem cells, *SAH* subarachnoid hemorrhage

### Conjugates

A drug conjugate is an integrated drug delivery system comprising therapeutic agents and one or more carriers through a biodegradable linker.^424,425^ The conjugate that has received the most clinical attention is a trivalent carbohydrate ligand based on N-acetylgalactosamine (GalNAc), which has been approved by the FDA.^426–428^ The strategy of GalNAc-based conjugation relies on the specific recognition and combination of GalNAc with the asialoglycoprotein receptor (ASGP-R), located on the basolateral membrane of hepatocytes with a very high density.^429^ ASGP-R was first isolated in 1974. Both Hudgin et al. and Stockert et al. reported that ASGP-R is a galactose-binding protein,^430,431^ whereas Baenziger et al. reported ASGP-R prefers GalNAc, compared with galactose.^432^ ASGP-R contains four functional domains, namely, the cytosolic domain, transmembrane domain, stalk, and carbohydrate recognition domain (CRD), which can bind to the GalNAc ligand.^433^ Moreover, in a previous study, the sialic acid residue of ASGP-R was removed to expose the sugar residues and improve the binding affinity to GalNAc.^434^ Classical GalNAc-based nucleic acid drugs comprise three components: a triantennary GalNAc, a linker and a nucleic acid molecule.^435^ GalNAc is a sugar derivative obtained by acetylation after the C-2 hydroxyl group on galactose is replaced by an amino group. Lee et al. demonstrated that the inhibitory potency of the tetraantennary molecule was greater than that of the triantennary molecule, whereas that of the triantennary molecule was much greater than that of both the biantennary and monoantennary molecules (tetraantennary > triantennary >> biantennary >> monoantennary).^436^ Furthermore, the spacers between the GalNAc moieties and the branching point of the dendrite can also change the affinity for ASGP-R. The most potent sugar spacing was 20 Å (20 Å >10 Å >4 Å).^437^ Another investigation suggested that the cargo size of GalNAc-based drugs should be less than 70 nm for proper receptor recognition and efficient endocytosis.^438^

After GalNAc-conjugated drugs bind to ASGP-Rs, the complex is endocytosed through clathrin-mediated pathway.^439^ The GalNAc linker is then quickly removed before the nucleic acid escapes into the cytoplasm.^440^ Moreover, ASGP-R is recycled to the cell surface quickly (less than 15 min) for another internalization of nucleic acid cargo.^441^ Many studies have verified that attachment of the GalNAc moiety can greatly increase the efficiency of hepatocyte-targeted delivery by approximately 6–7-fold.^442,443^ Prakash et al. reported that unconjugated ASOs are predominantly ( > 70%) taken up by nonparenchymal cells in the liver, while GalNAc-conjugated ASOs prefer hepatocyte fraction in the liver ( > 80%).^444^ Sirnaomics designed an upgraded GalNAc platform named peptide docking vehicle-GalNAc (PDoV-GalNAc) which provides two binding sites for siRNAs and exhibits excellent property in endosomal escape. Thus, the PDoV-GalNAc can both accelerate the speed at which the drug cargo is delivered and increase the efficiency of target gene knockdown.

Other types of conjugates include cholesterol, tocopherol, vitamin E and fatty acids. Wolfrum et al. constructed cholesterol-conjugated siRNAs and performed in vivo experiments showing that they can silence gene expression, whose endocytosis is associated with some lipoprotein particles.^445^ Moreover, through covalent binding of alpha-tocopherol (vitamin E) to the 5’ end of antisense strand, the endogenous mRNA target in the liver can be effectively knocked down by conjugated siRNAs.^446^

Since ASGP-R is highly expressed on parenchymal hepatocytes and minimally expressed on other cells, GalNAc conjugates can be constructed to target liver lesions. Scharner et al. ectopically expressed ASGP-R in nonhepatic cancer cell lines to achieve extrahepatic delivery, results showed increased internalization efficiency of GalNAc-conjugated ASOs in vitro and greater splicing modulation than that mediated by unconjugated ASOs in vivo.^447^ In addition, lipid conjugation can cause small nucleic acids to accumulate in extrahepatic tissues and subsequently leads to gene silencing, providing an opportunity to realize extrahepatic delivery of therapeutic small nucleic acids. By systematically comparing the body distributions of a set of fatty acid-conjugated siRNAs, Biscans et al. reported that trivalent lipid-conjugated siRNAs mainly resided at the injection site, whereas monovalent lipid-conjugated siRNAs were rapidly released into the circulation and subsequently accumulated mainly in the kidney, and divalent lipid-conjugated siRNAs exhibited intermediate behavior and preferentially accumulates in the liver.^448^

### Inorganic nanoparticles

Inorganic nanoparticles mainly include metal, semiconductor, and metal oxide nanoparticles and exhibit various physicochemical properties.^449–451^ Inorganic nanoscaffolds offer access to distinctive magnetic and optical properties based on their unique physiochemical characteristics and structural capabilities.^452,453^ The inorganic nanoparticles employed for nucleic acid delivery have become a critical component in the targeted treatment of diseases. We focus on four classes of inorganic materials in this section.^454^

#### Gold

Gold is a versatile nanoparticle core for vector delivery and can form monodisperse nanostructures with high specificity.^455,456^ Gold nanoparticles (AuNPs) are tiny particles ranging from 1 to 100 nm in diameter, and their flexible sizes and shapes facilitate the loading of proteins, peptides, oligonucleotides, or small drug molecules.^457,458^ The in vivo characteristics of various AuNPs are determined by their sizes, shapes, surface charges, and surface coatings.^459–462^ For example, gold nanorods (AuNRs) were created, conjugated with CLPFFD (a brain-targeting peptide) and associated with siRNA-PARP-1, and the results revealed that this complex can cross the BBB of neonatal rats.^463^ Among the different sizes of AuNPs, medium- to large-sized AuNPs ( ≥ 10 nm) have been extensively studied due to their advantageous physical properties.^464^ A variety of coating methods, such as ligand substitution, chemical moieties or embedding in a carrier matrix, can also be applied to better employ AuNPs in pharmacology.^465^ The most widely utilized coating polymer is PEG, and PEG-coated AuNPs have a nearly neutral surface and are highly hydrophilic. Therefore, the PEG modification prevents nonspecific protein adsorption on the AuNP surface, increasing the uptake efficiency of the EPR and increasing the retention time in the circulation.^466–468^ Nucleic acid strands can be easily modified and attached to AuNP cores in a selective and cooperative manner, usually via thiol moieties.^469,470^ In early years, the synthesis and characterization of polyvalent RNA‒gold nanoparticle conjugates (RNA‒AuNPs) were reported.^471^ The particles were stable and protected from degrading enzymes, with a high efficiency in loading siRNA duplexes onto their surface, and produced robust gene knockdown.^471^ For the past few years, extensive delivery of small nucleic acids via AuNPs has been performed in animal experiments. Wan et al. developed a PEG-SH-GNP-SAPNS@miR-29a delivery system aimed at repairing injured spinal cords by creating a regenerative microenvironment that encourages the recruitment of endogenous neural stem cells.^472^ Chen et al. created aptamer-siRNA chimeras combined with PEI/PEG/AuNP/collagen membranes that can sequentially activate T cells through a layer-by-layer assembly approach.^473^ PLL is also used to decorate gold nanoparticles and efficiently deliver a Sema3A siRNA.^474^ Moreover, gold cores are inert, highly biocompatible, and safe.^459,460^ However, many aspects can be improved for an ideal AuNP drug delivery process. Prolonging the plasma circulation time, enhancing the target accumulation, improving the cellular uptake and controlling the intracellular release of AuNPs are necessary to optimize their pharmacology.

#### Silica nanoparticles (SiNPs)

Due to their advantages in terms of drug solubility and an extended release rate, SiNPs, especially porous nanoparticles, have attracted increasing interest.^475,476^ SiNPs possess tunable porous structures, excellent stability, and ease of functionalization, which make them promising platforms for nucleic acid delivery.^477,478^ SiNPs commonly offer alternative routes for medical treatment, such as inhalation, ingestion, transdermal absorption, and intravenous injection.^479^ Significant advancements have been achieved in the use of SiNPs for nucleic acid delivery and application to improve intentional modifications as well as change the surface chemistry, size, shape, and charge.^480,481^ The repulsive negative charges on the surfaces of nucleic acids and mesoporous silica nanoparticles (MSNPs) prevent them from interacting directly; thus, nucleic acids can be incorporated into MSNP pores via hydrogen bonding.^482^ Kumar et al. developed an MSNP-based multifunctional nanocarrier for the codelivery of drugs that kill drug-resistant triple-negative breast cancer cells and validated its therapeutic effects on a Shahin et al. used hyaluronic acid (HA)-modified mesoporous silica nanoparticles (MSNPs) to target CD44 on cancer cells, delivering TWIST-siRNA to inhibit TWIST protein expression in ovarian cancer cells..^483,484^ Moreover, the oral administration of only one MSNP system has been approved by the FDA.^485^ Additionally, the unique properties of MSNPs, such as their negative charge and large surface area, can be covalently modified to generate cationic substances and greatly improve drug encapsulation and release rates; PEI, dendrimers and lipids have been utilized to modify the surfaces of MSNPs for the delivery of nucleic acids.^486,487^

Moreover, prior studies have shown that exposure to SiNPs can cause adverse biological effects in various organs.^488,489^ The dose, exposure, route of administration, particle size, shape and composition have combined effects on nanoparticle toxicity.^490^ Acute toxicity is commonly observed with SiNPs, but there is a lack of information regarding their long-term toxicity. The toxicity of SiNPs depends on their physicochemical properties and is mediated by distinct mechanisms. Some researchers have confirmed that pristine SiNPs with a diameter of 50 nm caused targeted lung injury, while silica particles with a diameter of 3 μm significantly reduced the severity of lung damage. Additionally, recent evaluations have assessed the biocompatibility of SiNPs in the liver.^488^ This research group also revealed that SiNPs promoted lipid accumulation and aggravated metabolic-associated fatty liver disease (MAFLD) progression.^488^ Additionally, the cardiovascular effects of SiNP exposure have been gradually confirmed.^491–493^ A long-term study compared the acute (10-day) and subchronic (60-day and 180-day) toxicity of nonporous SiNPs with diameters of approximately 50 nm and 500 nm, as well as mesoporous SiNPs with a diameter of about 500 nm, following a single-dose intravenous injection into male and female BALB/c mice.^494^ Pathological lesions were predominantly observed with the administration of large nonporous SiNPs, while small nonporous or mesoporous SiNPs showed no significant toxicity.^495^

#### Iron oxide nanoparticles (IONPs)

IONPs contain maghemite (γ-Fe_2_O_3_) and magnetite (Fe_3_O_4_) at certain sizes, which allows them to be used as delivery vehicles.^496,497^ In recent years, various researchers have investigated the structure, magnetic properties, and biological effects of IONPs, especially surface-engineered cationic IONPs, on electrostatic interactions with nucleic acids.^498,499^ In the late 1970s, Widder and Senyi were the first to document the capability of magnetic fields to control nanoparticles for drug delivery purposes.^500,501^ This application delivers drugs directly to the brain, which is difficult to achieve using traditional methods. Small ( < 1 μm) magnetic nanoparticles can reside in glioma tissues.^502^ Recently, IONPs functionalized with peptides were used to deliver si-MGMT to glioma cells, which exhibited significantly increased sensitivity to temozolomide treatment.^503^ With the assistance of RVG (a brain-targeting peptide), biomimetic iron oxide nanorods loaded with an NF-κB siRNA targeting neurons have been developed and shown to improve memory and cognitive abilities in Alzheimer’s disease model mice.^504^ In addition, IONPs combined with engineered exosomes can penetrate the BBB and encapsulate siRNAs for GBM therapy.^505^ IONPs offer several benefits, including the ability to be magnetically directed to disease sites, tracked using contrast imaging, and precisely release drugs upon heat stimulation (Table 5).^506^

**Table 5:** Inorganic nanoparticles for small nucleic acid drug delivery

Inorganic nanoparticle	Organic modification	Disease	Nucleic acid drug	Therapeutic target	Administration route	Ref.
AuNPs	CLPFFD peptide	Perinatal asphyxia	siRNA	PARP-1	Intraperitoneal injection	^463^
AuNPs	PLL	Spinal cord injury	siRNA	SEMA3A	Lesion site injection	^474^
MSN	PLR, PEG, AS1411 aptamer	TNBC	siRNA	BCL-2	-	^483^
MSN	PEI	-	siRNA	COLL1A	Intraperitoneal injection	^495^
Iron oxide nanorod	RVG-conjugating polymer, dopamine	AD	siRNA	NF-κB	Intravenous injection	^504^

*AuNPs* gold nanoparticles, *MSN* mesoporous silica nanoparticles, *PLR* poly-L-arginine, *TNBC* triple-negative breast cancer, *PLL* Poly-l-lysin, *PEI* polyethyleneimine, *AD* Alzheimer’s Disease

### Vector-free delivery

Although delivery systems can increase uptake efficiency in vitro and alter accumulation in target organs in vivo, delicately designed small nucleic acid drugs, which can be directly used without any vectors, exhibit high biosafety, few side effects and low immunogenicity in the human body.^507^ ALN-RSV01, which targets the human respiratory syncytial virus (RSV) nucleocapsid gene, can reduce the viral titer to background levels after the intranasal instillation of the molecule with 2’-methoxylation of certain nucleotides and the addition of hydroxyproline in RSV-infected mice.^508^ SYL040012, an siRNA that targets ADRB2, can be rapidly distributed among structures of the anterior segment of the eye after direct administration in eye drops.^509^ Zheng et al. constructed an siRNA-based vehicle (siRNAsome) composed of self-assembled siRNA-disulfide-poly(N-isopropylacrylamide) diblock copolymers with a hydrophilic interior. This siRNAsome can be directly endocytosed by MDR MCF-7 cells without a transfection agent.^510^ Through the grafting of a cationic photosensitizer (NB-Br) onto siRNAs, an amphiphilic conjugate can be obtained for further self-assembly into nanoparticles. In vitro experiments revealed rapid and efficient cell endocytosis and endosomal escape; in vivo experiments revealed the inhibition of tumor growth.^511^ However, several hurdles must be overcome before the wide use of vector-free small nucleic acids in the clinic, including their easy degradation by nucleases, lack of specific delivery, poor stability and unpredictable in vivo fate.^512^

### Commercial platforms for smart small nucleic acid drug design

The landscape of small nucleic acid therapeutics has rapidly advanced, driven by innovative design platforms that enable the precise targeting of genetic material. These platforms represent the forefront of next-generation medicine.

Dicerna possesses delivery platforms capable of targeting the (GalXC™) and extrahepatic tissues (GalXC-Plus™), both of which can greatly complement Novo Nordisk’s existing therapeutic pipeline. The GalXC™ platform is a cutting-edge technology designed to precisely target the liver in drug delivery. By leveraging the high liver selectivity of GalNAc, GalXC™ constructs structurally specific siRNAs. The Dicer enzyme is the natural starting point for RNAi within human cells, and Dicerna directly links the GalNAc to the extended region of proprietary Dicer-substrate short interfering RNA (DsiRNA) molecules. This strategy allows the creation of various conjugated delivery structures that can flexibly and efficiently bind to target ligands while stabilizing the RNAi duplex, achieving precise targeting. Unlike other GalNAc delivery systems, GalXC™ introduces a unique tetraloop structure in the passenger strand, with monovalent GalNAc conjugation that enhances conjugate stability. Additionally, this design accurately positions multiple GalNAc ligands, effectively delivering siRNAs to liver cells. Furthermore, GalXC RNAi technology has exceptional pharmacological properties that significantly reduce the risks associated with drug development. GalXC-Plus builds upon the strong preclinical and clinical characteristics of the original GalXC platform, offering customizable nucleic acid structures and a variety of ligands. This platform enables RNAi drugs to be delivered to a wide range of tissues, including the central nervous system, muscles, adipose tissue, and tumors. The platform provides additional flexibility for drug optimization and expansion into new therapeutic areas. Dicerna’s Dicer-substrate RNA and tetraloop conjugation technology theoretically provide certain advantages over Alnylam’s corresponding technology. The successful completion of phase 3 clinical trials and subsequent approval of nedosiran demonstrated that GalXC™ technology is not only theoretically sound but also effective in practice.

The Arrowhead TRiM™ platform is a powerful and universal tool that employs a comprehensive array of RNAi delivery technologies, chemical modifications and structural designs. The platform utilizes specialized targeting ligands combined with unique RNA modifications to achieve the highly effective and efficient suppression of AGER gene expression. ARO-RAGE is an investigational RNAi-based drug designed using the TRiM™ platform that is intended for the treatment of inflammatory lung diseases, such as asthma. ARORAGE-1001 is an ongoing phase 1/2a randomized, double-blind, placebo-controlled study designed to evaluate the safety, tolerability, pharmacokinetics, and pharmacodynamics of ARO-RAGE in normal healthy volunteers and asthma patients (NCT05276570).

Nano-systems play a crucial role in the delivery of small nucleic acids, as they enhance the stability, targeting, and efficacy of these therapies. Each type of carrier system offers unique benefits that make it suitable for specific applications. In conclusion, leveraging the unique properties of various carrier systems can significantly enhance the effectiveness of small nucleic acid therapies, facilitating their translation from bench to clinic and back again (Table 6).

**Table 6:** The advantages of different carrier systems for application

Characteristics	Chemical structure	Preparation process	Uniformity	Tissue targeting	Advantages	Applications
LBNPs	● Amphiphilic molecules forming bilayers ● Flexibility in incorporating various lipids	● Self-assembly in aqueous environments ● Scalable and reproducible	High batch-to-batch consistency	● Enhanced by surface modification ● Passive targeting via EPR effect	● High biocompatibility ● Efficient encapsulation of both hydrophilic and hydrophobic drugs ● Ease of functionalization for targeted delivery	● Drug delivery in cancer therapy ● Gene delivery systems ● Vaccines
Peptides	● Short chains of amino acids ● High structural diversity	● Solid-phase synthesis ● Customizable sequences	High uniformity in synthesis	High specificity through sequence design	● High specificity to target sites ● Easy to synthesize and modify ● Biodegradability and low toxicity	● Targeted drug delivery ● Antimicrobial agents
Polymers	● Long chains of repeating unit ● Can be natural or synthetic	● Polymerization ● Various methods (emulsion, nanoprecipitation)	Can vary depending on synthesis method	Tunable by polymer composition and structure	● Versatile in size and shape ● Controlled drug release ● High stability and loading capacity	● Sustained drug release systems ● Tissue engineering ● Controlled drug delivery in specific conditions
Exosomes	● Lipid bilayer vesicles ● Derived from natural cells	● Isolated from biological fluids ● Requires purification steps	May have variability depending on source	● Intrinsic targeting abilities ● Homing to donor cells	● Natural origin and high biocompatibility ● Intrinsic targeting ability ● Minimal immune response	● Regenerative medicine ● Cancer therapy ● Diagnostic biomarkers and drug delivery
IONPs	● Core-shell structure with iron oxide core ● Can be coated with various materials	● Co-precipitation, thermal decomposition ● Requires precise control-	High uniformity achievable	● Can be targeted using magnetic fields ● Functionalizable for specific targeting	● Magnetic properties enabling targeted delivery ● High stability and low toxicity ● Easily functionalizable	● Magnetic resonance imaging (MRI) ● Targeted drug deliverr ● Hyperthermia treatment in cancer therapy

*LBNPs* Lipid-based nanoparticles, *IONPs* Iron Oxide Nanoparticles

## Clinical translational studies of nucleic acid therapy

**Fig. 6:**
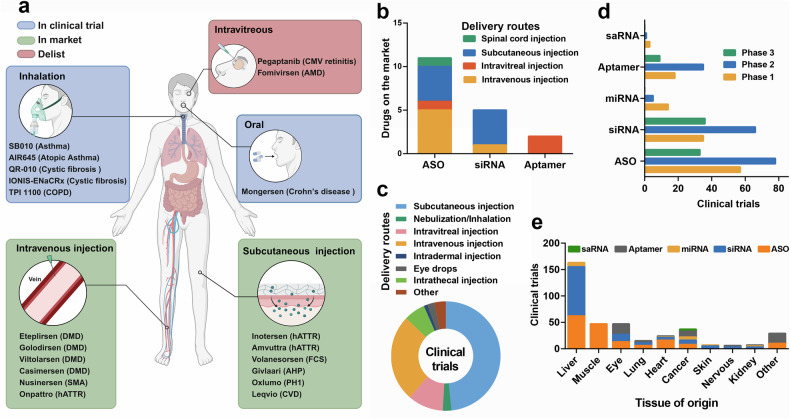
Summary of the characteristics of small nucleic acid drugs used in clinical trials. **a** Various administration routes for small nucleic acid drugs, including subcutaneous, intravitreal, intravenous, inhalation, and oral administration. Blue box: in a clinical trial; green box: on the market; red box: delisted. **b** Administration routes of small nucleic acid drugs on the market. **c** Administration routes of small nucleic acid drugs in clinical trials. **d** Different phases of clinical trials for small nucleic acid drugs. **e** Different target organs of small nucleic acid drugs in clinical trials

Small nucleic acids represent promising therapeutic options for various diseases, and their clinical translation is advancing with the approval of multiple ASOs and siRNA drugs for clinical use (Table 7). This section reviews the clinical trial outcomes and the current status of each drug that has been approved or is in the clinical trial phase (Fig. 6).

**Table 7:** Approved small nucleic acid-based therapies

Target	Generic name	Brand name	Company	Mechanism	Delivery system/Chemistry	Delivery route	Disease	Approved
CMV IE2	Fomivirsen	Vitravene®	Novartis	ASO	2ʹ-deoxy	Intravitreal injection	CMV retinitis	1988 USA (Delist)
VEGF	Pegaptanib	Macugen®	Pfizer/Eyetech	Aptamer	Pegylated	Intravitreal injection	AMD	2004 USA (Delist)
ApoB-100	Mipomersen (delist)	Kynamro®	Kynamro	ASO	2ʹ-O-methoxyethyl	Subcutaneous injection	HoFH	2013 USA
DMD	Eteplirsen	Exondys 51®	Sarepta	ASO	PMO	Intravenous injection	DMD	2016 USA
SMN2	Nusinersen	Spinraza®	Biogen	ASO	2ʹ-O-methoxyethyl	Intravenous injection	SMA	2016 USA 2019 CN
TTR	Inotersen	Tegsedi®	Ionis Pharmaceuticals	ASO	2ʹ-O-methoxyethyl	Subcutaneous injection	hATTR	2018 USA 2018 EUR
TTR	Patisiran	Onpattro®	Alnylam	siRNA	LNP	Intravenous injection	hATTR	2018 USA 2018 EUR
ALAS1	Givosiran	Givlaari®	Alnylam	siRNA	GalNAc	Subcutaneous injection	AHP	2019 USA
DMD	Golodirsen	Vyondys 53®	Sarepta	ASO	PMO	Intravenous injection	DMD	2019 USA
ApoC-3	Volanesorsen	Waylivra®	Ionis Pharmaceuticals	ASO	2ʹ-O-methoxyethyl	Subcutaneous injection	FCS	2019 EUR
DMD	Viltolarsen	Viltepso®	Nippon Shinyaku Co	ASO	PMO	Intravenous injection	DMD	2020 USA 2020 JPN
HAO1	Lumasiran	Oxlumo®	Alnylam	siRNA	GalNAc	Subcutaneous injection	PH1	2020 USA 2020 EUR
PCSK9	Inclisiran	Leqvio®	Alnylam Novartis	siRNA	GalNAc	Subcutaneous injection	CVD	2020 EUR 2021 USA
DMD	Casimersen	Amondys45®	Sarepta	ASO	PMO	Intravenous injection	DMD	2021 USA
TTR	Vutrisiran	Amvuttra®	Alnylam	siRNA	GalNAc	Subcutaneous injection	hATTR	2022 USA
C5	Avacincaptad pegol	Izervay®	Iveric Bio	Aptamer	Naked	Intravitreal injection	GA	2023 USA
SOD1	Tofersen	Qalsody®	Biogen	ASO	Naked	Spinal cord injection	ALS	2023 USA
TTR	Eplontersen	Wainua®	Ionis Pharmaceuticals	ASO	GalNAc	Subcutaneous injection	hATTR	2023 USA
LDHA	Nedosiran	Rivfloza®	NovoNordisk	siRNA	GalNAc	Subcutaneous injection	PH1	2023 USA

*CMV* cytomegalovirus, *AMD* age-related macular degeneration, *HoFH* homozygous familial hypercholesterolemia, *PMO* phosphorodiamidate morpholino oligomer, *DMD* Duchenne muscular dystrophy, *SMA* spinal muscular atrophy, *FCS* familial chylomicronaemia syndrome, *hATTR* hereditary TTR-mediated amyloidosis, *AHP* acute hepatic porphyria, *PH1* primary hyperoxaluria type 1, *CVD* cardiovascular disease, *GA* geographic atrophy, *ALS* Amyotrophic lateral sclerosis, *USA* the United States of America, *EUR* Europe, *CN* China, *JPN* Japanese

### siRNA- and ASO-based therapeutics

#### Infectious diseases

Hundreds of millions of people suffer from acute or chronic infectious diseases for which no satisfactory treatments or effective vaccinations are available. In fact, in contrast to patients with monogenic disorders or cancer, patients with infectious diseases are rarely treated with nucleic acid drugs.^513^ Moreover, among these infectious diseases, hepatitis, including HBV, HCV and HDV, is the most intensively researched disease in the context of treatment with small nucleic acid delivery systems.^514^

Among the 6 ASO drugs tested, GSK3228836, also called bepirovirsen, has entered phase III clinical trials (B-Well 1 and B-Well 2), the last step before submission for approval by the FDA, and the aims of the trial are to evaluate its safety and efficacy in treating chronic HBV (NCT05630820 and NCT05630807, respectively). This ASO drug is modified with 2ʹ-MOE to target all HBV RNAs, and this trial is expected to enroll 900 patients and be completed in 2026. Moreover, GSK3389404, also developed by GlaxoSmithKline; RO7062931, developed by Roche; and ALG-020572, developed by Aligos Therapeutics, are all GalNAc conjugates with the same target as bepirovirsen. Unfortunately, ALG-020572 was terminated because of multiple reports of a sudden increase in alanine transaminase (ALT) levels in patients enrolled in a phase 1 clinical trial, which suggested the occurrence of severe adverse events resulting from hepatotoxicity (NCT05001022). In addition to hepatitis virus RNAs, host microRNAs can also be optimal antiviral targets for treating hepatitis infection. Early studies documented that miR-122, specifically located in liver, is an essential host factor for HCV infection that can stabilize HCV RNA genomes.^515–517^ For example, RG-101 is a kind of ASO drug that target miR-122.

Most siRNA drugs target the RNA of all hepatitis viruses, whereas VIR-2218 (BRII-835 or ALN-HBV-02) and RBD-1016 target the X gene of HBV; however, RG6346 (DCR-HBVS), ARC-520 and ALG-125755 target the S gene of HBV. As a next-generation RNAi therapy from Arbutus Biopharma, AB-729 showed a significant improvement in efficacy compared with ARB-001467 and ARE-1740 in clinical trials. However, the AB-729 study was terminated early by the study sponsor for strategic reasons (NCT04820686). Moreover, although both animal and clinical trials have shown an efficient decrease in HBsAg levels after ARC-520 and ARC-521 treatment, both drugs were still terminated because of the death of nonhuman primates (chimpanzees) after administration, which might be associated with the excipient-related toxicity of the ARC-EX1 delivery system.^518–520^

Additionally, siRNA drugs have been used to treat other infectious diseases. ALN-RSV01 is a naked siRNA that was developed to be intranasally administered to silence the nucleocapsid protein transcripts of respiratory syncytial virus (RSV). In clinical trials, ALN-RSV01 was shown to have protective effects and reduce the infection rate in healthy individuals (NCT00658086), potentially leading to long-term benefits in lung transplant patients infected with RSV (NCT01065935). Additionally, MIR 19®, a siRNA-based drug, target SARS-CoV-2 RNA-dependent RNA polymerase (RdRp). The results of a phase 2 clinical trial revealed that MIR 19® decreased the recovery time of patients hospitalized with moderate COVID-19 (NCT05184127). Moreover, siRNA drugs for the treatment of Ebola virus infection include TKM-130803, TKM-100201 (TKM-EBOV-001) and TKM-100802, all of which are composed of LNPs and target L polymerase (Lpol) and Viral Protein 35 (VP35), and TKM-100201 and TKM-100802 also target VP24. However, clinical trials have shown that an intravenous infusion of 0.3 mg/kg/d TKM-130803 into adult patients with severe EVD does not improve survival compared with that of historic controls (PACTR201501000997429).^521^ Due to the corporate decision to reformulate the test product, both the TKM-100201 (NCT01518881) and TKM-100802 (NCT02041715) clinical trials were terminated.

Unexpected toxicity and pathogen escape from mutation pressure are still extraordinary challenges for small nucleic acid therapeutic in infectious diseases. Further improvement is essential to create well-tolerated and effective nucleic acid drugs.

#### Liver disease

The liver is a major organ of the reticuloendothelial system (RES) that actively participates in many physiological processes.^522^ Since liver is a central hub for most metabolic pathways, liver dysfunction can lead to systemic and liver-specific diseases.^523,524^ Owing to the development of GalNAc conjugation and the discovery of the first-pass effect, which refers to the phenomenon in which drugs greater than 300 Da in size accumulate in the liver following administration, several small ASOs and siRNAs for liver-related disorders have been approved.^524^ In this section, we discuss small nucleic acid drugs developed to treat liver disorders and focus on therapeutics for metabolic disorders such as hyperlipidemia, hemophilia, porphyria and primary hyperoxaluria.

A total of 8 RNA-based drugs, including 3 ASOs and 5 siRNAs, have been approved by the FDA or European Medicines Agency (EMA) for the treatment of liver disorders. As a trailblazer in oligonucleotide therapy, Carlsbad developed Kynamro (mipomersen), approved by the FDA in 2013 for the treatment of heterozygous familial hypercholesterolemia (HoFH) by reducing the expression of apolipoprotein B-100 (ApoB-100).^525^ Clinical trials showed that long-time mipomersen treatment can sustainedly reduce the expression of all atherosclerotic lipoproteins without serious toxicity observed (NCT00694109).^526^ In addition, inotersen (Tegsedi^TM^), which is the first RNA-targeting hATTR therapeutic, is an ASO drug launched by the Ionis Company for the treatment of hATTR-mediated amyloidosis.^527^ Inotersen can inhibit the production of transthyretin by binding to the TTR mRNA in the liver, the main TTR synthesis site, to reduce the circulating levels of TTR avoiding TTR deposition in tissues, which can further induce polyneuropathy.^528^ Phase 3 clinical trials have shown that long-term treatment with inotersen (for >3 years) slows the progression of polyneuropathy and improves the quality of life of hATTR patients without causing new safety issues (NCT01737398, NCT02175004).^529,530^ Moreover, volanesorsen is a 2ʹ-O-methoxyethyl-modified ASO drug, targeting hepatic apolipoprotein C-III (APOC3) synthesis to further reduce the plasma triglyceride concentration for the treatment of familial chylomicronaemia syndrome (FCS).^531^ Four key phase III clinical trials supporting the commercial application of volanesorsen (NCT02658175, NCT02300233, NCT02211209, and NCT02527343) have been conducted. These results showed that the administration of volanesorsen works in 77% of patients with FCS, where the triglyceride level was reduced to less than 750 mg per deciliter avoiding acute pancreatitis events; however, thrombocytopenia and injection site reactions are also common adverse events.^532,533^ As a result, the FDA rejected its marketing application on August 27, 2018, because of the risk of thrombocytopenia. Fortunately, on May 07, 2019, the EMA approved volanesorsen serve as an adjunct for FCS.

The first siRNA drug approved for marketing by the FDA in 2018 was the hATTR-mediated amyloidosis therapeutic drug patisiran (Onpattro™), which is also the only approved siRNA drug that does not rely on GalNAc but rather LNPs to target the liver.^15^ Since then, interest in siRNA therapies has increased, even surpassing the interest in ASOs. Patisiran can specifically bind to TTR mRNA at the 3’ untranslated region to further block its production in the liver.^534^ Givosiran (Givlaari™), lumasiran (Oxlumo™), vutrisiran (Amvuttra™) and inclisiran (Leqvio®) are conjugated with GalNAc. Vutrisiran is another siRNA drug used to treat hATTR whose chemical stability and liver targeting are both largely enhanced through GalNAc conjugation, which decreases the subcutaneous injection frequency to once every 3 months.^535^ In addition, givosiran was approved in 2019 for the treatment of acute hepatic porphyria (AHP), targeting the aminolevulinate synthase 1 (ALAS1) mRNA.^16^ ALAS is associated with accumulation of neurotoxic δ-aminolevulinic acid and porphobilinogen, which may trigger acute porphyria attacks.^536,537^ Lumasiran (Oxlumo™) is a siRNA drug targeting the hydroxyacid oxidase 1 gene (HAO1), for primary hyperoxaluria type 1 (PH1) by subcutaneous administration. The HAO1 gene encodes glycolate oxidase, which is involved in the synthesis of oxalate.^538^ Inclisiran (Leqvio^®^) targets the proprotein convertase subtilisin kexin type 9 (PCSK9), an enzyme secreted primarily by the liver that can modulate the degradation of the low-density lipoprotein receptor (LDLR), further influencing the concentration of LDL-cholesterol (LDL-C), a factor contributing to atherosclerotic cardiovascular disease (ASCVD).^539^ Inclisiran exhibits excellent efficacy in lowering LDL-C levels, which can lead to a half reduction in LDL-C levels in ASCVD through subcutaneous administration every 6 months.^540^

#### Ocular diseases

Since the 1990s, ASOs have been developed for effective ocular therapeutic use. The initial application of ASO therapy for ocular conditions involved injecting the IGF-I ASO during conditioned eye-blink learning in rats, which resulted in the normalization of cerebellar IGF-I levels following treatment.^541^ The delivery of ASOs to the retina has also been explored.^542^ Fomivirsen is a 21-mer ASO modified with a PS backbone, designed to target and inhibit the viral IE-2 protein.^543^ As the first approved ASO-based drug, fomivirsen treats cytomegalovirus retinitis in patients with acquired immunodeficiency syndrome (AIDS) through intravitreal (IVT) administration. Fomivirsen inhibits human cytomegalovirus replication by binding to complementary sequences on mRNAs derived from the major immediate-early transcriptional unit of virus. Treatment with fomivirsen notably slowed disease progression in patients with advanced, refractory, sight-threatening cytomegalovirus retinitis or newly diagnosed unilateral peripheral cytomegalovirus retinitis.^543^ The most commonly reported side effects of fomivirsen in clinical trials were elevated intraocular pressure and certain eye inflammation.^544^ Regrettably, fomivirsen was withdrawn from the European market in 2002 and from the United States in 2006, as more effective antiretroviral therapies for the same indication had been developed. Moreover, vascular endothelial growth factor (VEGF) is also a well-established target for treating neovascular diseases. In preclinical studies of ocular ASO therapeutics, a VEGF ASO drug was applied in vivo to inhibit choroidal neovascularization (CNV) in the eye. ASO therapy also targets inflammatory factors, and the therapeutic effects of these ASOs on keratitis, chorioretinitis and inflammation after glaucoma surgery have been assessed.

ASO therapy for patients with inherited retinal degeneration (IRD) is an effective therapeutic approach for treating *CEP290*-associated Leber’s congenital amaurosis (LCA). Two studies addressing the c.2991 + 1655 A > G mutation in *CEP290* demonstrated that ASO therapy could correct the abnormal splicing of pre-mRNAs and restore ciliation in the patients’ fibroblasts.^545,546^ Furthermore, ASO therapy has been studied for other genetic conditions, such as retinitis pigmentosa (RP) and Stargardt disease.^547^ Recently, the *USH2A* gene was targeted for RP treatment, and experimental studies showed that protein domain-specific ASO-induced dual-exon skipping restored usherin expression in the zebrafish retina, correcting photopigment mislocalization in zebrafish with *USH2A* mutations.^548^

Currently, a total of seven ASO drugs that target the eye have been progressed in human trials. QR-421a was administered to patients with RP due to mutations in exon 13 of the *USH2A* gene; however, no updates on these three trials are available. QR-110 (a target of CEP290) has been explored for the LCA administration and has shown vision improvement at 3 months and sustained visual gain at 15 months in one patient. Additionally, aganirsen, an ASO that targets *IRS1*, for ischemic central retinal vein occlusion that reduces the patient burden by topical treatment.^549^

With respect to siRNA therapies being investigated in clinical trials for eye disease, most drugs are administered via IVT injection. AGN211745 (sirna-027) is the first siRNA drug aimed at eye conditions that began clinical trials, and this siRNA drug targeted the mRNA of VEGF receptor 1 (VEGFR-1). A notable decrease in the area of neovascularization was detected in a murine model of laser-induced CNV after the IVT injection of AGN211745 in mice.^550^ Further clinical trials have investigated its value in ocular disease. A phase I/II trial (NCT00363714) administered different doses of AGN211745 to 26 neovascular age-related macular degeneration (nAMD) participants via IVT, and the participants were observed for more than 24 months. Regrettably, no study results were found. A Phase II trial, which ended in 2007, involved administering various doses of AGN211745 to 138 participants over a 2-month period, with follow-up observations extending for 24 months. However, the company decided to terminate this study early; since then, no clinical studies have focused on AGN211745. In addition to targeting VEGFR, bevasiranib directly inhibits VEGF for the treatment of nAMD and has shown clinical promise in phase III trials. However, the occurrence of serious adverse effects, such as diminished visual acuity and endophthalmitis, led to the discontinuation of one clinical trial (NCT00499590). A Phase III trial of codosiran, which was designed to target CASP2 for glaucoma treatment, was also halted due to unexplained reasons (NCT02341560).

Three trials investigated the topical administration of siRNA drugs for ocular disease treatment. Tivanisiran, which targets TRPV1, finished phase III trials in 2020 for the treatment of dry eye disease (NCT03108664). SYL040012 is thought to exert its therapeutic effect by downregulating ADRB2 production. For patients with both open-angle glaucoma (NCT02250612) and ocular hypertension (NCT00990743), SYL040012 led to a substantial decrease in intraocular pressure. SYL1801 inhibited NRARP, and the safety and tolerability of different SYL1801 doses, along with the pharmacokinetic profile of the drug, were evaluated in healthy volunteers (NCT04782271). Moreover, the safety and effects of three different doses of SYL1801 eye drops on visual acuity were evaluated in patients with wet AMD (NCT05637255). Additionally, RXI-109 (NCT02599064) and PF-0423655 (NCT01445899) may reduce the progression of subretinal fibrosis in subjects with neovascular age-related macular degeneration and diabetic macular edema, respectively; however, neither of these studies has published results. Vectors loaded with different small nucleic acids are promising for treating eye diseases in the future.

#### Lung disorders

Pulmonary diseases are prominent public health and medical problems and are among the primary causes of death globally, as anatomical barriers hinder the effective delivery of medications to the lungs.^551^ Nanomedicine has emerged as a promising solution to address the limitations of existing treatments for pulmonary diseases.^552^ Inhalation of a nanocarrier-based drug delivery system (DDS) is currently being widely investigated.^553^ A total of 8 RNA-based drugs, comprising 6 ASOs and 2 siRNAs, have been evaluated in clinical trials. For the treatment of asthma, three ASO products, TPI ASM8 (NCT01158898), SB010 (NCT01743768) and AiR645 (NCT00941577), are presently being tested in clinical trials. Another drug candidate, TPI 1100 (NCT00914433), is an unmodified ASO that inhibits phosphodiesterases 4 and 7, which are crucial in the progression of chronic obstructive pulmonary disorder (COPD).

Eluforsen, a full PS and full 2ʹ-OMe ASO developed to target the mRNA region surrounding the F508 deletion, the most common mutation leading to cystic fibrosis (CF). Its goal is to restore the function of the cystic fibrosis transmembrane conductance regulator (CFTR) protein in the airway epithelium.^554^ Preclinical data have shown that eluteforsen enhanced CFTR function in F508del-CFTR cells and animal models.^555^ This ASO has exhibited potential improvements in lung function and advantages for homozygous del-508 CF patients in two clinical trials (NCT02564354 and NCT02532764).^554,556^ The nebulization of IONIS-ENAC-2.5Rx, a PS 2ʹ-cEt ASO that aimed at reducing epithelial sodium channel (ENaC) expression in the lungs and promoting RNase H1 activity, is ongoing in a Phase II clinical trial involving CF patients (NCT04441788). According to preclinical studies, ENaC mRNA expression is obviously reduced in mouse models of CF-related lung disease, resulting in significant functional improvements.^557^ In a phase I trial, IONIS-ENAC-2.5Rx also demonstrated a significant reduction in ENaC mRNA expression in healthy volunteers who received the drug via nebulization (NCT03647228).

#### Central nervous system disorders

Most neurological diseases result from gain‑of‑function mutations; thus, ASOs are extensively utilized to suppress the expression of mutant proteins in various diseases. An intrathecal infusion of 2ʹ‑MOE‑PS ASOs aimed at the human HTT transgene in a Huntington’s disease (HD) mouse model suppressed huntingtin accumulation and delayed disease progression.^558^ Furthermore, allele-specific ASOs that are both potent and selective can correct single nucleotide polymorphisms (SNPs) that are prevalent in the HD allele.^127,559^ Unlike tominersen, WVE-120101 and WVE-120102 are experimental stereopure ASOs designed to selectively suppress mHTT by targeting SNPs associated with the CAG repeat expansion in the haplotype phase.^560^ The two ASOs currently undergoing evaluation in patients with HD in two phase Ib/IIa studies. According to Wave Life Sciences, the results of the mHTT from the PRECISION-HD trials do not justify the continued development of WVE-120102 and WVE-120101, as the occurrence of serious adverse events was higher in the 32 mg group compared to the low-dose group. Additionally, the company is currently enrolling Huntington’s disease patients for its phase 1b/2a trial of the WVE-003 (SNP3) program.

Moreover, atherosclerosis (ALS) is a progressive neuromuscular disease caused by the degeneration and impairment of neurons in motor pathways, resulting in muscle function loss and ultimately leading to death within 3 to 5 years.^561^ Tofersen received its initial approval in the USA for treating ALS in adults with mutations in *SOD1* gene. In a clinical study of the intrathecal administration of tofacersen, a PS 2′-MOE ASO was developed to decrease the levels of both wild-type and mutant SOD1 mRNA through RNase H1-mediated degradation.^562^ In a phase 1/2 ascending-dose trial (NCT02623699) that assessed cerebrospinal fluid (CSF), SOD1 concentrations showed a decline following the intrathecal administration of the highest tofersen dose over 12 weeks, with a manageable incidence of adverse events related to lumbar punctures.^563^ In a phase III trial (NCT02623699), tofersen lowered SOD1 concentrations in the cerebrospinal fluid and reduced neurofilament light chain levels in the plasma over 28 weeks. However, it did not demonstrate improvements in clinical endpoints and was associated with adverse events.^562^ ALS can also result from mutations in the *C9orf72* gene. A phase I/II study was conducted to evaluate BIIB078 (IONIS-C9Rx), a PS 2′-MOE ASO aimed at selectively degrading the mutan*t C9orf72* mRNA. Regrettably, BIIB078 did not achieve any of the secondary efficacy endpoints and failed to provide a clinical benefit. No consistent differences were observed between the 60 mg BIIB078 group and the placebo group. However, participants in the 90 mg BIIB078 cohort showed a tendency for greater reductions in secondary endpoints compared to those in the 60 mg BIIB078 group and the placebo group. Based on these disappointing results, the clinical development program for BIIB078 will be discontinued, including the ongoing open-label extension study.

Spinal muscular atrophy (SMA) is among the most common and severe genetic disorders affecting children,^564^ with the loss of function of SMN2 being the primary genetic cause of this condition. Nusinersen is a fully modified 2ʹ-MOE polysaccharide ASO that modifies the splicing of the SMN2 pre-mRNA.^565^ It promotes the production of functional full-length SMN protein throughout the spinal column and central nervous system (including motor neurons) by inducing the retention of exon 7 in *SMN2*.^566^ A few clinical trials have demonstrated that the intrathecal administration of nusinersen to SMA patients is well tolerated and results in improved motor function. Nusinersen significantly improved event-free survival and overall survival in infants with type 1 SMA, showing comparable results in patients with type 2 SMA and later-onset type 3 SMA.^567,568^ These promising outcomes from clinical trials led to the FDA’s approval of nusinersen for the treatment of SMA in both pediatric and adult patients in the United States.

siRNAs are often highly modified or encapsulated in LNPs to increase their stability. Patisiran was the first siRNA therapeutic to obtain FDA approval in 2018 for treating polyneuropathy with hATTR.^15^ However, this drug is administered intravenously and does not directly target the nervous system. Patisiran inhibits TTR production in the liver, significantly decreasing neuropathy and stopping the advancement of the disease in patients with hATTR.^569^ Patisiran is known for its immense potential as an siRNA for treating brain diseases, and many siRNA therapies were later evaluated in late clinical trials for the treatment of Alzheimer’s disease (AD), Parkinson’s disease (PD), and spinocerebellar ataxia. Revusiran is the first-generation GalNAc–siRNA with ‘Standard Template Chemistry’ (STC) that contains two terminal phosphorothioate (PS) linkages and is fully modified with 2′-F,^570^ which targets TTR. In a phase 1 study, a mean reduction in TTR levels of approximately 90% was observed with multiple revusiran doses, indicating the potential clinical use of subcutaneously administered GalNAc-siRNAs for liver-related diseases. ALN-APP was designed to treat early-onset Alzheimer’s disease (EOAD) by attaching siRNAs to a lipophilic C16 fatty acid chain, which significantly enhanced the distribution of the siRNA in the brains of rats and monkeys after intrathecal injection. In 2022, a clinical trial for ALN-APP, which could specifically target the CNS for the treatment of AD (NCT05231785), was announced.

#### Muscle diseases

Duchenne muscular dystrophy (DMD) is a severe muscle disorder caused by in *DMD* mutations, leading to progressive muscle degeneration and, in many cases, premature death.^571^ A single-exon or multiexon skipping strategy to restore dystrophin expression would be applicable for 90% of DMD mutations. A strategy involving single-exon or multiexon skipping to restore dystrophin expression could potentially address 90% of *DMD* mutations.^572^ Seven ASOs with different chemical modifications targeting DMD have progressed to clinical trials. To date, the clinical therapies available for patients with confirmed *DMD* mutations that are amenable to exon 51, 45 and 53 skipping include the approved DMD therapies casimersen, eteplirsen, golodirsen, and viltolarsen, respectively. However, only viltolarsen has provided evidence supporting a treatment-related clinical improvement, as assessed by the 6-minute walk test.^573^ The rapid development of ASO-mediated therapies for DMD opens the door for the application of nucleic acid drugs in treating a variety of diseases with well-understood pathogenesis.

#### Cancer

Over the decades, the therapeutic delivery of nucleic acids into cancer cells through gene therapy has been acknowledged as a hopeful new strategy for meeting the unmet needs in cancer care. The initial clinical trial of ASOs in cancer was a phase 2 study that began in 1993 (NCT00002592). This ASO specifically targeted G4460 in chronic myelogenous leukemia (CML), binding to the mRNA of the proto-oncogene *CMYB* and triggering RNAse H-dependent degradation.^574^ Previous experiments demonstrated that ASOs markedly reduced the proliferation and differentiation of human leukemia cells.^574,575^ Furthermore, findings from trial NCT00780052 revealed that nearly half of the CML patients who were ineligible for allografts experienced substantial decreases in *CMYB* mRNA levels following treatment with the G4460 ASO.^576^ Danvatirsen (AZD9150) is a chimeric generation 2.5 ASO (16-mer ASO) modified with phosphorothioate, aimed at degrading human *STAT3* through RNase H1 activity. In the phase 1b trial (NCT01563302), Danvatirsen demonstrated a favorable safety profile and was well-tolerated at both dosing levels. Additionally, it exhibited efficacy in a subset of pretreated patients diagnosed with diffuse large B-cell lymphoma (DLBCL).^577^ Moreover, further trials of danvatirsen have been conducted in patients with different types of cancer, but the results have not been published to date.

IONIS-AR-2.5Rx (AZD5312) is a second PS 2ʹ-cEt ASO (constrained ethyl bicyclic nucleic acid) specifically designed to target full-length, splice variant and mutated forms of AR. A phase 1 study revealed that IONIS-AR-2.5Rx is tolerable in heavily pretreated mCRPC patients, showing lasting reductions in PSA and CTC levels in some individuals (NCT02144051).^578^ IONIS-AR-2.5Rx has limited efficacy, possibly due to multiple tumor pathways and mutations. Further investigations may focus on the effectiveness of these agents in combination with other anti-androgen drugs. EZN-4176 is a 16-mer third-generation locked ASO that effectively downregulates AR expression at both the transcriptional and translational levels in androgen-sensitive and castration-resistant PCa, as demonstrated in vitro and in animal models.^579^ A phase I study of EZN-4176 in CRPC patients (NCT01337518) reported dose-limiting toxicity (DLT) with elevated ALT at 10 mg/kg on a weekly schedule, preventing dose escalation. Minimal antitumor activity was observed, leading to trial termination.

The number of siRNA drugs approved for cancer immunotherapy is steadily increasing. CALAA-01, the first targeted siRNA drug, was developed in 2008 for the treatment of advanced solid tumors. It is composed of a cyclodextrin-containing polymer (CDP), a PEG stabilization agent, human transferrin, and an *RRM2* siRNA. These components can self-assemble into nanoparticles suitable for pharmaceutical applications and can be delivered intravenously.^580^ In the case of CALAA-01, the delivery system allows for the targeted delivery of functional siRNA; however, its full potential was not evaluated in the initial phase I clinical trial (NCT00689065).^581^ Multiple patients encountered dose-limiting toxicities (DLTs) such as diarrhea, fever, and fatigue, leading to the termination of the clinical trial.^581^ The probable reason is NP instability and the degradation of various components in the bloodstream.^582^

A local drug eluter (LODER) was developed for treating pancreatic ductal adenocarcinoma (PDA). The local prolonged siRNA delivery system (Local Drug EluteR, LODER) was employed to silence mutated KRAS, and the siG12D LODER was^583^developed based on the slow release of the biodegradable polymer matrix that encompasses the siRNA.^583^ A phase 1/2a study (NCT01188785) was launched for first-line treatment of non-operable locally advanced pancreatic cancer (LAPC). Patients received a single dose of siG12D-LODER with chemotherapy, showing a reported therapeutic effect.^584^ Phase II trials are currently underway for siG12D-LODER in combination with gemcitabine+nab-paclitaxel, FOLFIRINOX, or modified FOLFIRINOX (NCT01676259).

Atu027 is a lipoplex siRNA that targets PKN3 and is formulated using positively charged liposomes composed of cationic and fusogenic lipids complexed with negatively charged PKN3 siRNA, leading to RNAi induction in the vasculature of mice following systemic administration.^585,586^ Furthermore, intravenous administration of Atu027 is considered an effective drug for preventing lung metastasis in experimental lung metastasis models and mouse models of spontaneous metastasis mouse models.^587^ The first-in-human study (NCT00938574) revealed that Atu027 was safe in patients with advanced solid tumors.^588^ The first-in-human trial (NCT00938574) confirmed the safety of Atu027 in patients with advanced solid tumors. Moreover, in an orthotopic pancreatic cancer model, Atu027 was shown to boost the antitumor effects of gemcitabine. As a result, a phase I/II combination trial for advanced pancreatic cancer (NCT01808638) is currently in progress.^588^

Tyrosine kinase EphA2 is overexpressed in tumor tissues while maintaining relatively low levels in most normal tissues, making it a promising target for cancer therapy.^589^ An EphA2-targeting DOPC-encapsulated siRNA (siRNA EphA2-DOPC) was developed for efficient siRNA delivery.^590^ In an orthotopic mouse model of ovarian cancer, the injection of siRNA EphA2-DOPC led to a significant reduction in tumor growth.^590^ Moreover, the combination of siRNA EphA2-DOPC with paclitaxel demonstrated greater tumor inhibition than treatment with paclitaxel alone.^590^ Meanwhile, no observed AEs resulting from the EphA2-DOPC siRNA were considered to have occurred at >225 μg/kg when the siRNA was given as a single intravenous injection to CD-1 mice, there was an indication of a mild to moderate inflammatory response after two weekly injections.^591^ Combined with previously reported in vivo validation data, these findings indicate that the EphA2-DOPC siRNA is effective and well tolerated. The ongoing first-in-human phase I clinical trial enrolled patients with advanced solid tumors to intravenously inject the siRNA EphA2-DOPC (NCT01591356).

MYC proteins are major drivers of human tumorigenesis.^592^ For targeting MYC, Dicer-substrate siRNA (DsiRNA) formulated in EnCore lipid nanoparticles (DCR-MYC) has demonstrated efficacy in various tumor models in vivo.^593,594^ In one patient, DCR-MYC anticancer therapy was linked to thrombotic microangiopathy in the kidney.^595^ The patient ultimately died from cancer progression one year after DCR-MYC treatment was halted.^595^ Moreover, DCR-MYC is the first MYC-targeting siRNA to undergo human trials (NCT02110563). Anthony W. Tolcher reported that DCR-MYC is well-tolerated and shows encouraging clinical and metabolic effects across a range of doses.^596^ In a phase Ib/II clinical trial (NCT02314052), DCR-MYC was evaluated in patients with advanced HCC to assess its safety and tolerability. Despite the encouraging outcomes of the phase I study supporting MYC as a therapeutic target, both studies were halted due to sponsor decisions.^597^

ALN-VSP02, consisting of an LNP loaded with two distinct siRNAs (siVEGF and siKSP), marks the first dual-targeted siRNA therapy employed in clinical trials aimed at treating solid tumors.^598,599^ Multiple doses of ALN-VSP notably extend the survival of mice with advanced orthotopic liver tumors.^600^ A phase 1 trial in patients with liver-involved solid tumors assessed the safety, tolerability, pharmacokinetics, and pharmacodynamics of ALN-VSP02, indicating that ALN-VSP02 was well tolerated at the highest dose (1.25 mg/kg) with biweekly intravenous injections.^601^ The pharmacodynamics suggest robust antitumor activity, including a complete regression of liver metastases in patients with endometrial cancer.^601^ An extension study was also performed for patients who continued ALN-VSP02 to collect long-range safety response (NCT01158079).

Arbutus Biopharma Corporation developed TKM-080301, a LNP formulation of a *PLK1* siRNA targeting human PLK1.^602^ TKM-080301 demonstrated strong antiproliferative effects and gene-specific silencing in cancer cell lines, showing antitumor activity in PDX models of tumors implanted intrahepatically or subcutaneously.^603^ Its toxicity was mainly limited to the liver and spleen in HCC patients, reflecting the distribution of the LNPs.^603^ An open‐label, multicenter, dose escalation study revealed that the antitumor effect of TKM 080301 as a single agent was limited (NCT02191878).

In recent years, extensive efforts have been dedicated to identifying critical oncogenes through genomic databases, with small nucleic acids emerging as a promising method for regulating target gene expression in precision medicine.^604^ However, challenges remain in improving delivery efficiency, targeting control, release, and reducing side effects.^605^ Nanotechnology has earned attention for enhancing precision cancer therapy, with NP-mediated delivery systems (passive, active, and endogenous targeting) effectively increasing drug accumulation in tumors.^604,606^ Thus, nucleic acid drugs are employed in the early stages of cancer treatment.

More detailed information about the LNP systems referenced in this section is cataloged in Table 8 (ASO-based therapeutics) and Table 9 (siRNA-based therapeutics).

**Table 8:** Clinical trials based on ASO therapy

	Target	Brand name	Status	Delivery system	Delivery route	Disease	Clinical Trials
Infectious diseases	All HBV RNAs	RO7062931	Phase 1 Phase 1	GalNAc LNA	Subcutaneous injection	HBV	NCT03038113 NCT03505190
	All HBV RNAs	GSK3389404	Phase 1 Phase 2	GalNAc	Subcutaneous injection	HBV	NCT02647281 NCT03020745
	All HBV RNAs	GSK3228836 (Bepirovirsen)	Phase 1 Phase 1 Phase 2 Phase 2 Phase 2 Phase 2 Phase 2 Phase 2 Phase 2 Phase 2 Phase 3 Phase 3	2ʹ-O-MOE modified	Subcutaneous injection	HBV	NCT06058390 NCT04971928 NCT06497504 NCT05330455 NCT04544956 NCT04954859 NCT04676724 NCT04449029 NCT05276297 NCT02981602 NCT05630820 NCT05630807
	All HBV RNAs	ALG-020572	Phase 1	GalNAc	Subcutaneous injection	HBV	NCT05001022
	All HBV RNA	AHB-137	Phase 1 Phase 1/2 Phase 2	Undisclosed	Subcutaneous injection	HBV	NCT05717686 NCT06115993 NCT06550128
	miR-122	RG-101	Phase2 Phase2 /	GalNAc	Subcutaneous injection	HCV	EudraCT:2015-001535-21 EudraCT:2015-004702-42 EudraCT:2016-002069-77
Liver	miR-103 /107	RG125/AZD4076	Phase 1 Phase 1	GalNAc	Subcutaneous injection	NASH T2DM With NAFLD	NCT02612662 NCT02826525
	HIF1A	RO7070179	Phase 1	ASO Naked (modified)	Intravenous injection	HCC	NCT02564614
	LPA	Pelacarsen (TQJ230)	Phase 1 Phase 1 Phase 2 Phase 2 Phase 3 Phase 3 Phase 3 Phase 3	GalNAc	Subcutaneous injection	HI Healthy volunteers Aortic Stenosis Elevated Lp (a), CVD Hyperlipoproteinemia (a) Elevated Lp (a), ASCVD Hyperlipoproteinemia (a) Lp (a), CVD	NCT05026996 NCT05337878 NCT05646381 NCT03070782 NCT05900141 NCT06267560 NCT05305664 NCT04023552
	STAT6	CDK-004	Phase1	Exosome	Intravenous injection	HCC	NCT05375604
	TTR	Eplontersen (AKCEA-TTR-LRx)	Phase 1 Phase 1/2 Phase 3 Phase 3 Phase 3	GalNAc	Subcutaneous injection	Healthy volunteers hATTR Amyloidosis hATTR-PN ATTR CM hATTR-PN	NCT04302064 NCT03728634 NCT05071300 NCT04136171 NCT04136184
	APOC-III	Olezarsen (AKCEA-APOCIII-LRx)	Phase 1 Phase 2 Phase 3 Phase 3 Phase 3 Phase 3 Phase 3 Phase 3 Phase 3	GalNAc	Subcutaneous injection	Healthy volunteers HTG, ASCVD HTG HTG, CVD HTG HTG FCS FCS FCS	NCT05579860 NCT05355402 NCT05681351 NCT05610280 NCT05079919 NCT05552326 NCT05185843 NCT05130450 NCT04568434
	DGAT2	IONIS-DGAT2Rx	Phase 2	Naked ASO (modified)	Subcutaneous injection	Hepatic Steatosis	NCT03334214
	GCGR	ISIS-GCGRRx	Phase 2 Phase 2 Phase 2	Naked ASO (modified)	Subcutaneous injection	T2DM	NCT02824003 NCT01885260 NCT02583919
	PCSK9	CIVI 007	Phase 1 Phase 2	LNA	Subcutaneous injection	Hypercholesterolemia	NCT03427710 NCT04164888
	GHR	IONIS-GHR-LRx	Phase 2 Phase 2 Phase 2	GalNAc	Subcutaneous injection	Acromegaly	NCT04522180 NCT03967249 NCT03548415
	GHR	Atesidorsen/ ATL1103	Phase 2	Naked ASO (modified)	Subcutaneous injection	Acromegaly	ACTRN12615000289516
	PTP1B	ISIS-PTP1BRx	Phase 2	Naked ASO (modified)	Subcutaneous injection	T2DM	NCT01918865
	GCCR	ISIS-GCCRRx	Phase 2	Naked ASO (modified)	Subcutaneous injection	T2DM	NCT01968265
	GCGR	ISIS-GCGRRx	Phase 2 Phase 2 Phase 2	Naked ASO (2ʹ O-MOE)	Subcutaneous injection	T2DM	NCT02824003 NCT02583919 NCT01885260
	FGFR4	ISIS-FGFR4RX	Phase 2	Naked ASO (modified)	Subcutaneous injection	Obesity	NCT02476019
Neurological and muscle diseases	KLKB1	IONIS-PKKRx	Phase 2	Naked ASO (2ʹ-O-MOE)	Subcutaneous injection	Chronic migraine	NCT03108469
	SMN2	Nusinersen	Phase 1 Phase 1 Phase 1 Phase 1/2 Phase 2 Phase 2 Phase 2 Phase 2 Phase 3 Phase 3 Phase 3 Phase 3 Phase 3 Phase 3 Phase 3 Phase 4 Phase 4 /	Naked ASO (2’-O-MOE-PS)	Intrathecal injection	SMA	NCT01494701 NCT01780246 NCT02052791 NCT01703988 NCT02386553 NCT01839656 NCT02386553 NCT02462759 NCT02193074 NCT02292537 NCT02594124 NCT05156320 NCT05386680 NCT04089566 NCT02292537 NCT04488133 NCT05522361 NCT02865109
	SCN2A	PRAX-222	Phase 1/2	Undisclosed	Undisclosed	SCN2A-associated DEE	NCT05737784
	TTR	Inotersen	Phase 2 Phase 3 Phase 2/3 Phase 3	Naked ASO (2’ O-MOE-PS)	Subcutaneous injection	Amyloidosis hATTR-PN FAP, TTR FAP, TTR	NCT03702829 NCT04136184 NCT01737398 NCT02175004
	DMD (exon 51)	Eteplirsen	Phase 1/2 Phase 1/2 Phase 2 Phase 2 Phase 2 Phase 2 Phase 2 Phase 2 Phase 2 Phase 3 Phase 3	Naked ASO (PMO)	Intravenous injection	DMD	NCT00844597 NCT00159250 NCT01396239 NCT01540409 NCT02286947 NCT02420379 NCT03985878 NCT03218995 NCT04179409 NCT03992430 NCT02255552
	DMD (exon 45)	PRO044	Phase 1/2 Phase 2	Naked ASO (2ʹ‑OMe)	Subcutaneous or intravenous injection	DMD	NCT01037309 NCT02329769
	DMD (exon 45)	PRO045	Phase 1/2	Naked ASO (2ʹ‑OMe)	Subcutaneous injection	DMD	NCT01826474
	DMD (exon 45) DMD (exon 53)	SRP‑4045 SRP‑4053	Phase 3	Naked ASO (PMO)	Intravenous injection	DMD	NCT02500381
	DMD (exon 53)	Viltolarsen	Phase 2 Phase 3 Phase 3 Phase 4	Naked ASO (PMO)	Intravitreal injection	DMD	NCT04956289 NCT04060199 NCT04768062 NCT04687020
	DMD (exon 51)	Drisapersen	Phase 2 Phase 3 Phase 3	Naked ASO (2ʹ‑OMe)	Subcutaneous or intravenous injection	DMD	NCT01910649 NCT01480245 NCT01803412
	DMD (exon 45)	DS-5141b	Phase 1/2 Phase 2	Naked ASO (2ʹ‑OMe)	Subcutaneous injection	DMD	NCT02667483 NCT04433234
	DMD (exon 51)	SRP-5051	Phase 1 Phase 1/2 Phase 2	Naked ASO (PMO)	Intravenous injection	DMD	NCT03375255 NCT03675126 NCT04004065
	DMD (exon 51)	WVE-210201	Phase 1 Phase 2/3	Naked ASO (stereopure PS, 2ʹ-F, 2ʹ-OMe)	Intravenous injection	DMD	NCT03508947 NCT03907072
	DMD (exon 51)	SQY51	Phase 1/2	ASO (tcDNA)	Intravenous injection	DMD	NCT05753462
	DNM2	DYN101	Phase 1/2	Naked ASO (2ʹ-cEt)	Intravenous injection	CNM	NCT04033159
	HTT	ISIS 443139	Phase 1/2	Naked ASO (2’ -O-MOE-PS)	Intrathecal injection	HD	NCT02519036
	HTT	RO7234292	Phase 1 Phase 2 Phase 3 Phase 3	Naked ASO (2’ O-MOE-PS)	Intrathecal injection	HD	NCT04000594 NCT03342053 NCT03761849 NCT03842969
	HTT (rs362307)	WVE-120101	Phase 1/2 Phase 1/2	Naked ASO (2ʹ-OMe stereopure PS)	Intrathecal injection	HD	NCT03225833 NCT04617847
	HTT (rs362331)	WVE-120102	Phase 1/2 Phase 1/2	Naked ASO (2ʹ-OMe stereopure PS)	Intrathecal injection	HD	NCT03225846 NCT04617860
	DMPK	ISIS-DMPKR_X_	Phase 1/2	Naked ASO (2’-O-MOE-PS)	Subcutaneous injection	DM1	NCT02312011
	SNCA	ION464	Phase 1	Naked ASO (2ʹO-MOE)	Intrathecal injection	MSA	NCT04165486
	SOD1	Tofersen	Phase 1 Phase 1 Phase 3 Phase 3 Phase 3	Naked ASO (2’-O-MOE-PS)	Intrathecal injection	ALS	NCT01041222 NCT03764488 NCT02623699 NCT03070119 NCT04856982
	C9orf72	IONIS-C9Rx	Phase 1	Naked ASO (2’-O-MOE-PS)	Intrathecal injection	ALS	NCT04288856
	ATXN2	BIIB105	Phase 1/2	Naked ASO (2ʹ-O-MOE)	Intrathecal injection	ALS	NCT04494256
	LRRK2	BIIB094	Phase 1	Naked ASO (2ʹ-O-MOE)	Intrathecal injection	PD	NCT03976349
	SCN1A	STK-001	Phase 1/2 Phase 2	ASO-TANGO	Intrathecal injection	DS	NCT04442295 NCT04740476
	MAPT	IONIS-MAPTRx	Phase 1/2	Naked ASO (2’ -O-MOE-PS)	Intrathecal injection	AD	NCT03186989
Eye	USH2A	QR-421a	Phase 1/2 Phase 2/3 Phase 2/3	Undisclosed	Intravitreal injection	RP	NCT03780257 NCT05158296 NCT05176717
	CFB	IONIS-FB-L_Rx_	Phase 2 Phase 2 Phase 2	GalNAc (2ʹ-O-MOE)	Subcutaneous injection	Primary IgA Nephropathy GA GA	NCT04014335 NCT03815825 NCT03446144
	CEP290	QR-110	Phase 1/2 Phase 1/2 Phase 2 Phase 2 Phase 2/3 Phase 2/3	Naked ASO (2ʹ-OMe)	Intravitreal injection	LCA LCA AMD, GA Primary IgA Nephropathy LCA LCA	NCT03140969 NCT03913130 NCT03815825 NCT04014335 NCT03913143 NCT04855045
	IRS-1	Aganirsen	Phase 2/3	Naked ASO	Topical eye drops	iCRVO	NCT02947867
	RHO	QR-1123	Phase1/2	Naked ASO	Intravitreal injection	ADRP	NCT04123626
	TGFβ2	ISTH0036	Phase 1	Naked ASO	Intravitreal injection	POAG	NCT02406833
Lung	CCR3 IL-3, IL-5, GM-CSF receptors	TPI ASM8	Phase 2 Phase 2 Phase 2 Phase 2	Naked ASO (PS)	Inhalation	Asthma	NCT00822861 NCT00402948 NCT01158898 NCT00550797
	GATA-3	SB010	Phase 1 Phase 1 Phase 1 Phase 2	Naked ASO (modified)	Inhalation	Asthma	NCT01470911 NCT01577953 NCT01554319 NCT01743768
	CFTR, FΔ508	QR-010	Phase 1 Phase1/2	Naked ASO (2ʹ-OMe-PS)	Intranasal	CF	NCT02564354 NCT02532764
	ENaC	IONIS-ENaCRx	Phase 1 Phase 2	Naked ASO (2ʹ-cEt)	Inhalation	CF COPD	NCT03647228 NCT04441788
	IL-4/IL-13	AIR645	Phase 1 Phase 2	Naked ASO (2ʹ-O-MOE)	Nebulization	Asthma	NCT00658749 NCT00941577
	phosphodiesterase 4/7	TPI 1100	Phase 1	Naked ASO (PS)	Inhalation	COPD	NCT00914433
Cardiovascular diseases	Apo(a)	Pelacarsen	Phase 1 Phase 2 Phase 2 Phase 3 Phase 3 Phase 3 Phase 3	GalNAc (2ʹ-O-MOE)	Subcutaneous injection	HI Aortic Stenosis CVD CVD Hyperlipoproteinemia (a) ASCVD Hyperlipoproteinemia (a)	NCT05026996 NCT05646381 NCT03070782 NCT04023552 NCT05900141 NCT06267560 NCT05305664
	TTR	AKCEA-TTR-LR_x_	Phase 1 Phase 1/2 Phase 3 Phase 3 Phase 3	GalNAc (2ʹ-O-MOE)	Subcutaneous injection	Healthy volunteers hATTR Amyloidosis hATTR-PN ATTR CM hATTR-PN	NCT04302064 NCT03728634 NCT04136184 NCT04136171 NCT05071300
	ANGPTL	Vupanorsen	Phase 1 Phase 1 Phase 2 Phase 2	GalNAc (2ʹ-O-MOE)	Subcutaneous injection	Healthy volunteers Healthy volunteers NAFLD Dyslipidaemias	NCT04916795 NCT04459767 NCT03371355 NCT04516291
	ApoC-III	AKCEA-APOCIII-LR_x_ (Olezarsen)	Phase 2 Phase 2 Phase 3 Phase 3 Phase 3 Phase 3 Phase 3 Phase 3 Phase 3	GalNAc (2ʹ-O-MOE)	Subcutaneous injection	HTG; CVD ASCVD; HTG FCS FCS FCS SHTG SHTG HTG; AVD; SHTG	NCT03385239 NCT05355402 NCT05130450 NCT04568434 NCT05185843 NCT05681351 NCT05552326 NCT05610280 NCT05079919
	AGT	IONIS-AGT-LR_x_	Phase 2 Phase 2 Phase 2 Phase 2 Phase 2	GalNAc (2ʹ-O-MOE)	Subcutaneous injection	Mild Hypertension Healthy volunteers Hypertension Hypertension Chronic HFrEF	NCT03714776 NCT03101878 NCT04083222 NCT04714320 NCT04836182
	FXI	IONIS FXI-LR_x_ /BAY2306001	Phase 1 Phase 2	Naked ASO (2ʹ-O-MOE)	Subcutaneous injection	Healthy volunteers ESRD	NCT03582462NCT02553889
	Factor B	IONIS-FB-LR_x_	Phase 2	GalNAc (2ʹ-O-MOE)	Subcutaneous injection	Primary IgAN; GA	NCT04014335 NCT03815825
	miR-103/miR-107	AZD4076	Phase 1 Phase 1	Undisclosed	Subcutaneous injection	T2DM With NAFLD NASH	NCT02826525 NCT02612662
Cancer	HSP27	OGX-427	Phase 2	Naked ASO (2ʹ-O-MOE)	Intravenous injection	Bladder Cancer	NCT01454089
	STAT3	IONIS-STAT3Rx	Phase 1/2	Naked ASO (2ʹ-cEt)	Intravenous injection	Advanced cancers	NCT01563302
	STAT3	Danvatirsen	Phase 1 Phase 1 Phase 2 Phase 2 Phase 2	Naked ASO (2ʹ-cEt)	Intravenous injection	MDS/AML NSCLC NSCLC NSCLC HNSCC	NCT05986240 NCT03819465 NCT03794544 NCT02983578 NCT05814666
	AR	AZD5312	Phase 1 Phase 1	Naked ASO (2ʹ-cEt)	Intravenous injection	Prostate cancer AR tumors	NCT03300505 NCT02144051
	miR-155	Cobomarsen	Phase 1 Phase 2 Phase 2	LNA	Intratumoral/ /Subcutaneous injection/ Intravenous injection Intravenous injection	CTCL; MF; CLL; DLBCL; ATLL CTCL; MF CTCL; MF	NCT02580552 NCT03713320 NCT03837457
	GRB2	Prexigebersen	Phase 1 Phase 1 Phase 2	Liposome (P-ethoxy)	Intravenous injection	AML, CML, solid tumours CML, AML, ALL, MDS AML	NCT04196257 NCT01159028 NCT02781883
	CLU	Custirsen	Phase 1 Phase 1 Phase 1/2 Phase 2 Phase 2 Phase 2 Phase 2 Phase 3 Phase 3 Phase 3	Naked ASO (2ʹ-O-MOE)	Intravenous injection	Cancer Cardiac Conduction and Repolarization NSCLC BCa Prostate Cancer Prostate Cancer Prostate Cancer Prostate Cancer NSCLC Prostate Cancer	NCT01497470 NCT01874561 NCT00138658 NCT00258375 NCT00138918 NCT00258388 NCT00327340 NCT01188187 NCT01630733 NCT01578655
	AR	EZN-4176	Phase 1	LNA	Intravenous injection	PCa	NCT01337518
	AR	AZD5312 (ARRx)	Phase 1 Phase 1	Naked ASO (2ʹ-cEt)	Intravenous injection	Prostate Cancer Advanced Solid Tumours	NCT03300505 NCT02144051
	IGF-1R	IGV-001	Phase 2	Undisclosed	Implanted with biodiffusion chambers	Glioblastoma	NCT04485949
	GRB2	Prexigebersen (BP1001)	Phase 1 Phase 1 Phase 2	Liposome	Intravenous injection	CML, AML, ALL and MDS Solid tumors AML	NCT01159028 NCT04196257 NCT02781883
	IRF4	ION251	Phase 1	Undisclosed	Intravenous injection	RRMM	NCT04398485
	YAP1	ION537	Phase 1	Undisclosed	Intravenous injection	Advanced solid tumors	NCT04659096
	FOXP3	AZD8701	Phase 1	Naked ASO (modified)	Intravenous injection	ccRCC; NSCLC; TNBC; HNSCC; SCLC; GEC; Melanoma; Cervical Cancer and Advanced Solid Tumors	NCT04504669
Immune diseases	ICAM1	Alicaforsen	Phase 1 Phase 2 Phase 2 Phase 3 Phase 3 Phase 3	Undisclosed	Enema	IBD Ulcerative colitis Ulcerative colitis Pouchitis Crohn’s Disease Crohn’s Disease	NCT03473626 NCT00063830 NCT00063414 NCT02525523 NCT00048295 NCT00048113
	SMAD7	Mongersen	Phase 1 Phase 1 Phase 2 Phase 2 Phase 3 Phase 3	Naked ASO (phosphorothioate)	Oral	Healthy volunteers Crohn’s Disease Crohn’s Disease Colitis, Ulcerative Crohn’s Disease Crohn’s Disease	NCT02957474 NCT02367183 NCT02685683 NCT02601300 NCT02596893 NCT02641392
Other diseases	TTR	Eplontersen	Phase 1 Phase 1/2 Phase 3 Phase 3 Phase 3 Phase 3 Phase 3	GalNAc (2ʹ-O-MOE,)	Subcutaneous injection	Healthy volunteers hATTR Amyloidosis hATTR-PN ATTR CM hATTR-PN ATTR CM ATTR CM	NCT04302064 NCT03728634 NCT04136184 NCT05667493 NCT05071300 NCT06194825 NCT04136171
	KLKB1	Donidalorsen	Phase 1 Phase 2 Phase 2 Phase 3 Phase 3	Naked ASO (2ʹ-O-MOE)	Subcutaneous injection	HAE	NCT03263507 NCT04307381 NCT04030598 NCT05139810 NCT05392114
	TMPRSS6	Sapablursen	Phase 2 Phase 2	LICA	Subcutaneous injection	Polycythemia vera BT intermedia	NCT05143957 NCT04059406
	miR-17	RGLS4326	Phase 1	Undisclosed	Subcutaneous injection	Autosomal dominant; polycystic kidney disease	NCT04536688
	miR-21	Lademirsen (RG-012)	Phase 1 Phase 2	Chemically modified	Subcutaneous injection	Alport syndrome	NCT03373786 NCT02855268
	COL7A1 (exon 73 mutation)	QR-313	Phase 1/2	Undisclosed	Topical cream	Recessive and dystrophic EB	NCT03605069

*GalNAc* N-acetylgalactosamine, *LNA* locked nucleic acid, *HBV* Hepatitis B virus infectious, *2ʹ-O-MOE* 2’-O-(2-methoxyethyl), *HCV* Hepatitis C virus infectious, *NASH* Non-alcoholic fatty liver disease, *T2DM* Diabetes mellitus type 2, *NAFLD* Non-alcoholic fatty liver disease, *HCC* Hepatocellular carcinoma, *HI* Hepatic impairment, *hATTR-PN* Hereditary Transthyretin-Mediated Amyloid Polyneuropathy, *ATTR CM* Transthyretin-Mediated Amyloid Cardiomyopathy, *FAP* Familial amyloid polyneuropathy, *HTG* Hypertriglyceridemia, *FCS* Familial chylomicronemia syndrome, *CVD* cardiovascular disease, *HAE* Hereditary angio-oedema, *PS* modified phosphorothioate, *SMA* Spinal muscular atrophy, *SCN2A* Sodium voltage-gated channel alpha subunit 2, *DEE* Developmental and epileptic encephalopathy, *PMO* Phosphorodiamidate morpholino oligomers, *DMD* Duchenne muscular dystrophy, *2ʹ-OMe* 2ʹ-methoxy, *2ʹ-F* 2ʹ-fluoro, *tcDNA* Tricyclo-DNA, *2ʹ-cEt* 2ʹ-constrained ethyl, *CNM* Centronuclear myopathy,*HD* Huntington’s disease, *DM1* Myotonic dystrophy type 1, *MSA* Multiple system atrophy, *ALS* Amyotrophic lateral sclerosis, *PD* Parkinson diseas, *DS* Dravet syndrome, *AD* Alzheimer’s disease, *RP* Retinitis pigmentos, *GA* Geographic atrophy, *LCA* Leber’s congenital amaurosis, *iCRVO* Ischaemic central retinal vein occlusion, *ADRP* Autosomal dominant retinitis pigmentosa, *POAG* Primary open-angle glaucoma, *CF* Cystic fibrosis, *COPD* Chronic obstructive pulmonary disease, *CAVS* Calcific aortic valve stenosis, *ASCVD* Arteriosclerotic cardiovascular disease, *SHTG* Severe hypertriglyceridemia, *RHTN* Resistant hypertension, *HFrEF* Heart failure with reduced ejection fraction, *ESRD* End-stage Renal Disease, *IgAN* Immunoglobulin A (IgA) nephropathy, *HF* Heart failure, *MDS* Myelodysplastic syndromes, *AML* Acute myeloid leukaemia, *NSCLS* non-small-cell lung cancer, *HNSCC* head and neck squamous cell carcinomas, *AR* Androgen receptor, *CTCL* Cutaneous T-cell lymphoma, *MF* Mycosis fungoides, *CLL* Chronic lymphocytic leukemia, *DLBCL* Diffuse large B-cell lymphoma, *ATLL* Adult T-cell leukemia/lymphoma, *ALL* acute lymphocytic leukaemia, *BCa* Bladder cancer, *PCa* prostate cancer, *CML* chronic myelogenous leukaemia, *RRMM* Relapsed/refractory multiple myeloma, *ccRCC* clear cell renal cell carcinoma, *TNBC* Triple negative breast cancer, *HNSCC* Head and neck squamous cell carcinoma, *SCLC* Small Cell Lung Cancer, *GEC* Gastroesophageal cancer, *IBD* Inflammatory bowel disease, *BT* Beta-thalassemia, *EB* Epidermolysis bullosa

**Table 9:** siRNA-based therapies currently in clinical trials

	Target	Brand name	Phase	Delivery system	Delivery route	Disease	Clinical trials
Infectious diseases	All HBV RNAs	AB-729	Phase 2 Phase 2 Phase 2 Phase 2	GalNAc	Subcutaneous injection	Chronic HBV Chronic HBV Chronic HBV, HDV Chronic HBV	NCT04980482 NCT04820686 NCT04847440 NCT06154278
	X gene	VIR-2218	Phase 1/2 Phase 1/2 Phase 2 Phase 2 Phase 2 Phase 2 Phase 2 Phase 2 Phase 2 Phase 2	Ga1NAc	Subcutaneous injection	Chronic HBV Chronic HBV Chronic HBV Chronic HBV Chronic HBV Chronic HBV Chronic HDV Chronic HBV Chronic HBV Chronic HBV	NCT03672188 NCT05612581 NCT06092333 NCT04507269 NCT04412863 NCT04856085 NCT05461170 NCT05970289 NCT04749368 NCT04891770
	S gene	RG6346 (DCR-HBVS)	Phase1	GalNAc	Subcutaneous injection	Chronic HBV	NCT03772249
	All HBV RNAs	JNJ-73763989 (JNJ-3989) (ARO-HBV)	Phase 1 Phase 1 Phase 1 Phase 1 Phase 2 Phase 2 Phase 2 Phase 2 Phase 2 Phase 2 Phase 2	GalNAc	Subcutaneous injection	Chronic HBV Chronic HBV Chronic HBV Hepatic impairment Chronic HBV Chronic HBV Chronic HBV Chronic HBV and HDV Chronic HBV HBV Chronic HBV	NCT05123599 NCT04002752 NCT04586439 NCT04208386 NCT05275023 NCT04667104 NCT05005507 NCT04535544 NCT04439539 NCT04585789 NCT03982186
	All HBV RNAs	ARC-520	Phase 1 Phase 1 Phase 2 Phase 2 Phase 2 Phase 2 Phase 2 Phase 2	Polymer	Intravenous injection	Chronic HBV Chronic HBV Chronic HBV HBV HBV Chronic HBV HBV, HDV Chronic HBV	NCT02535416 NCT01872065 NCT02738008 NCT02604199 NCT02604212 NCT02452528 NCT02577029 NCT02065336
	All HBV RNAs	ARC-521	Phase 1	Polymer	Subcutaneous injection	HBV	NCT02797522
	X gene	RBD-1016	Phase 1 Phase 1 Phase 2	GalNAc	Subcutaneous injection	Chronic HBV	NCT05017116 NCT04685564 NCT05961098
	All HBV RNAs	ARB-001467	Phase2	LNP	Intravenous injection	Chronic HBV	NCT02631096
	S gene	ALG-125755	Phase 1	GalNAc	Subcutaneous injection	Chronic HBV	NCT05561530
	All HBV RNAs	TQA3038	Phase 1 Phase 1/2	GalNAc	Subcutaneous injection	Chronic HBV	NCT06085053 NCT06452693
	Undisclosed	HRS-5635	Phase 1 Phase 2	Undisclosed	Subcutaneous injection	Chronic HBV	NCT05808374 NCT06425341
	Lpol, VP35	TKM-130803	Phase2	LNP	Intravenous injection	Ebola Virus Infection	PACTR201501000997429
	Lpol, VP24, and VP35	TKM-100201 (TKM-EBOV-001)	Phase1	LNP	Intravenous injection	Ebola Virus Infection	NCT01518881
	Lpol, VP24, and VP35	TKM-100802	Phase1	LNP	Intravenous injection	Ebola Virus Infection	NCT02041715
Liver	AAT	ARC-AAT	Phase 1	ARC-EX1	Intravenous injection	AATD	NCT02363946
	Antithrombin	Fitusiran (ALN-AT3SC)	Phase 1 Phase 1/2 Phase 3 Phase 3 Phase 3 Phase 3 Phase 3 Phase 3	GalNAc	Subcutaneous injection	Hemophilia A Hemophilia A and B Hemophilia Hemophilia Hemophilia A and B Hemophilia A and B Hemophilia Hemophilia	NCT06145373 NCT02554773 NCT03974113 NCT03754790 NCT03417245 NCT03417102 NCT03549871 NCT05662319
	LDH	Nedosiran (DCR-PHXC)	Phase 1 Phase 2 Phase 2 Phase 2 Phase 3 /	GalNAc	Subcutaneous injection	PH	NCT04555486 NCT04580420 NCT05001269 NCT03847909 NCT04042402 NCT05993416
	PNPLA3	ALN-PNP	Phase 1 Phase 1	Undisclosed	Subcutaneous injection	NAFLD	NCT06024408 NCT05648214
	HSD17B13	ALN-HSD	Phase 1 Phase 2	GalNAc	Subcutaneous injection	NASH	NCT04565717 NCT05519475
	HBV X gene	VIR2218	Phase 1	Ga1NAc	Subcutaneous injection	Hepatic Impairment Cirrhosis	NCT05484206
	GO	DCR-PH1	Phase 1	LNP	Intravenous injection	PH Type 1	NCT02795325
	TTR	Revusiran (ALN-TTRSC)	Phase 1 Phase 2 Phase 2 Phase 2 Phase 3	GalNAc	Subcutaneous injection	hATTR hATTR, FAP hATTR hATTR hATTR	NCT01814839 NCT02595983 NCT02292186 NCT01981837 NCT02319005
	TTR	Patisiran	Phase 1 Phase 1 Phase 1 Phase 2 Phase 2 Phase 3 Phase 3 Phase 3 Phase 3 Phase 3 / / / /	LNP	Intravenous injection	hATTR	NCT05023889 NCT02053454 NCT01559077 NCT01961921 NCT01617967 NCT01960348 NCT02510261 NCT03862807 NCT03997383 NCT03759379 NCT03431896 NCT04201418 NCT05040373 NCT05873868
	FXI	RBD4059	Phase 1	GalNAc	Subcutaneous injection	Thrombotic diseases	NCT05653037
	PCSK9	SGB-3403	Phase 1	GalNAc	Subcutaneous injection	Hyperlipidemias	NCT06239714
	PCSK9	Inclisiran	Phase 2 Phase 2 Phase 3 Phase 3 Phase 3 Phase 3 Phase 3 Phase 3 Phase 3 Phase 3 Phase 4 Phase 4 Phase 4 Phase 4	Chemically modified siRNA	Subcutaneous injection	ASCVD ASCVD; T2DM; FH ASCVD HeFH or HoFH ASCVD ASCVD HeFH HoFH ASCVD ASCVD Hypercholesterolemia Hypercholesterolaemia; PH ASCVD	NCT02597127 NCT03060577 NCT04929249 NCT05682378 NCT06494501 NCT03814187 NCT03397121 NCT03851705 NCT03400800 NCT03399370 NCT06431763 NCT06501443 NCT06386419 NCT06249165
	APOC3	Plozasiran	Phase 3 Phase 3 Phase 3 Phase 3	GalNAc	Subcutaneous injection	FCS HTG Severe HTG Severe HTG	NCT05089084 NCT06347133 NCT06347003 NCT06347016
	Lipoprotein(a)	LY3819469 (Lepodisiran)	Phase 1 Phase 1 Phase 1 Phase 2 Phase 3	GalNAc	Subcutaneous injection	Healthy Healthy Renal insufficiency Lipoprotein disorder ASCVD; Elevated Lp(a)	NCT04914546 NCT05932446 NCT05841277 NCT05565742 NCT06292013
	Lipoprotein(a)	Olpasiran (AMG 890)	Phase 1 Phase 1 Phase 1 Phase 1 Phase 2 Phase 3	GalNAc	Subcutaneous injection	Basic science RI Elevated Lp(a) Hepatic impairment CVD ASCVD	NCT06411860 NCT05489614 NCT04987320 NCT05481411 NCT04270760 NCT05581303
	ANGPTL3	ARO-ANG3	Phase 1 Phase 2 Phase 2	GalNAc	Subcutaneous injection	Dyslipidemias; FH; HTG Mixed dyslipidemia HoFH	NCT03747224 NCT04832971 NCT05217667
	HAO1	Lumasiran	Phase 2 Phase 2 Phase 2 Phase 3 Phase 3 Phase 3 / / /	GalNAc	Subcutaneous injection	PH type 1 CKD; CVD; PH Recurrent KSD PH type 1 PH type 1 PH type 1 PH PH type 1 PH type 1	NCT03350451 NCT06225544 NCT05161936 NCT03905694 NCT03681184 NCT04152200 NCT04125472 NCT04982393 NCT06225882
	KHK	ALN-KHK	Phase 1/2	GalNAc	Subcutaneous injection	T2DM	NCT05761301
Eye	NRARP	SYL1801	Phase 1 Phase 2	Naked siRNA	Eye drops	Wet AMD	NCT04782271 NCT05637255
	CTGF	RXI-109	Phase 1/2	Naked siRNA	Intravitreal injection	Wet AMD	NCT02599064
	RTP801	PF-0423655	Phase 2	Naked siRNA	Intravitreal injection	DME CNV DR	NCT01445899
	CASP2	Codosiran (QPI-1007)	Phase 1 Phase 2 Phase 2/3	Naked siRNA	Intravitreal injection	APAC APACG NAION	NCT01064505 NCT01965106 NCT02341560
	ADRB2	Bamosiran (SYL040012)	Phase 1 Phase 2	Naked siRNA	Eye drops	OHT OAG	NCT00990743 NCT02250612
	VEGFR1	AGN211745	Phase 1/2 Phase 2 (Terminated)	Naked siRNA	Intravitreal injection	CNV, AMD ARMD	NCT00363714 NCT00395057
	TRPV1	Tivanisiran (SYL1001)	Phase 3 Phase 3 Phase 3	Naked siRNA	Eye drops	DED	NCT03108664 NCT05310422 NCT04819269
	VEGF	Bevasiranib	Phase 1 Phase 2 Phase 2 Phase 3	Naked siRNA	Intravitreal injection	MD DME Wet AMD	NCT00722384 NCT00259753 NCT00306904 NCT00499590
Lung	RSV	ALN-RSV01	Phase 2 Phase 2 Phase 2b	Naked siRNA	Nebulization	RSV	NCT00658086 NCT00496821 NCT01065935
	SARS-CoV-2 RdRp	MIR 19 ®	Phase 2 Phase 2/3	Peptide dendrimer KK-46	Inhalation	COVID-19	NCT05184127 NCT05783206
	SARS-CoV-2	siCoV/ KK46	Phase 1	Peptide dendrimer	Inhalation	COVID-19	NCT05208996
Kidney	P53	Teprasiran	Phase 3	Naked siRNA	Intravenous injection	DGF with kidney allografts	NCT03510897
	P53	QPI-1002	Phase 1 Phase 1/2 Phase 2 Phase 3 Phase 3	Naked siRNA	Intravenous injection	AKI DGF AKI DGF DGF	NCT00683553 NCT00802347 NCT02610283 NCT03510897 NCT02610296
	PCSK9	Inclisiran	Phase 1	Chemically modified siRNA	Subcutaneous injection	RI	NCT03159416
	HBV X gene	VIR2218	Phase 1	Ga1NAc	Subcutaneous injection	RI	NCT05844228
Nervous system	APP	ALN-APP	Phase 1 Phase 2	2’-O-hexadecyl modified	Intrathecal injection	EOAD CAA	NCT05231785 NCT06393712
	BCL2L12	NU-0129	Phase 1	SNA	Intratumoral injection	Recurrent GBM or GS	NCT03020017
	SOD1	RAG-17	Phase 1	SCAD™	Lumbar spine injection	ALS	NCT05903690
Heart	AGT	Zilebesiran (ALN-AGT01)	Phase 1 Phase 1/2 Phase 2 Phase 2 Phase 2	GalNAc	Subcutaneous injection	Hypertension Mild-to-moderate hypertension Hypertension High cardiovascular risk, Hypertension	NCT03934307 NCT06423352 NCT04936035 NCT05103332 NCT06272487
	PCSK9	Inclisiran	Phase 4 Phase 4	Chemically modified siRNA	Subcutaneous injection	ACS; IHD CAD	NCT06421363 NCT06338293
Skin	CTGF	RXI-109	Phase 1 Phase 1 Phase 2 Phase 2	Naked siRNA	Incision injection	Transverse HTS	NCT01640912 NCT01780077 NCT02030275 NCT02079168
	CTGF	OLX10010	Phase 1 Phase 2	cp-asiRNA	Intradermal injection	Recurrence of HTS	NCT03569267 NCT04877756
	CTGF	BMT101	Phase 1 Phase 2	cp-asiRNA	Intradermal injection	HTS	NCT03133130 NCT04012099
	TGFB1 and COX-2	STP705	Phase 1 Phase 1 Phase 1/2 Phase 1/2 Phase 1/2 Phase 1/2 Phase 2 Phase 2 Phase 2	HKP	Dry powder for intra-and peri-lesional injection	Abdominal obesity HCC Facial isSCC isSCC HTS HTS BCC Keloid isSCC	NCT05422378 NCT04676633 NCT05421013 NCT04293679 NCT02956317 NCT05196373 NCT04669808 NCT04844840 NCT04844983
	K6a	TD101	Phase 1	Naked siRNA	Intralesional injection into a plantar callus	PC	NCT00716014
Cancer	RRM2	CALAA-01	Phase1	AD-PEG-Tf	Intravenous injection	Advanced solid tumors	NCT00689065
	KRAS	siG12D LODER	Phase1 Phase2	Polymeric NPs (LODER)	EUS biopsy needle	Operable PDA Locally advanced PDA	NCT01188785 NCT01676259
	PKN3	Atu027	Phase1 Phase1/2	Cationic lipids	Intravenous injection	Advanced solid tumor Advanced or metastatic PDA	NCT00938574 NCT01808638
	EphA2	siRNA EphA2-DOPC	Phase1	Neutral liposome (DOPC)	Intravenous injection	Advanced or recurrent solid tumor	NCT01591356
	MYC	DCR-MYC	Phase1 Phase1b/2	Lipid nanoparticle	Intravenous injection	Solid tumors, multiple myeloma, or lymphoma HCC	NCT02110563 NCT02314052
	VEGF and SKP	ALN-VSP02	Phase1 Phase1	LNP	Intravenous injection	Advanced solid tumor	NCT00882180 NCT01158079
	PLK1	TKM-080301	Phase 1 Phase1/2 Phase 1/2	LNP	Intravenous injection	Primary or secondary HCC Advanced HCC Cancer	NCT01437007 NCT02191878 NCT01262235

*GalNAc* N-acetylgalactosamine, *HBV* Hepatitis B virus infectious, *HDV* Hepatitis D virus infectious, *LNP* Lipid nanoparticle, *AATD* Alpha-1 Antitrypsin Deficiency, *PH* Primary Hyperoxaluria, *NASH* Nonalcoholic Steatohepatitis, *hATTR* Hereditary transthyretin-mediated amyloidosis, *FAP* Familial Amyloidotic Polyneuropathy, *ASCVD* Atherosclerotic Cardiovascular Disease, *T2DM* Type 2 diabetes mellitus, *FH* Familial Hypercholesterolemia, *HoFH* Homozygous Familial Hypercholesterolemia, *HeFH* Heterozygous familial hypercholesterolemia, *FCS* Familial chylomicronemia syndrome, *HTG* Hypertriglyceridemia, *Lp(a)* Lipoprotein(a), *RI* Renal impairment, *CVD* Cardiovascular Disease, *CKD* Chronic Kidney Disease, *AMD* Age related macular degeneration, *DME* Diabetic macular edema, *CNV* Choroidal neovascularization, *DR* Diabetic retinopathy, *APAC* Acute primary angle closure, *APACG* Acute primary angle-closure glaucoma, *NAION* Non-arteritic anterior ischemic optic neuropathy, *OHT* Ocular hypertension, *OAG* Open-angle glaucoma, *DED* Dry eye disease, *MD* Macular degeneration, *RSV* Respiratory syncytial virus, *COVID-2019* Coronavirus disease, *DGF* Delayed graft function, *AKI* Acute kidney injury, *EOAD* Early-onset alzheimer’s disease, *CAA* Cerebral amyloid angiopathy, *SNA* Spherical nucleic acid, *GBM* glioblastoma multiforme, *GS* gliosarcoma, *ALS* Amyotrophic lateral sclerosis, *ACS* Acute coronary syndrome, *IHD* Ischemic heart disease, *CAD* Coronary artery disease, *HKP* Histidine-lysine polypeptide copolymer, *HTS* Hypertrophic scars, *HCC* Hepatocellular carcinoma, *isSCC* in situ squamous cell carcinoma, *BCC* Basal cell carcinoma, *PC* Pachyonychia congenita, *AD-PEG-Tf* Adamantane–Polyethylene glycol–transferrin, *PDA* Pancreatic adenocarcinoma, *DOPC* 1,2-Dioleoyl-sn-glycero-3-phosphocholine

## Aptamer-based therapies

Due to their unique three-dimensional structure, aptamers can recognize target molecules through their three-dimensional conformation and exhibit high binding affinity. Aptamer-based drugs include a variety of technologies, featuring novel shapes, chemistries, and delivery methods, and are utilized for both therapeutic and diagnostic purposes.^607^

Aptamers are chemically synthesized in vitro, resulting in shorter synthesis times, lower costs, greater stability, and greater specificity. The small and flexible structure of aptamers allows them to bind to smaller targets or hidden epitopes that some antibodies cannot access. Over the last few years, aptamer-based drugs that have garnered significant interest in clinical applications as alternatives to traditional monoclonal antibody-based therapies have been developed. Pegaptanib, developed by Valeant, was the first nucleic acid aptamer drug approved for the treatment of wet age-related macular degeneration and received FDA approval in 2004. It was subsequently approved by the EMA in January 2006 and by the PMDA in July 2008.

Structurally, pegaptanib is a 28-base RNA oligonucleotide conjugated to a 20 kDa branched PEG.^608^ Mechanistically, pegaptanib selectively binds to the VEGF 165 isoform, thereby slowing the development of choroidal neovascularization and reducing leakage from abnormal blood vessels.^609^ Preclinical data indicate that the drug is metabolized in vivo by endonucleases and exonucleases and is excreted in the urine in both its original form and as a metabolite. Pharmacokinetic studies of IVT in rhesus monkeys have shown that drug concentrations in the vitreous and blood are dose dependent and that the drug’s half-life is also dose dependent, following first-order kinetic processes.^610^ In a phase II clinical trial, a multicenter, open-label, repeated-dose study involving 21 patients with choroidal neovascularization secondary to AMD was conducted over 3 months. The results revealed that 87.5% of patients who received pegaptanib monotherapy experienced stable or improved vision, with 25% of treated eyes gaining 3 or more lines of visual acuity. Additionally, 60% of patients treated with a combination of pegaptanib and photodynamic therapy (PDT) had visual acuity improvements of 3 lines or more, suggesting a potential synergistic effect between the two treatments.^611^ These findings confirmed the safety of the drug, with no clear drug-related complications observed.

Avacincaptad pegol is also an RNA aptamer. Structurally, it is composed of 39 bases, with a PEG modification at the 5’ end, a capped structure at the 3’ end, and a molecular weight of nearly 56 kDa.^612^ It can efficiently and specifically bind to complement C5 in patients with AMD and received FDA approval for geographic atrophy in 2023.^612^ In a phase II AMD study (NCT03362190), 43 subjects received monthly doses of 0.3, 1, or 2 mg of avacincaptad pegol along with 0.5 mg of Lucentis (OphthoTech Corporation). OphthoTech announced results from a phase 2a safety trial of Zimura® in combination with Lucentis® for wet age-related macular degeneration (media release, 16 Nov 2018). After 6 months, the mean improvement in visual activity was 13.6, 11.7, and 15.3 letters for patients receiving low, medium, and high doses, respectively, with 46%, 47%, and 60% of patients improving by more than 3 lines. Compared with the control group receiving Lucentis alone, a significant portion of patients in the combination therapy group experienced improved vision.

To date, most therapeutic aptamers are still in preclinical or early clinical development stages. Aptamer drugs that target ophthalmic diseases, cardiovascular conditions, tumors, and inflammation have already entered clinical trials. AS1411 is the first aptamer to enter clinical trials for cancer treatment. Its nucleic acid sequence is rich in guanine, which easily forms a quadruplex structure.^613^ This unique structure not only resists nuclease degradation but also inhibits the proliferation of cancer cells by specifically targeting nucleolin.^614,615^ In a phase I clinical trial involving patients with advanced cancer (NCT00881244), AS1411 was well tolerated after six months of treatment, with no adverse reactions observed, except in patients with renal cell carcinoma. However, in the phase II clinical trial, only limited efficacy was observed, leading to the suspension of the trial (NCT00740441).

NOX-A12 binds to and neutralizes CXC chemokine ligand (CXCL12), disrupting the CXCL12 gradient established by bone marrow stromal cells and ultimately sensitizing CLL cells to cytotoxic drugs.^616^ The FDA granted NOX-A12 the orphan drug designation for use in combination with radiation therapy for patients with glioblastoma in 2014. This aptamer is currently being developed under the trade name olaptesed pegol for the treatment of various malignancies. In a recent phase I/II clinical trial (NCT01486797), 28 patients with relapsed/refractory chronic lymphocytic leukemia received olaptesed pegol in combination with bendamustine and rituximab.^617^ The monotherapy was well tolerated, with no dose-limiting toxicity observed, and the overall response rate for the combination therapy was 86%, with 11% of patients achieving complete remission and 75% achieving partial remission.^617^ In animal experiments, the PEGylated L-RNA aptamer olaptesed pegol was highly effective in a rat brain tumor model of highly refractory glioblastoma (GBM). An open-label, multicenter phase I/II trial known as the GLORIA trial was conducted to evaluate the clinical safety and efficacy of combining radiotherapy with NOX-A12 (NCT04121455). This study reported the safety of RT and NOX-A12 in patients with newly diagnosed chemotherapy-resistant GBM, achieving the primary endpoint of the trial.^618^

DTRI-031 is a dose-dependent platelet aggregation-inhibiting aptamer capable of reopening vessels occluded by platelet-rich thrombi.^619^ A randomized, double-blind, single-center, placebo-controlled phase I study is underway in healthy volunteers to assess the safety, tolerability, pharmacokinetics, and pharmacodynamics of a single intravenous injection of DTRI-031 (NCT05005520).

Several coagulation aptamers are being investigated in different stages of clinical trials. REG1 is an aptamer-based factor IXa inhibitor developed for percutaneous coronary intervention (PCI) in patients with acute coronary syndrome.^620^ It consists of the RNA aptamer pegnivacogin (RB006, with a 40 kDa polyethylene glycol carrier attached to its tail) and the antidote anivamersen (RB007). A phase I clinical trial (NCT00113997) of REG1 for anticoagulation showed positive results, with lower toxicity during PCI, but the phase II trial was prematurely terminated due to severe allergic reactions (NCT01848106), and the system is currently being optimized.^621^

ARC1779 is the first DNA aptamer that targets the A1 domain of von Willebrand factor (vWF), with indications primarily for thrombotic thrombocytopenic purpura, von Willebrand disease, cerebrovascular embolism, and thrombotic microangiopathy.^622^ Preliminary evidence suggests that low doses of ARC1779 can be used to correct vWF and/or FVIII deficiencies in patients with hereditary bleeding disorders. A clinical trial (NCT00432770) evaluated the safety, pharmacokinetics, and pharmacodynamics of ARC1779 in patients with VWF-related platelet function disorders. These results indicate that ARC1779 can inhibit platelet aggregation without significantly increasing the risk of bleeding.^622^ A phase II clinical trial (NCT00742612) investigated the effect of ARC1779 on cerebral microemboli in patients immediately after carotid endarterectomy; however, the study had to be paused because of insufficient patient enrollment.

Due to their high binding specificities and affinities, as well as several advantages over antibodies, aptamers have become excellent alternatives to antibodies in the treatment of various diseases. Currently, attention to therapeutic aptamers is increasing significantly annually, with several aptamer-based drugs undergoing proof-of-concept studies and various stages of clinical trials, and they have already shown great potential in the treatment of serious diseases (Table 10).

**Table 10:** Aptamer therapeutics in clinical trials

Tissue	Target	Brand name	Status	Delivery route	Disease	Clinical trials
Eye	VEGF	EYE001	Phase 1 Phase 2/3 Phase 2/3 Phase 3 Phase 2 Phase 2	Intravitreal injection	VHL AMD Neovascular AMD DME AMD	NCT00056199 NCT00321997 NCT00021736 NCT00150202 NCT00040313 NCT00239928
	PVDF	Fovista	Phase 1 Phase 2a Phase 2 Phase 3 Phase 3 Phase 3	Intravitreal injection	Neovascular AMD AMD Neovascular AMD AMD	NCT02591914 NCT02387957 NCT02214628 NCT01944839 NCT01940900 NCT01940887
	C5	Zimura	Phase 2 Phase 2 Phase 2 Phase 2 Phase 2 Phase 2/3 Phase 3 Phase 3	Intravitreal injection	IPCV Neovascular AMD AMD STGD1 GA secondary to AMD GA	NCT02397954 NCT03374670 NCT03362190 NCT05571267 NCT03364153 NCT02686658 NCT04435366 NCT05536297
Blood system	vWF	ARC1779	Phase 1 Phase 2 Phase 2 Phase 2 Phase 2 Phase 2	Intravenous injection	von Willebrand disease Thrombotic microangiopathy Cerebral microembolism Heart attack.	NCT00432770 NCT00632242 NCT00694785 NCT00726544 NCT00742612 NCT00507338
	TFP1	ARC19499	Phase 1	Subcutaneous injection	Hemophilia	NCT01191372
	Factor IX	REG1	Phase 1 Phase 1/2 Phase 2 Phase 2 Phase 3	Intravenous injection	Thrombosis Hematologic malignancies CAD Acute Coronary Syndrome CAD	NCT00113997 NCT01050764 NCT00715455 NCT00932100 NCT01848106
	vWF	BT200	Phase 1 Phase 2	Subcutaneous injection	Hereditary bleeding disorders	NCT04103034 NCT04677803
	Hepcidin	NOX-H94	Phase 1 Phase 1 Phase 1/2 Phase 2	Subcutaneous injection Intravenous injection	Anemia Anemia of chronic disease in patients with cancer	NCT01372137 NCT01522794 NCT02079896 NCT01691040
Cancer	Nucleolin	AS1411	Phase 1 Phase 2 Phase 2 Phase 2	Intravenous injection	Advanced solid tumors Metastatic renal cell carcinoma Acute myeloid leukemia	NCT00881244 NCT00740441 NCT00512083 NCT01034410
	PTK7	68Ga-Sgc8	Phase 1 NA	Intravenous injection	Colorectal cancer Bladder cancer	NCT03385148 NCT06005116
Immune system	CCL2	NOX-E36	Phase 1 Phase 1 Phase 1/2 Phase 2	Subcutaneous injection Intravenous injection	Chronic inflammatory diseases Renal impairment Type 2 diabetes mellitus	NCT00976729 NCT01372124 NCT01085292 NCT01547897
	CXCL12	NOX-A12	Phase 1 Phase 1 Phase 1/2 Phase 1/2 Phase 2 Phase 2 Phase 2	Intravenous injection	Autologous stem cell transplantation Colorectal and pancreatic cancer Glioblastoma Multiple myeloma CLL Pancreatic cancer	NCT00976378 NCT01194934 NCT03168139 NCT04121455 NCT01521533 NCT01486797 NCT04901741
Cardiovascular system	Thrombin	NU172	Phase 2	Intravenous injection	Heart disease	NCT00808964
Respiratory system	TLR4	ApTOLL	Phase 1	Intravenous injection	COVID-19	NCT05293236
	C5a	AON-D21	Phase 1 Phase 1 Phase 2	Intravenous injection	Community-acquired pneumonia Healthy Community-acquired pneumonia	NCT05343819 NCT05018403 NCT05962606
Nervous system	TLR4	ApTOLL	Phase 1 Phase 1 Phase 1/2	Intravenous infusion vs. bolus intravenous injection Intravenous injection	Stroke	NCT05569720 NCT04742062 NCT04734548

*VHL* Von Hippel-Lindau syndrome, *AMD* Age-related macular degeneration, *DME* Diabetic macular edema, *MD* Macular degeneration, *IPCV* Idiopathic polypoidal choroidal vasculopathy, *GA* geographic atrophy, *STGD1*Stargardt disease, *CAD* Coronary artery disease, *RADAR* Acute coronary syndrome, *HSCT* Hematopoietic peripheral blood stem cell transplant, *CLL* Chronic lymphocytic leukemia, *COVID-19* Coronavirus disease

### MicroRNA therapeutics

To date, miRNAs have been demonstrated to play a role in the pathogenesis of human diseases, especially viral infection, metabolic disorders and cancer.^623,624^ The main function of miRNAs is to suppress gene expression by binding to the 3’-UTRs (untranslated regions) of mRNAs in a post-transcriptional manner.^625^ Advancements in miRNA research concerning human diseases has allowed miRNAs to hold great prognostic value and to become therapeutic agents.^626,627^ Here, we present a summary of miRNA-related preclinical development and clinical trials, and miRNA mimics and miRNA inhibitors currently show promise as novel therapeutic drugs (Table 11).

**Table 11:** miRNA and saRNA therapeutics in clinical trials

	Tissue	Target	Brand name	Status	Delivery system	Delivery route	Disease	Clinical trials
miRNA	Liver	miR-122	Miravirsen	Phase 1 Phase 2 Phase 2 Phase 2	LNA modified	Subcutaneous injection	Hepatitis C Chronic HCV CHC	NCT01646489 NCT01727934 NCT01872936 NCT02452814 NCT02508090 NCT01200420
	Heart	miR-132	CDR132L	Phase 1	LNA modified	Intravenous injection	Heart failure	NCT04045405
	Skin	miR-29	MRG-201	Phase 1 Phase 1	Cholesterol conjugated	Intradermal injection	Scleroderma	NCT02603224 NCT03601052
		miR-92a	MRG-110	Phase 1	LNA modified	Skin injection	Wound	NCT03603431
	Cancer	miR-221	LNA-i-Mir-221	Phase 1	LNA modified	Intravenous injection	Refractory-MM, advanced solid tumors	NCT04811898
		miR‑155	MRG-106	Phase 1 Phase 2 Phase 2	LNA modified	Intratumoral Injection	MF, CLL, DLBCL or ATLL CTCL	NCT02580552 NCT03713320 NCT03837457
		miR‑16	MesomiR-1	Phase 1	Nonliving bacterial minicells	Intravenous injection	Recurrent MPM and NSCLC	NCT02369198
		miR-34a	MRX34	Phase 1 Phase 1/2	LNP	Intravenous injection	HCC Melanoma	NCT01829971 NCT02862145
		miR-193a-3p	INT-1B3	Phase 1	LNP	Intravenous injection	Advanced solid tumors	NCT04675996
	Kidney	miR-17	RGLS8429	Phase 1 Phase 1	Not mentioned	Subcutaneous injection	ADPKD	NCT05521191 NCT05429073
saRNA	Tumor	C/EBPα	MTL-CEBPA	Phase 1 Phase 1 Phase 1 Phase 2	SMARTICLES® liposomal nanoparticle	Intravenous injection	HCC Solid tumors Advanced HCC HCC, HBV, HCC	NCT02716012 NCT04105335 NCT05097911 NCT04710641

*LNA* locked nucleic acid, *HCV* Hepatitis C virus infection, *CHC* Chronic hepatitis C virus infection, *T2DM* Type 2 diabetes mellitus, *NAFLD* Non-alcoholic fatty liver disease, *MF* Mycosis fungoides, *CLL* chronic lymphocytic leukemia, *DLBCL* diffuse large B-cell lymphoma, *ATLL* adult T-cell leukemia/lymphoma, *CTCL* Cutaneous T-cell lymphoma, *MPM* Malignant pleural mesothelioma, *NSCLC* Non-small cell lung cancer, *LNP* Liposome nanoparticle, *HCC* Hepatocellular carcinoma, *ADPKD* Autosomal dominant polycystic kidney disease, *HBV* Hepatitis B virus infection

The first miRNA-targeted drug to advance into clinical development was miravirsen, a locked nucleic acid-modified oligonucleotide that designed to antagonize miR-122 for treating hepatitis C virus (HCV) infection. Patients with chronic HCV infection who received miravirsen demonstrated sustained, dose-dependent decreases in *HCV* levels, with no signs of viral resistance or long-term safety issues.^628–630^ This treatment, which makes the reality of miRNA therapy undeniable, facilitated the progression of miravirsen into further studies involving long-term follow-up, a larger patient population, and multi-drug combinations. RG-101 is a modified phosphorothioate oligonucleotide that inhibits miR-122 and is conjugated to a multivalent N-GalNAc structure, specifically engineered to improve oligonucleotide absorption by hepatocytes. This drug has undergone phase I trials in patients infected with HCV.^444,631,632^ While RG-101 led to a significant decrease in viral load among all treated patients, the trial was terminated by the FDA due to reports of jaundice.

The most advanced cancer-targeting miRNA drug is MRX34, a synthetic miR-34a mimic, double-stranded and encapsulated within the NOV40 lipid carrier.^633^ A preclinical study revealed that systematic injection of MRX34 markedly inhibited tumor growth and significantly increased the survival of mouse models with orthotopic hepatocellular carcinoma, and no immunostimulatory effects or dose-limiting toxicities were observed.^634,635^ The first clinical trial of miRNA therapy conducted on humans was the Phase 1 clinical trial (NCT01829971) of MRX34, which enrolled 155 participants diagnosed with seven distinct cancer types, such as primary liver cancer, SCLC, lymphoma, melanoma, multiple myeloma, renal cell carcinoma, and NSCLC.^636,637^ Another clinical trial (NCT02862145) evaluated the therapeutic effects of MRX34 in combination with dexamethasone on melanoma patients.

Many preclinical and clinical trials focused on miRNAs have since been conducted, describing the potential of miRNAs as therapeutic agents. The disparity between foundational research on miRNAs and their practical use in clinical settings continues to be considerable. A phase 1 clinical trial of the miR-29 mimic MRG-201 for the treatment of scleroderma was conducted in 2021 (NCT02603224). In addition, MRG-229 is a modified miR-29 mimic that features enhanced stability due to conjugation to the internalization moiety BiPP, was found to be a potent candidate therapeutic for fibrotic conditions in the preclinical stage.^638^ The drug MRG-110 is a locked ASO of miR-92a, which focuses on angiogenesis during wound healing and demonstrates potential therapeutic benefits for both chronic and acute wounds.^639^ A phase 1 clinical trial (NCT03603431) showed that a systemic infusion of MRG-110 effectively inhibited miR-92a in human blood.^640^ Activation of cardiac miR-132 results in adverse remodeling and pathological hypertrophy. A preclinical study assessed the safety and effectiveness of CDR132L in a clinically relevant pig model of chronic heart failure.^641^ CDR132L treatment markedly reduced the expression of fibrosis markers and reversed cardiac remodeling in a phase Ib trial (NCT04045405). The encouraging efficacy and suitable tolerance of these regimens provide a basis for further clinical research to further confirm the beneficial effects of CDR132L treatment.^642^ MRG-106, a miR-155 inhibitor, is synthesized as an LNA-modified oligonucleotide and can be administered via subcutaneous injection, intravenous infusion, or directly into cancerous skin lesions.^643,644^ Patients diagnosed with specific lymphomas and leukemias were recruited, and this trial was the first to utilize miRNA inhibitors instead of the previously tested miRNA mimics. Although the phase 1 results (NCT02580552) were encouraging, the phase 2 clinical trials (NCT03713320 and NCT03837457) were halted early by the sponsoring company for commercial reasons. LNA-i-Mir-221, MesomiR-1, and INT-1B3 were all systemically administered and completed phase 1 trials. However, the cellular effects of miRNAs are so extensive that off-target effects are inevitable. Therefore, the primary challenges for effectively tackling these issues require further development of synthetic RNA technologies and specific delivery systems to ensure the safe and targeted administration of miRNA therapeutics.

### Small activating RNA therapeutics

Unlike other gene overexpression techniques, such as plasmids, viruses or mRNAs, saRNAs are small, versatile and safe, with lower overall research costs.^645^ Thus, saRNAs constitute an alternative category of therapeutics that can restore the expression of essencial genes in disease conditions. A growing number of animal and clinical studies have investigated saRNA-based therapies for multiple diseases (Table 11).

Several in vivo studies have validated the therapeutic effectiveness of saRNAs in animal models. To induce endogenous p21 expression, Li et al. targeted a 19-nucleotide saRNA at the –322 position of the p21 promoter relative to the transcription start site and synthesized it into a novel saRNA, p21-saRNA-322, according to design guidelines.^105^ p21-saRNA-322 successfully hindered the growth of several cancer models, including PCa, HCC, NSCLC, pancreatic cancer and bladder cancer.^646–651^ However, the major obstacle to successful saRNA therapy is the absence of efficient drug delivery systems. A 2’-fluoro-modified derivative (dsP21-322-2’F) of LNPs was formulated for intravesical drug delivery, which led to increased urothelial uptake and prolonged survival in mice with established orthotopic human bladder cancer.^652^ Moreover, a rectal delivery system, TSLPP-p21-saRNA-322, consisting of a PEI/p21-saRNA-322 polyplex core and a hyaluronan-modulated lipid shell, was developed to treat colorectal cancer.^653^ Although p21-saRNA-322 induced substantial tumor shrinkage in animal models, intratumor delivery of the encapsulated saRNAs may delay the application of the findings into a clinical practice. Although most saRNA-based drugs are still in the preclinical stage, MTL-EBPA is the cutting-edge therapeutic saRNA developed by the National University Cancer Institute (NCIS) and MiNA Therapeutics, entered a phase 2 trial in March 2016.^654^ MTL-EBPA is intended for targeting solid tumors and is composed of SMARTICLE liposomal nanoparticles and 2′-O-Me saRNAs, which can increase the expression of the *C/EBPα* gene. *C/EBPα* is a transcription factor strongly implicated in myelopoiesis and the proliferation and differentiation of cancer cells.^655–658^ In preclinical studies, evidence has shown that injectable saRNAs that successfully effectively enhance C/EBPα expression, decrease tumor burden, and ameliorate liver function in a cirrhotic rat model of multifocal liver tumors.^659^ Another targeted delivery system for C/EBPα-saRNAs, employing RNA aptamers specific to pancreatic ductal adenocarcinoma (PDAC), has shown antiproliferative effects in vivo^660^; further studies have shown that linking C/EBPα-saRNAs to TR14 could offer promising therapeutic effects on advanced PDAC.^661^ These comprehensive and exciting preclinical studies prompted the development of clinical trials. The initial phase 1a/b clinical trial of MTL-CEBPA in humans revealed a favorable safety and tolerability profile for patients with hepatocellular carcinoma linked to hepatitis B and/or C infections (NCT02716012).^662^ Moreover, this trial demonstrated that pretreatment with MTL-CEBPA ameliorates the immunosuppressive HCC microenvironment to enhance the treatment benefits of tyrosine kinase inhibitors (TKIs).^663^ Thus, many clinical trials of the combination of MTL-CEBPA with other small-molecule drugs, such as PD-1 inhibitors (NCT04105335), atezolizumab and bevacizumab (NCT05097911), have been conducted.^612^ Moreover, the combination of MTL-CEBPA and sorafenib has entered a phase 2 clinical trial (NCT04710641).

## Clinical feedback and drug tailoring in development

To date, small nucleic acid drugs have achieved remarkable success in clinical trials. Additionally, an increasing number of pharmaceutical companies are now engaged in researching and developing small nucleic acid-based therapies for the treatment of various diseases. The integration of clinical feedback significantly enhances the drug development process.^664,665^ By analyzing data from clinical trials, researchers can refine drug formulations to better meet patient needs. This iterative process ensures that therapies are not only effective but also safe and well-tolerated.

During the initial phases of development, preclinical studies offer essential insights into the potential efficacy and safety of drugs. However, the data collected during clinical trials truly inform the refinement process. Patient responses, side effects, and therapeutic outcomes all contribute valuable insights.^666^ These insights allow researchers to adjust dosages, modify delivery mechanisms, and even alter the chemical structure of the drug to optimize drug performance. Furthermore, tailoring drugs based on the clinical data involves a deep understanding of patient demographics and genetic profiles. Personalized medicine has become a key focus, as variations in the genetic makeup can significantly impact how patients respond to treatments.^667^ By incorporating these data, researchers can develop more targeted therapies that are effective for specific patient groups, reducing the trial-and-error approach traditionally associated with drug development.^668^

The incorporation of clinical feedback not only improves the efficacy of drugs but also accelerates the approval process. Patient clinical data provide information for laboratory research to realize the rapid transformation of the results of the study. Regulatory agencies are more likely to approve drugs that demonstrate clear benefits and minimal risks and are supported by comprehensive clinical data. This streamlined process helps bring new therapies to market faster, benefiting patients who need them the most. Ultimately, the continuous loop of feedback and adjustment creates a robust framework for developing small nucleic acid therapies. By prioritizing clinical data, researchers ensure that each iteration of a drug is better suited to meet the clinical needs, laying the foundation for more effective and personalized treatments.

The transition from Onpattro® to Vutrisiran® represents an advancement in small nucleic acid drug development that benefitted from clinical feedback. Building on the success of Onpattro®, researchers have applied the lessons learned from the clinical limitations identified in Onpattro® to enhance the next generation of therapies.^669,670^ Vutrisiran® exemplifies how integrating patient data can lead to improved drug formulations with greater efficacy and safety.^569^ This continuous cycle of feedback and improvement underscores the importance of patient-centric approaches in drug development. Onpattro® requires intravenous infusion every three weeks, meaning that patients need to visit the hospital frequently for treatment, increasing both time and financial burdens. In contrast, Vutrisiran® has a relatively lower dosing frequency, requiring subcutaneous injection every three months.^535,670^ This schedule not only reduces the treatment frequency for patients but also decreases the occurrence of adverse reactions, thereby improving patients’ quality of life.^535,670^ Moreover, drawing from the experiences with inotersen, researchers have improved their methods to boost the efficacy and safety of eplontersen. The design of eplontersen reflects a more patient-centric approach, addressing the limitations observed with inotersen. Eplontersen shares a similar design and identical nucleobase sequence with inotersen, an ASO previously approved for the treatment of polyneuropathy of ATTR, but uses unique chemistry and GalNAc conjugation to specifically target hepatocytes, the main source of TTR in the body.^671,672^ This targeted approach allows eplontersen to be administered at lower doses and less frequently while still achieving efficacy.^671,672^

In recent years, the clinical use of small nucleic acid drugs has decreased, whereas laboratory research on these drugs has increased, which is marked by a resurgence in fundamental investigations. This shift reflects a strategic pivot toward refining the foundational aspects of small nucleic acid therapeutics. By conducting more basic science experiments, researchers aim to deepen their understanding of molecular mechanisms, optimize drug designs, and innovate delivery strategies.^673^ This iterative process promises to fortify the clinical pipeline, ensuring that future advancements are grounded in robust scientific insights and paving the way for transformative treatments in diverse therapeutic landscapes. Insights gained from patient outcomes and clinical data can be used to tailor drug development to meet real-world needs.^674^ This feedback loop allows researchers to make necessary adjustments, increasing the efficacy and safety of small nucleic acid therapeutics. The integration of clinical experiences with laboratory research fosters a dynamic environment where theoretical knowledge and practical application inform each other.

## Future directions for small nucleic acid therapeutics

**Fig. 7:**
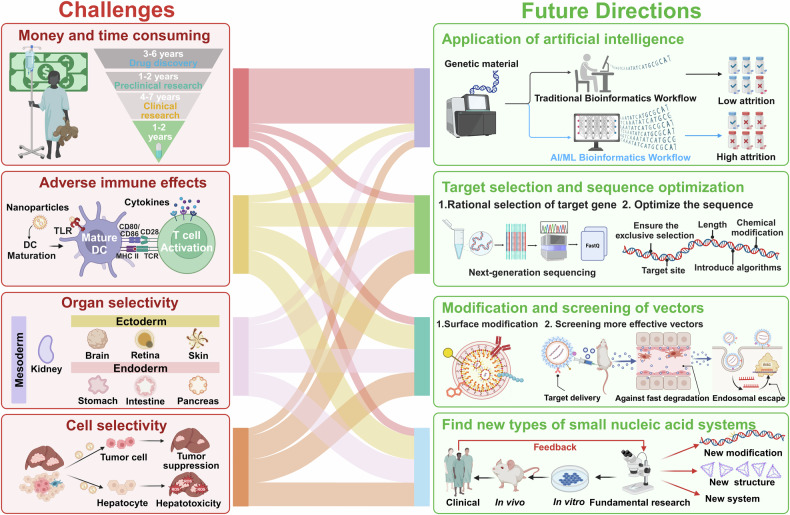
The challenges and future directions of small nucleic acid drugs. Various challenges in the development of small nucleic acid drugs persist (red box). First, the development of new drugs is expensive and time-consuming. In addition, NPs may cause adverse immune effects, such as an inflammatory cytokine storm. Moreover, cost and regulatory challenges exist. In addition, insufficient organ and cell selectivity reduce the effectiveness of small nucleic acid drugs. Many ideas have been proposed to solve these barriers as future directions for small nucleic acid drugs (green boxes). The first is the application of artificial intelligence (AI) in drug discovery. The second is improving the safety profile, which should focus on both the delivery vector and the sequence of small nucleic acids. The third is surface modifications of nucleic acid drugs for delivery to specific cell types. Finally, vectors are screened to deliver nucleic acid drugs more efficiently

Small nucleic acid therapeutics represent an innovative approach, leveraging the precise gene expression modulation abilities of these molecules through their binding affinity to specific RNA sequences. With a deeper understanding of genes and RNA structures, along with ongoing advancements in biotechnology, the prospects for small nucleic acid therapeutics are highly promising. Over the past years, preclinical and clinical data have confirmed the potential of small nucleic acid therapies to treat various diseases. However, several advances are needed to fully reach their potential (Fig. 7).

### Current limitations and shortcomings

Despite the significant progress in the development of small nucleic acid therapies, several limitations and shortcomings persist. These issues hinder the full realization of their potential as next-generation medicines.

#### Financial and time constraints

The process of translating these drugs from the laboratory to the clinic is notably time intensive.^675,676^ Extensive research, rigorous testing, and multiple phases of clinical trials are needed to ensure safety and efficacy. In addition to the time involved, the cost of developing small nucleic acid drugs is a major barrier.^677^ The complexity of drug design and production, coupled with stringent regulatory requirements, contributes to the high financial burden. These expenses can limit the ability of researchers and companies to sustain long-term development efforts and restrict access to these therapies once they are approved. Moreover, the financial risks associated with this lengthy development process can deter investment in these innovative drugs. The high cost and uncertainty of success may discourage potential investors, further slowing progress.

#### Unintended immune reactions

Unlike small-molecule drugs, small nucleic acid drugs primarily exert their effects by binding to target mRNAs once inside the body. Oligonucleotides generally share certain molecular characteristics with naturally occurring oligonucleotides, which may result in immunotoxicity.^678^ However, during the development process, these compounds are often chemically modified to increase their stability, safety, cellular uptake, and efficacy.^167^ According to the literature, the mechanism of immunogenic reactions is due primarily to the binding of oligonucleotide drugs to pattern recognition receptors (e.g., TLRs), which activate the innate immune system, leading to an immune response, and the immunomodulatory effect of oligonucleotides is influenced mainly by their sequences, with guanine- and uridine-rich sequences being more prone to binding to TLR7/8.^679–681^ Additionally, some modifications, such as PS modifications, promote inflammatory responses, which are known to induce immunogenic reactions.^682^ Thrombocytopenia has occasionally been observed in preclinical models, particularly for PS-ASOs such as volanesorsen and inotersen.^533,683^ PS backbone modifications have been proven to be one of the major causes of reduced platelet counts. Studies have reported that PS-ASOs specifically bind to platelet glycoprotein VI, which in turn activates human platelets, triggering the formation of platelet‒leukocyte aggregates.^684^

Moreover, nanoparticle toxicity remains a significant concern in the development of small nucleic acid drugs. For example, SiO2 NPs have been shown to cause toxicity in various human organs, including lung epithelial cells, liver cells, and intestinal cells, as well as in the lungs and kidneys.^685^ Although research on nanomaterial toxicity has yielded mixed results regarding its extent and mechanisms, some materials previously deemed biocompatible due to the safety of their bulk forms can clearly be toxic. Factors such as the nanomaterial size, shape, surface chemistry, and aggregation affect the generation of free radicals and the resulting oxidative stress. Nanoparticle toxicology is still an emerging field, with most studies focusing on acute toxicity. Understanding the long-term effects of and chronic exposure to these materials is essential for a comprehensive assessment of their in vivo toxicology.

### Vector screening to improve the efficiency of nucleic acid delivery

The advancement of delivery systems and techniques continues to be the primary challenge in unlocking the vast potential of nucleic acid drugs for gene therapy. Many NPs are composed of diverse chemical materials, and drug delivery mediated by each of these NPs has been evaluated in vitro. Moreover, the quantification of potent vector data in vitro was used to select a minority of NPs for in vivo research. Driven by the need for the systematic design and testing of delivery vehicles, establishing a reliable and efficient in vivo screening method to identify chemical properties that enhance drug delivery. In-depth research has been conducted on the design of LNPs aimed at the efficient delivery of therapeutic RNAs to the lungs via nebulization.^686^ Through the evaluation of the composition, molar ratios, and structure optimization of LNPs, An excellent LNP was found suitable for the nebulized delivery of low-dose mRNA.^686^ This systematic method for LNP design can be modified to optimize LNPs for various administration routes and therapeutic applications. In a related study, researchers synthesized and evaluated a library of 720 biodegradable ionizable lipids to develop inhalable delivery vehicles for mRNAs and CRISPR‒Cas9 gene editors through a high-throughput platform.^687^ Further multistep screening of DNA/LNPs and codelivery with siRNAs provides opportunities for nucleic acid-based gene therapy applications.^688^ This multistep screening platform integrates both in vitro and in vivo strategies to efficiently identify effective LNP candidates from a library of more than 1000 formulations.^688^ Another related advance is the construction of an in vivo library selection platform to optimize the therapeutic performance of protein NPs and protein linkers in living mammals.^689^ This approach may enable the identification of effective delivery platforms for peptide-based gene therapy.

Unlike in monoclonal antibody discovery and engineering, high-throughput sequencing has been utilized to gather extensive data on the diversity of antibody repertoires, and mature materials have been developed.^690^ The underlying mechanism through which NP properties engage with biological systems is unclear, however, it is broadly recognized that these properties are crucial in inducing biological responses..^691^ Moreover, the wide range of different types of NPs and the many unknown factors influencing their formation and biological behavior complicate the design process significantly. It is necessary to screen various combinations of oligonucleotide sequences, component ratios, and target organs to identify the principles that regulate the desired characteristics and therapeutic effectiveness of these agents.^692,693^ These experimental screens involve high workloads and costs, limiting the number of combinations that can be evaluated.^692,694^ Recently, computational models and artificial intelligence techniques have emerged for the design of NP candidates and nucleic acid sequences, accelerating the discovery of new functional drugs.^695,696^ Nevertheless, machine learning algorithms can provide predictive benefits to the speed of screening; aid in understanding physicochemical properties, encapsulation efficiencies, biodistribution and toxicity; and provide great value before synthesizing materials.^697–699^ Using machine learning algorithms, researchers can construct and identify NP-specific biomarkers via genomic NP trafficking networks,^700^ which can be further applied for a quick and high-throughput analysis of multicomponent NP mixtures and solutions.^701^ To effectively implement computational selection methods, there is a need for more reliable data collection tools and better algorithms to enhance formulation scanning throughput.

### Artificial intelligence in small nucleic acid drug discovery

The drug discovery process is complex and includes identifying and validating drug targets, designing and synthesizing compounds, and assessing their efficacy and safety for clinical development. It is estimated that the median cost for discovering and developing a new drug is approximately $985 million, which includes expenses incurred from unsuccessful trials.^702^ The emergence of artificial intelligence (AI)- and machine learning-based methods has led to the introduction of new and rapid algorithms for drug exploration, predicting drug responses and potent drug combinations.^703^ Here, we focus on AI in small nucleic acid drug discovery.

TREAT is an all-in-one platform for target selection, drug design, and optimization. It combines coding and noncoding genes from 81 biological networks under different physiological conditions and employs three advanced algorithms for target ranking and identification. For RNA sequence optimization and siRNA design, TREAT uses user-defined criteria to produce deterministic outputs within a specified search space.^704^ Another model, ‘MysiRNA,’ was trained on 2431 siRNA records and evaluated using three additional datasets. When compared to 11 other scoring tools, MysiRNA demonstrated superior performance based on the correlation coefficient and receiver operating characteristic (ROC) curve, increasing prediction accuracy by as much as 18% compared to the sensitivity and specificity of the best existing tools.^705^ In addition to siRNAs, the sequence and efficiency of ASOs have also received attention. eSkip-Finder (https://eskip-finder.org) is the first online resource that aids researchers in locating available exon-skipping ASOs. This tool facilitates rapid analysis of chosen exon/intron sequences and ASO lengths, leveraging a machine learning model trained on experimental data to identify effective ASOs for exon skipping.^706^

Chemical modifications of the siRNA itself could increase serum stability and target delivery efficiency.^707^ Most recently, a machine learning approach to predicting the efficacy of chemically modified siRNA efficacy has the potential to greatly enhance the siRNA design process for chemical modifications, thereby reducing the time and costs involved in siRNA drug development.^708^ Liu et al. created the Cm-siRPred algorithm utilizing a multiview learning strategy to aid in the design of chemically modified siRNA drugs.^708^ The algorithm utilizes a multiview strategy for predicting the efficacy of chemical modifications to siRNAs and assisting in designing chemical modifications of siRNAs, comprising a module for designing chemical modifications and a module for predicting the efficiency of chemically modified siRNAs.^708^ Furthermore, a model designed to predict the silencing activity of siRNAs featuring different chemical modification patterns has been documented, illustrating the application of machine learning to reveal the correlation between the siRNA sequence, chemical modification pattern, and silencing efficacy.^709^ More importantly, AI and ML integrated with recent advancements in the biomedical or chemical field can significantly accelerate technological progress in healthcare. By exploring the search space of ionizable lipid molecules using the synergy of deep learning and combinatorial chemistry, Xu et al. developed an AI-guided ionizable lipid engineering (AGILE) platform.^710^ The AGILE platform learns structural information for many small molecules through pretrained deep learning neural networks and uses self-supervised methods to discriminate and differentiate lipid structures. After fine-tuning and high-throughput screening, AGILE can accurately identify novel lipid structures with high mRNA transfection efficacy.^710^ Methods that combine systems and synthetic biology with ML models, including graph neural networks, sequence-to-function and sequence-to-structure frameworks, as well as generative models, provide new opportunities for drug candidates and drug discovery approaches.^711,712^ Sebastian M Castillo-Hair and Georg Seelig integrated high-throughput assays and deep-learning techniques to develop predictive models and build quantitative models related to ribosome loading that optimize protein expression for mRNA therapeutic applications.^713^ This strategy is not only quicker but also less likely to become trapped in local sequence optima.^713^

AI depends on the merging of various technologies with basic science methods to leverage extensive multimodal data through predictive modeling to support decision-making.^714^ Through the automated processing and analysis of large-scale data from laboratory and clinical trials, AI not only improves research efficiency but also reduces the likelihood of human error. With the assistance of AI, scientists can identify key data patterns and trends more quickly, thus advancing research more effectively.

### Surface modifications of nucleic acids for delivery to specific cell types

A primary challenge in using NPs for nucleic acid delivery is ensuring selectivity for specific organs or cells, primarily because of the many unpredictable factors that affect how nanoparticles distribute in vivo.^38^ Currently, the clinical efficacy of tissue-specific delivery methods for RNAi therapeutics is mainly limited to hepatocyte targeting, achieved through LNPs (passive targeting), GalNAc conjugates (active targeting), or local administration.^715,716^ For instance, the clinically approved siRNA drug Onpattro can efficiently target the liver when administered through the intravenous route. The GalNAc-siRNA conjugate not only can mediate cellular uptake as a ligand without relying on cationic particles, but also can specifically target hepatocytes.^717^ Thus, approaches to improve the systemic drug delivery efficiency of NPs are highly desirable. A promising strategy involves using targeting ligands and chemical probes, molecules that can specifically bind to surface markers on affected cell populations. This approach is particularly advantageous for single-component delivery systems with well-defined compositions. The RGD peptide can specifically target cancer cells and the tumor vasculature by binding to these integrins. By integrating RGD into lipid‒protamine nanoparticles, Rengaswamy et al. reported a statistically significant tumor growth delay, as well as the inhibition of tumor initiation.^718^ The RVG peptide conjugate, which is specific to the central nervous system, binds to the acetylcholine receptor and can modify various types and forms of nanoparticles for effective delivery to the brain.^719,720^ The addition of RVG to nontoxic SSPEI nanovectors increased microRNA accumulation in the brain by approximately 1.5-fold.^721^ In addition, Tang et al. employed T7, a brain-targeting peptide with a high affinity for the transferrin receptor, to construct lipid-based nanoparticles with DP7 and cholesterol, and the results revealed enhanced brain targeting and extended survival compared with those of T7 unmodified LBNPs.^722^ In addition, novel passive NPs (such as lipid-based, polymer-based, and biomimetic NPs) for organ-selective systemic nucleic acid delivery have made breakthroughs in extrahepatic targeted therapy.^723^ Cheng et al. conceived a strategy to add a fifth molecule to LNPs, which is a selective organ-targeting (SORT) molecule, to establish new compositions without destroying the core 4-component ratios. The results showed that SORT enabled existing liver-targeting LNPs to be tuned to deliver nucleic acids to the spleen or lungs.^724^ Among them, linear PEIs are widely investigated for their capacity to enable lung-selective targeted delivery.^725^ In a novel dry siRNA powder for inhalation, PEI is the delivery vector, and the results showed that a low dose of 3 μg of siRNA resulted in strong and specific gene silencing activity in the lungs without severe lung injury.^726^ A “passive” strategy can be integrated with other methods, such as stimuli-responsive delivery, to attain precise organ selectivity. In addition to NPs, cellular organelles such as extracellular vesicles, mitochondria, lysosomes, and lipid droplets are being actively explored as viable drug delivery systems for therapeutic intervention.^727^ Organelle carriers possess biocompatibility and can be tailored through their specific biological structures or surface modifications to facilitate targeted drug delivery. They predominantly transport drugs to target cells through processes like membrane fusion, receptor-mediated endocytosis, and macropinocytosis.^728–730^ Wei et al. constructed a smart Janus-like surface-coated mitochondrial system that can not only target tumors but also penetrate the depth of tumor tissues and be retained for a long period.^731^ Through the introduction of tumor cell nuclei into activated macrophages, Wang et al. obtained chimeric exosomes with excellent accumulation in both lymph nodes and tumors, which constitute a powerful delivery system for tumor immunotherapy.^732^ Among the different kinds of organelle-based drug delivery systems, exosomes are currently a research hotspot and have entered clinical trials.^733^ The large-scale production of exosomes is challenging and not economically feasible, which also limits the translation of exosomes from the bench to the clinic. Besides the development and screening of NP formulations and drug delivery systems, a fundamental understanding of NP-mediated tissue tropism has also been obtained. Further research is necessary to clarify how these factors contribute to achieving targeted delivery to specific tissues and to refine the design of next-generation delivery systems for clinical use.

### Improved safety profiles

The Oligonucleotide Safety Working Group has issued comprehensive guidelines for evaluating oligonucleotide safety. Unfortunately, trial results have been mixed, with some studies showing strong effects while others report significant adverse reactions. Tolerability issues primarily result from pathogen-associated molecular pattern (PAMP) receptors, like Toll-like receptors (TLRs), recognizing RNA structures or nanoparticles, which trigger adverse immune effects.^69^ An example is the miR-34 mimic MRX34, which resulted in serious treatment-related AEs in five patients with advanced solid tumors in a multicenter clinical trial (NCT01829971).^637^ In preclinical studies, the application of cationic liposomes has been constrained by their toxicity at the administration site.^734^ Therefore, a primary obstacle in bringing nucleic acid therapies to clinical practice and the market is ensuring their absolute safety. By understanding the potential toxicity mechanisms, studies have paid attention to mitigating the adverse effects of NPs.^735,736^ The most commonly reported strategies involve altering the surface chemistry and properties of nanoparticles.^737–739^ Adding biocompatible PEG polymers to SiO_2_ synthesis systems can attenuate chemical toxicity.^740,741^ On the other hand, an innovative strategy to overcome this barrier is to encapsulate NPs in cell plasma membranes, such as membranes obtained from erythrocytes.^742^

For optimal safety, nucleic acid drugs need to ensure that only the antisense strand is selected, which can be achieved by optimizing the thermodynamic stability of the double-stranded RNA.^743^ Sequence selection, including the target site and oligonucleotide length, has decisive effects on both false off-target activities and on-target functions.^744^ Numerous researchers have worked to establish consistent and practical algorithms to increase the chance of oligonucleotide binding success.^745^ In recent decades, certain online design software programs have aimed to improve the quality of siRNA design based on the most highly cited algorithms.^745,746^

### Rational selection of the appropriate target gene

Identifying the correct target gene is essential for ensuring the efficacy of therapy, as it determines the specific genetic pathway to be modulated. Mutations in the superoxide dismutase 1 (SOD1) gene account for approximately 15% of familial ALS cases.^564^ Designing ASO drugs that specifically target these SOD1 gene mutations and reduce the levels of the harmful SOD1 protein represents a promising therapeutic approach.^561^ ASO drugs targeting SOD1 have been used to treat familial ALS in human clinical trials and slow disease progression.^561,747^ This targeted gene selection makes the treatment more focused, significantly improving patient outcomes. In Alzheimer’s disease research, mutations in the amyloid precursor protein (APP) gene are closely linked to the onset of the disease.^748,749^ ALN-APP is a novel RNAi therapeutic developed by Alnylam using proprietary C16-siRNA conjugation technology that targets APP for AD treatment. The conjugation of the siRNA with C16 increases cellular uptake in the central nervous system, and the C16-siRNA binds to the APP mRNA, which is then cleaved and degraded by the RISC, leading to reduced APP protein expression, a subsequent decrease in Aβ protein levels, a further reduction in the level of the substrate for brain amyloid deposition, and an improvement in neurological function. This approach demonstrates how targeting the APP gene with RNAi can intervene at the root cause of the disease, thereby increasing treatment efficacy.

As we move forward, the integration of advanced genomic tools and technologies will further refine this selection process, enabling researchers to identify the most promising genetic targets with greater accuracy. These advancements will be crucial in expanding the range of treatable conditions and in developing personalized therapies tailored to individual genetic profiles. Next-generation sequencing (NGS) and whole-genome sequencing (WGS) have transformed how we identify target genes. NGS provides an extensive view of genetic variations, revealing genes that may be linked to diseases. Similarly, WES focuses on the coding regions of the genome, which are often related to genetic disorders because mutations affect protein function. Bioinformatics tools are essential for interpreting genomic data. Gene Ontology and pathway analyses help researchers associate genes with specific biological functions and processes. Predictive modeling uses computational algorithms to forecast how genetic variations impact protein functions and disease outcomes, assisting in target gene identification. Single-cell genomics adds another layer of detail, providing insights into gene expression at the individual cell level, revealing cellular diversity and identifying specific cell types or states relevant to disease. Furthermore, functional genomics approaches, such as gene knockdown and overexpression studies, are valuable for validating target genes. These methods use RNAi or gene overexpression techniques to evaluate the roles of specific genes in disease models, confirming their potential as therapeutic targets. By integrating these advanced genomic tools and technologies, researchers can more accurately select and validate target genes, paving the way for more precise and personalized treatment strategies.

### Optimizing nucleic acid sequences to reduce off-target effects

For optimal safety, nucleic acid drugs need to ensure the exclusive selection of the antisense strand, which can be accomplished by tuning the thermodynamic stability of dsRNA.^743^ Sequence selection, including the target site and oligonucleotide length, has decisive effects on both false off-target activities and on-target functions.^744^ Clinically utilized siRNAs undergo chemical modifications to increase their potency, minimize immune reactions, and reduce off-target effects (OTEs).^750,751^ A notable example involves the development of 2’-deoxy-2’-α-F-2’-β-C-methyl (2’-F/Me) modifications incorporated into siRNAs, which reduce the thermal stability of double-stranded structures due to steric hindrance. At the ends of oligonucleotides, 2’-F/Me modifications provide greater resistance to nuclease degradation than do 2’-F modifications. Compared with unmodified siRNAs, siRNAs with 2’-F/Me modifications delivered via LNPs or GalNAc showed equal or improved silencing activity in cells and mice. The 2’-F/Me modification at the 7th position of the siRNA antisense strand also reduced the OTEs. When combined with 5’-vinylphosphonate modifications, both the E and Z isomers had similar silencing activities to unmodified siRNA. These findings indicate that the 2’-F/Me modification is a promising tool for increasing the potency, duration, and safety of nucleic acid therapeutics.^752^ The patent CN118202046A by Zhongtian Biotech discloses a strategy for modifying siRNA molecules aimed at reducing OTEs. By introducing PS nucleotide linkages at positions 5–8 in the seed region of the antisense strand, the specificity and silencing efficiency of the siRNA are improved. This modified siRNA can more precisely target and silence HIF1α, thereby reducing OTEs and increasing the safety and efficacy of treatment.

Numerous researchers have attempted to introduce uniform and practical algorithms to increase the chance of oligonucleotide binding success.^745^ In recent decades, certain design software programs have aimed to improve the quality of design based on the most frequently cited algorithms^745,746^; by carefully refining their length, composition, and binding affinity, researchers can increase specificity, ensuring that the nucleic acid sequences bind only to the desired targets. This process involves rigorous testing and computational modeling to predict and eliminate potential off-target interactions. Furthermore, ongoing advancements in predictive algorithms, especially machine learning and artificial intelligence, are increasingly being integrated into bioinformatics platforms, providing powerful tools to identify and assess OTEs before clinical application. These tools allow the simulation of interactions across the entire genome, enabling the selection of sequences with the highest precision and the lowest risk of unintended consequences. OligoWalk, a partition function calculation that considers all possible secondary structures, is used to predict target site accessibility, improving upon methods that consider only the structures with the lowest free energy.^753^ These thermodynamic features, along with siRNA sequence features, are input into a support vector machine to select functional siRNAs.^753^ The method effectively predicts efficient siRNAs (70% efficacy) in a large Novartis dataset, with a positive predictive value of 87.6%.^753^ Very recently, a novel approach called AttSiOff was developed for the prediction of siRNA inhibition and off-target effects. It integrates a self-attention-based siRNA inhibition predictor, an mRNA search package, and an off-target filter. The predictor analyzes siRNA and local mRNA sequences embedded from the pretrained RNA-FM model, capturing key features that influence siRNA inhibition. Tests on five siRNA drugs and a new target gene (AGT) confirmed the practicality and effectiveness of AttSiOff.^754^

Another area of exploration is the development of more comprehensive databases that include a broader range of genomic data, encompassing diverse populations and rare genetic variations. These databases would enable more accurate modeling of potential OTEs, ensuring that therapies are effective and safe across different genetic backgrounds. Novel methods, such as AI-based techniques, may increase the predictive power of algorithms in complex situations.^755^ In the future, collaboration among computational scientists, biologists, and clinicians will be crucial in overcoming these challenges. Together, they can develop next-generation tools that not only predict off-target effects with greater accuracy but also provide strategies to mitigate them.

### Finding new types of small nucleic acid systems

To date, numerous well-studied nucleic acid systems have been selected for clinical therapies, shedding light on the treatment of intractable diseases. In terms of fundamental studies, the discovery of new types of small nucleic acid systems is essential. Several novel systems have been developed, with laboratory data showing encouraging results, positioning them as potential candidates for clinical trials. For example, preclinical experiments have shown that thiourea-based nucleic acid (TNA)-based modifications of ASOs and siRNA, which offer an alternative to natural nucleic acids, exhibit enhanced nuclease resistance compared to conventional 2’-O-methyl or 2’-fluororibose modifications.^756,757^ Additionally, Depmeier et al. pioneered the synthesis of TNA with an expanded genetic alphabet (exTNA), further broadening the scope of TNA-based systems.^758^ Single-stranded oligonucleotides can also be folded into a tetrahedral framework nucleic acid (tFNA). Natural tFNA has inherent reactive oxygen species (ROS) scavenging capabilities and exhibits enhanced structural programmability and efficient endocytosis, thanks to its optimal size and geometry.^759^ Thus, tFNA shows promise as a nanodelivery system for small nucleic acids. For instance, Zhang et al. developed a novel transdermal RNAi drug utilizing tFNA to deliver siRNAs, which demonstrated increased resistance to enzymatic, serum, and lysosomal degradation. In vivo results indicated that this system effectively and specifically silenced the target gene, nuclear factor kappa-B (NF-κB) p65, thereby maintaining the stability of the skin’s microenvironment and restoring normal immune defense.^760^ Moreover, Moreno et al. successfully locked multiple functional siRNAs into a bimolecular “caged-siRNA” structure. This structure, formed from single-stranded siRNA and a DNA dendron, self-assembles through the identification and pairing of sense and antisense strands. Their findings revealed that this caged-siRNA system, with multiple RNAi triggers, holds significant potential for therapeutic applications.^761^
